# An integrated miRNA functional screening and target validation method for organ morphogenesis

**DOI:** 10.1038/srep23215

**Published:** 2016-03-16

**Authors:** Ivan T. Rebustini, Maryann Vlahos, Trevor Packer, Maria A. Kukuruzinska, Richard L. Maas

**Affiliations:** 1 Division of Genetics, Department of Medicine, Brigham and Women’s Hospital, Harvard Medical School, Boston, MA 02115, USA; 2 Department of Molecular and Cell Biology, Boston University Medical Center, Boston, MA 02118, USA

## Abstract

The relative ease of identifying microRNAs and their increasing recognition as important regulators of organogenesis motivate the development of methods to efficiently assess microRNA function during organ morphogenesis. In this context, embryonic organ explants provide a reliable and reproducible system that recapitulates some of the important early morphogenetic processes during organ development. Here we present a method to target microRNA function in explanted mouse embryonic organs. Our method combines the use of peptide-based nanoparticles to transfect specific microRNA inhibitors or activators into embryonic organ explants, with a microRNA pulldown assay that allows direct identification of microRNA targets. This method provides effective assessment of microRNA function during organ morphogenesis, allows prioritization of multiple microRNAs in parallel for subsequent genetic approaches, and can be applied to a variety of embryonic organs.

The growing appreciation of the role of microRNAs (miRNAs) in regulating organ morphogenesis^1^ underscores the need for methods that rapidly assess miRNA function *in vivo*. Methods for *in vivo* miRNA functional perturbation include the injection of chemically modified miRNA inhibitors^2^, referred to here as antagomirs, and viral infections of DNA constructs expressing miRNA inhibitors^3^, both of which are limited to postnatal developmental stages and can generate systemic off-target effects. The *in vivo* genetic targeting of miRNAs using knockout mouse models^4^ remains a gold standard to address miRNA function during organogenesis, but is costly and time-consuming.

Explanted mouse embryonic organs provide a reliable and reproducible alternative to mouse genetic models, and in terms of suitability for rapid screening, offer potential advantages. Organ explants of submandibular salivary glands (SMGs)^5^, lungs^6^, and kidneys^7^, mammary glands^8^, and tooth^9^ recapitulate some of the important morphogenetic processes involved in early organogenesis, including epithelial proliferation and branching morphogenesis. In addition, organ explants can be used to visualize organ development in real-time^7^,^10^, and provide three-dimensional models for developmental and regenerative biology.

The mechanism of miRNA action is based on the specificity of its 5′-UTR seed region, a 6–8 nucleotide sequence that binds to the complementary 3′-UTR sequence present in the corresponding target mRNA, which triggers mRNA degradation and translational downregulation^11^,^12^. Antagomirs are complementary oligonucleotides to mature miRNAs that prevent interactions between targets and their corresponding miRNAs. Methods to transfect antagomirs have frequently employed liposomes^13^,^14^ and have largely been limited to *in vitro* applications in cell lines^15^. Antagomirs can incorporate a chemical modification termed Locked Nucleic Acid (LNA)^2^,^15^, which consists of a 2′, 4′ methylene-bridge in the ribose that forms a bicyclic nucleotide with higher affinity binding to the complementary miRNA target. This allows the use of short LNA-modified oligonucleotides in multiple applications including miRNA *in situ* hybridization^16^ and knockdown studies^15^. The efficiency of liposome-based LNA-modified antagomir transfection, previously used to interfere with miRNA function in explanted embryonic SMGs^17^, can be affected by the cytotoxicity of the liposomes, triggering stress-induced off-target effects and the degradation of the antagomir (or any other cargo molecule carried by the liposomes) via the endocytic pathway^18^.

An alternative method of transfection originally used in siRNA gene targeting^19^ employs peptide-based nanoparticles to overcome the problems of liposome cytotoxicity and endocytic degradation. Cell-penetrating amphiphilic peptides that possess a self-assembling property have relatively higher affinity for single and double strand nucleic acids compared to liposomes. To date, the commercially available N-TER peptide (Sigma^TM^)^19^ is the most commonly used peptide for nucleic acid delivery in a variety of applications, including transfection of antagomirs in cell lines^20^. Despite their versatility, to our knowledge nanoparticle-forming peptides have not been previously used to transfect miRNA antagomirs and mimics into more complex systems such as explanted embryonic organs.

Here, we describe a method to rapidly characterize the developmental effects of individual miRNAs during organ morphogenesis (Fig. 1). First, we employ peptide-based nanoparticles to transfect specific miRNA antagomirs and mimics for each miRNA to be evaluated into embryonic organ explants to test for loss- or gain-of-function effects, respectively (Fig. 1a; Suppl. Fig. 1). Second, for miRNAs that yield interesting phenotypes or gene expression changes, we apply a direct miRNA target validation assay based on biotinylated miRNA mimics and miRNA pulldown (Fig. 1b; Suppl. Fig. 2).

An initial step in this protocol involves selecting the miRNAs for functional studies. It has been demonstrated that miRNAs play essential roles in directing endoderm, mesoderm, and ectoderm specification and differentiation during organogenesis^21^. Therefore, one approach is to identify or generate datasets of differentially expressed miRNAs in representative ectodermal (*e.g.,* tooth germs and SMGs), endodermal (*e.g.,* lungs), and mesodermal (*e.g.,* kidneys) organs during early organogenesis, based on their relative abundance within an individual organ (expression profile), or after comparing miRNA expression among different organs to identify a set of miRNAs that are enriched in a specific organ (expression signature).

Either RNA-Seq^22^,^23^ or qPCR-based TaqMan Low Density Arrays (TLDA)^24^ can enable fast and robust miRNA expression screening, and have generated miRNA expression databases for different developing vertebrate organs^1^,^17^,^22^,^25^,^26^. Starting from any of these miRNA expression datasets, bioinformatic and annotation-based criteria must be employed to select miRNAs for further evaluation. Herein, we provide a rapid, first-order functional screening method that can be applied to a variety of vertebrate organs that develop via epithelial-mesenchymal interactions. For illustrative purposes, we focus on the murine molar tooth germ and the SMG as representative organs, since both provide classical, well-established model systems^5^,^8^,^9^,^27^ to investigate early morphogenetic processes during organ development.

## Results

### Selection of miRNAs for organ-specific perturbation

**Figure 2:**
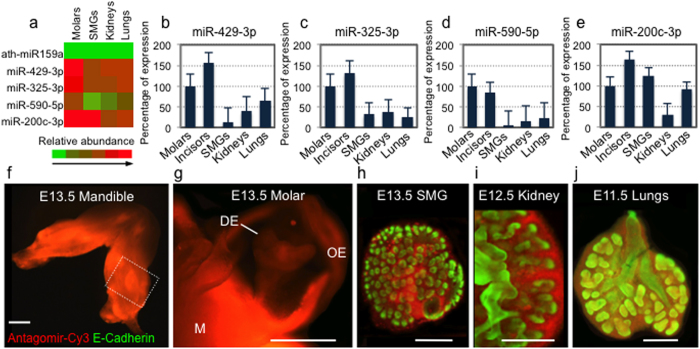
Selection of miRNAs for functional perturbation and visualization of antagomir transfections into organ explants. (**a**) Heat map of miRNA expression generated by TaqMan Low Density Array (TLDA) analysis shows a negative control miR from *Aradopsis* (ath-miR159a) and four miRNAs that are part of a miRNA expression signature for molar tooth germs. (**b–e**) qRT-PCR graphs show percent relative gene expression (normalized to molars) for miR-429-3p, miR-325-3p, and miR-590-5p in embryonic molar tooth germs, incisors, submandibular salivary glands (SMGs), kidneys and lungs. miR-200c-3p, a known regulatory component of the morphogenetic processes in embryonic tooth and SMG, is also included. (**f–j**) Representative epifluorescent images were collected after 48 h of *ex vivo* culture using 100 nM of Antagomir-Cy3 (red) transfected into intact E13.5 mandible explants (**f**), after dissecting the epithelium and mesenchyme tissues with Dispase incubation (**g**), or after fixation of the organ explants and whole mount immunofluorescence for E-Cadherin (green) (**h–j**). The epithelial-mesenchymal separation (**g**) suggests efficient nanoparticle-based transfection of Antagomir-Cy3 throughout both the oral and dental epithelia (OE and DE, respectively) and in the mesenchyme (M), a result confirmed quantitatively by flow cytometry analysis of isolated dissociated epithelial and mesenchymal cells after Antagomir-Cy3 transfection into mandibular explants (see Suppl. Fig. 5). Scale bars: 100 μ.

We generated miRNA datasets using total RNA from embryonic molar tooth germs, salivary glands, lungs, and kidneys, and performed TLDA analysis of miRNA expression. We then obtained relative miRNA expression profiles using a qRT-PCR approach^28^ (Suppl. Table 1). Our TLDA analysis of miRNA expression (Fig. 2a) revealed enriched expression of miR-429-3p, miR-325-3p, and miR-590-5p in molar tooth germs and developing incisors compared to SMGs, lungs and kidneys, which we confirmed using individual primers to detect the corresponding miRNAs (Fig. 2b–d; Suppl. Fig. 3b). We also generated miRNA signatures that could potentially be useful for miRNA perturbation studies using other embryonic organs (Suppl. Table 1).

In addition, to help validate our miRNA functional perturbation method, we included in this analysis the miR-200c-3p (Fig. 2e), which although not differentially expressed in molar and incisor tooth germs compared to SMGs and lungs, is known to play a role in regulating epithelial proliferation during odontogenesis^29^ and SMG branching morphogenesis^17^.

### miRNA screening in explanted organs

#### Efficiency of antagomir transfection into organ explants

To develop a rapid, reproducible protocol for perturbing miRNA function during organ morphogenesis, we first optimized nanoparticle transfection efficiency using a Cy3 fluorescently-labeled off-target antagomir (Antagomir-Cy3) to visualize antagomir uptake in embryonic mandible, SMG, kidney, and lung explants (Fig. 2f–j). We dissected the epithelium and the mesenchyme from mandibular explants, and found that Antagomir-Cy3 uptake was qualitatively detected in both tissues after nanoparticle transfections (Fig. 2g). We found that Nanoparticle-Forming Solution (NFS) was comparatively more efficient than Liposome-Forming Solution (LFS) in transfecting Antagomir-Cy3 into explanted E13.5 SMGs and E11.5 lungs cultured on Nuclepore floating filters (Suppl. Fig. 4a,b). As an additional quantitative measure of Antagomir-Cy3 transfection efficiency in molar tooth germs, we prepared single cell suspensions from dissected epithelium and mesenchyme dental tissues after Antagomir-Cy3 nanoparticle transfection into embryonic mandibular explants, and subjected them to flow cytometry analysis (Suppl. Fig. 5). The results showed that ~81% of epithelial cells and ~93% of mesenchymal cells were transfected with Antagomir-Cy3. In addition, cell viability assays did not show significant cell death when comparing Antagomir-Cy3 nanoparticle transfection with that of an off-target, unlabeled (negative control) antagomir, or with untreated explanted embryonic mandibles (Suppl. Fig. 5).

#### Efficacy of antagomir transfection

**Figure 3:**
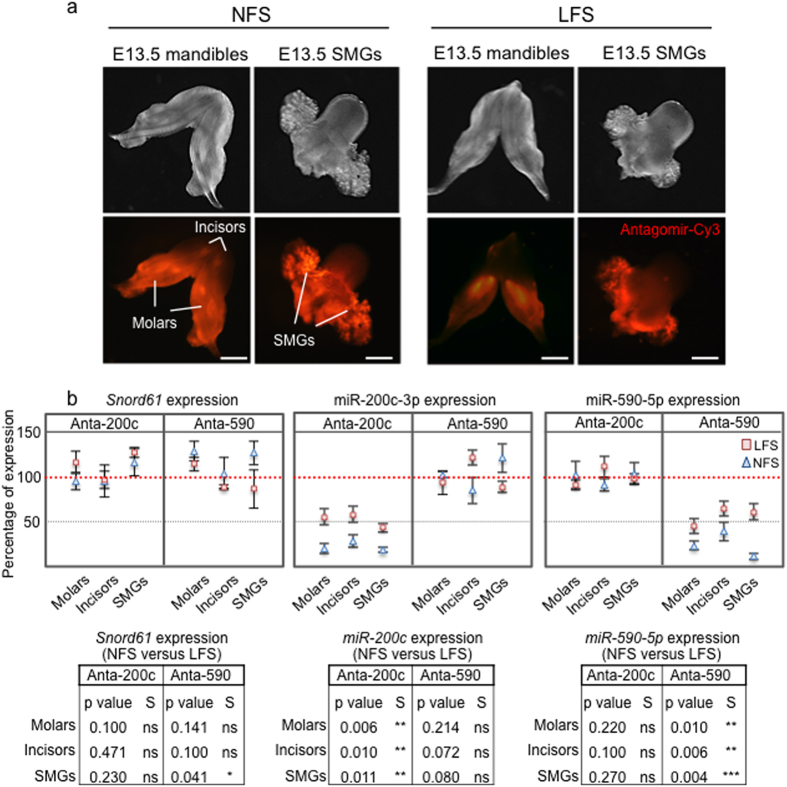
Efficiency and specificity of miRNA knockdown using nanoparticle or liposome based antagomir transfection. (**a**) Representative images of explanted E13.5 mandibles and SMGs (attached to tongue) in Trowell-type organ culture after 48 h of transfection of a fluorescently labeled antagomir (Antagomir-Cy3) using Liposome Forming Solution (LFS) or Nanoparticle Forming Solution (NFS) methods. Images correspond to 4–6 representative organ explants with experimental triplicates. Antagomir-Cy3 uptake was more efficient with NFS than LPS (for quantification of Antagomir-Cy3 uptake, see Supplementary Fig. 5). Scale bar: 100 μ. (**b**) Antagomirs targeting miR-590-5p and miR-200c-3p (Anta-590 and Anta-200c, respectively), were transfected into E13.5 mandible and SMGs (attached to tongue) in Trowell-type organ cultures. Molar and incisor tooth germs, and SMGs were dissected after 24 h, and miRNA expression assessed by qRT-PCR. Both NFS and LFS showed specific miRNA knockdown after transfection of the respective antagomir (middle and right graphs). *Snord61* expression was used as an off-target control (left graph), and the qRT-PCR data were normalized to snRNA-U6 expression and plotted as percentage of expression compared to the control (represented here as 100% of expression, dotted red line). Expression differences in miRNAs after transfection of the corresponding antagomirs using LFS (pink squares) or NFS (blue triangles) are statistically significant (T-Student paired one tailed test), as follows: miR-200c-3p expression in molar tooth germs (p = 0.006), in incisors (p = 0.010), and SMGs (p = 0.011) using Anta-200c (graph in the middle); miR-590-5p expression in molar tooth germs (p = 0.010), in incisors (p = 0.006), and SMGs (p = 0.004), using Anta-590 (graph on the right). *Snord61* expression did not change significantly in most treatments. For further data documenting the specifity of antagomir treatment, see Suppl. Figs 7 and 8.

To establish the efficacy of antagomir in decreasing expression of its corresponding miRNA, we conducted a comparative analysis using liposome (Liposome-forming solution, LFS)^17^ and peptide-based nanoparticle (Nanoparticle-forming solution, NFS) methods of transfection (Fig. 3a,b). We selected antagomirs targeting miR-590-5p, previously defined as a component of the molar tooth germ miRNA expression signature (Fig. 2d), and miR-200c, a known regulatory component of tooth^29^ and SMG^17^ morphogenesis. After transfecting antagomirs targeting miR-200c-3p and miR-590-5p in explanted E13.5 mandibles, or in explanted E13.5 SMGs attached to the tongue (Fig. 3a), the analysis of miRNA expression by qRT-PCR showed a selective decrease in miRNA expression using both LFS and NFS transfections compared to an irrelevant arbitrarily selected target small nucleolar RNA *Snord61* and *snRNA U61* (Fig. 3b). The decreases in miR-200c expression (middle graph, Fig. 3b) using the corresponding antagomir transfected with LFS (pink squares) and NFS (blue triangles) were, respectively: 42.0% and 81% (molar tooth germs), 43.0% and 72.0% (developing incisors), and 36.0% and 80.0% (SMGs). The decreases in miR-590-5p expression (right graph, Fig. 3b), using antagomirs transfected with LFS (pink squares) or NFS (blue triangles) were, respectively: 39.0% and 77.0% (molar tooth germs), 33.0% and 61.0% (developing incisors), and 40.0% and 81.0% (SMGs). Transfections of antagomirs using NFS were significantly more efficient than LFS in decreasing expression of the target miRNA. Thus, in these initial experiments the organ explant antagomir transfection method shows a relatively high miRNA targeting efficiency.

#### Specificity of antagomir induced miRNA targeting

To evaluate the specificity of the antagomir-induced downregulation of target miRNAs, we next conducted a comprehensive qRT-PCR-based expression survey of odontogenic miRNAs and their targets (Suppl. Figs 7, 8). We also evaluated the specificity of miRNA targeting using individual antagomirs in increasing the expression of their corresponding target genes (Suppl. Fig. 8). We compared the expression of a set of odontogenic miRNAs using qRT-PCR, after transfection of antagomirs in mandible explants that targeted miR-590-5p and miR-21 (Suppl. Fig. 7), since members of the miR-21/miR-590-5p family partly share seed sequence identity^30^. We found specific and statistically significant miR-590-5p and miR-21 downregulation after transfection of the respective antagomirs (Suppl. Fig. 7), whereas the expression of other odontogenic miRNAs did not change.

To furher evaluate the specificity of miRNA perturbation, we screened a selection of 15 odontogenic and morphogenetic marker mRNAs (Suppl. Table 3) in molar tooth germs after transfection of antagomirs targeting the previously defined miRNA-signature in mandible explants (Suppl. Fig. 8a). We found specific upregulation of miR-590-5p odontogenic targets after transfection with the corresponding antagomir (Suppl. Fig. 8b), increased expression of *Nog* (a miR-200c-3p target)^31^ after transfection of miR-200c antagomir, and *Pdcd4* (a miR-21 target)^32^ and *Casp3* (which is known to be upregulated in the miR-21 knockout mouse)^33^ after transfection of miR-21 antagomir.

#### Developmental assays following miRNA perturbation

**Figure 4:**
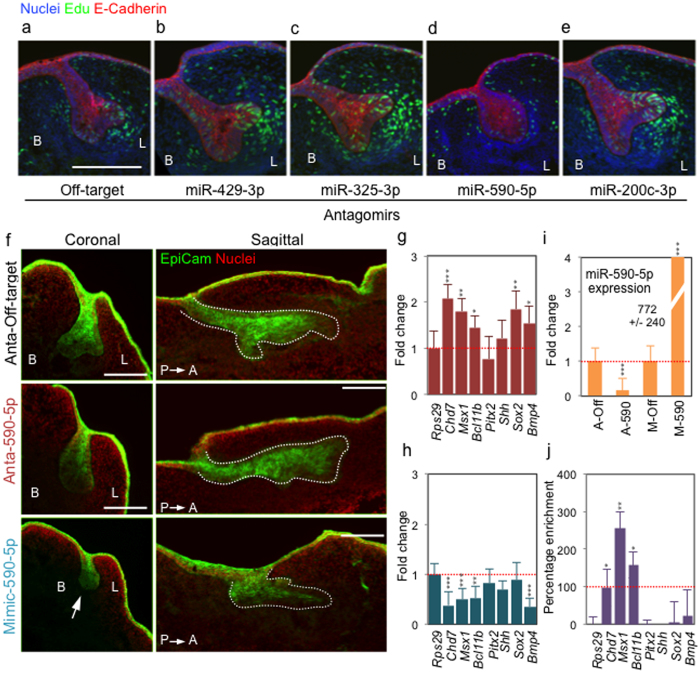
Functional screening of miRNAs in molar tooth germ explants and validation of miRNA target genes. (**a–e**) Representative molar tooth germ phenotypes generated by miRNA antagomir transfections into mandibular explants. Coronal sections (10 μ) of molar tooth germs show nuclear labeling with DAPI (blue), epithelial E-cadherin immunostaining (red), and EdU incorporation (green). Note increased proliferation (EdU) in the lingual (L) side to the tooth germ (in **b**,**c**,**e**), and the progression of epithelial morphogenesis (from cap to bell stage) after targeting miR-429-3p (**b**), miR-325-3p (**c**), and miR-200c-3p (**e**), whereas epithelial proliferation and morphogenesis were inhibited with miR-590-5p antagomir transfection, compared to off-target antagomir (**a**). (**f**) Coronal and sagittal sections of E13.5 mandible explants showing molar tooth germ epithelium (EpiCam, green) and nuclei (DAPI, red). Defects in molar tooth germ morphogenesis after 48 h of antagomir transfection targeting miR-590-5p (Anta-590) include lack of the normal cap stage morphology observed in the Anta-off-target control, and arrest of tooth morphogenesis at the bud stage after transfection of the miRNA-590-5p mimic (arrow in the lower panel). B and L correspond to buccal and lingual orientation, and P and A to posterior and anterior orientation, respectively. Scale bar: 100 μ. (**g–h**) qRT-PCR analysis shows significantly increased expression for the miR-590-5p targets *Chd7*, *Msx1*, and *Bcl11b*, 24 h after antagomir transfection (**g**), and corresponding decreases in expression after mimic transfections (**h**). Graphs represent fold-change in gene expression normalized to the control housekeeping gene *Rps29* and to an off-target antagomir transfection group (red dotted lines). (**i**) qRT-PCR analysis shows decreased fold change in miR-590-5p expression after transfection of the corresponding antagomir (A-590), and increased detection of miR-590-5p after mimic transfection (M-590). A-Off: Antagomir off-target; M-Off: Mimic off-target. Graph shows relative fold-change in qRT-PCR expression, normalized to control non-coding RNA (*snoRD61*) and to an off-target antagomir control (red dotted line). (**j**) Enrichment of miR-590-5p targets in the pulldown fraction after transfecting a biotinylated miR-590-5p mimic in E13.5 mandible explants (24 h), and performing the pulldown (miR-PD) assay. Graph shows relative percent enrichment for miR-590-5p predicted targets *Chd7*, *Msx1*, and *Bcl11b,* compared to non-target genes (*Shh*, *Sox2*, *Bmp4*). Details on qRT-PCR data normalization are in Suppl. Fig. 2.

After transfecting individual antagomirs into embryonic mandibular explants, and once the knockdown efficacy and specificity of miRNA targeting were established (Fig. 3), we next performed several different developmental assays, including cell proliferation, and morphological and histological assessment after targeting various odontogenic miRNAs (Fig. 4a–e). We prepared frozen sections of antagomir transfected embryonic mouse mandibular explants, followed by E-Cadherin and EpCam immunohistochemistry detection to assess epithelial morphogenesis, or EdU detection following EdU labeling of cultured explants to assay cell proliferation.

We found that miR-590-5p perturbation produced abnormal cap stage formation during molar tooth germ morphogenesis (Fig. 4d,f, upper panel), and that miR-200c-3p loss-of-function via its antagomir (Fig. 4e) increased proliferation in both epithelial and mesenchymal tissues, as previously observed in SMG^17^ and in tooth morphogenesis in a miR-200c knockout mouse^31^. Of note, loss-of-function using antagomirs targeting other miRNAs previously defined as part of the odontogenic miRNA signature (miR-429-3p and miR-325-3p, Fig. 4b,c) also produced increased epithelial and mesenchymal proliferation, compared to an antagomir off-target control.

In a potentially more rapid approach, embryonic mandibular explants were isolated from transgenic or knockin mouse strains that express fluorescent reporters under the control of promoters for key regulatory genes for tooth morphogenesis (*e.g.,* Krt14-GFP, Shh-GFP), and analyzed directly by fluorescence microscopy (Suppl. Figs 12, and 13). These analyses revealed disorganized epithelial morphology triggered by miR-590-5p antagomir in short-term mandibular explants (Suppl. Fig. 12). Interestingly, when explanted organs were cultured for longer period of times, these differences in molar tooth germ morphologies no longer attained statistical significance (Suppl. Fig. 13). This suggests potential developmental compensation for miR-590-5p function. In addition, most changes in miR-590-5p target genes using antagomirs and mimics, although statistically significant, are small (Fig. 4g–h), suggesting a fine-tuning rather than a major regulatory role for miR-590-5p.

### miRNA target prediction and validation using miRNA pulldown

Having established that antagomir transfections produce a reproducible effect in embryonic mandibular explants, we sought to extend the miRNA loss-of-function screening approach by: (i) predicting and validating potential miRNA target genes, and (ii) transfecting the corresponding miRNA mimic into organ explants and assessing the developmental effect,, with the possibility of observing a complementary and/or distinct phenotype. Notably, the developmental effects obtained by using antagomirs and mimics are not necessarily expected to be complementary (see Discussion).

To assist in this goal, we developed a target prediction pipeline (Suppl. Fig. 6) that utilizes TargetScan^34^, a searchable homology-based prediction database that associates miRNA seed regions with 3′-UTR sequences of potential target mRNAs. Our target prediction pipeline employed two selection filters: (i) the presence of the potential miRNA targets in the organ of interest using an appropriate gene expression database (*e.g.,* ToothCODE for molar tooth germ^35^; Suppl. Table 2); and (ii) target gene interaction analysis, using the Gene Ontology-based CytoScape-GeneMania plugin^36^ (Suppl. Table 2). We detected several miR-590-5p targets potentially associated with the developing molar tooth germ (Fig. 4g,h, and Suppl. Fig. 6), such as *Pitx2*, *Bcl11b*, *Msx1*, *Chd7*, and *Edar*. Each of these genes was associated with the GO category “Odontogenesis” (Suppl. Fig. 6, and Supp. Table 2).

The abnormal molar germ tooth morphogenesis caused by the transfection of miR-590-5p antagomir (Fig. 4d), and the target prediction associated with odontogenesis (Suppl. Fig. 6) provided a rationale to focus on its functional analysis. We transfected a miR-590-5p mimic into E13.5 mandibular explants in Trowell-type organ culture, and assessed gene expression after 24 h, and morphogenesis after 48 h. The transfection of miR-590-5p mimic in the molar tooth germs promoted arrest in molar morphogenesis at the bud stage (Fig. 4f, lower panel). To complete the morphogenetic analysis, we assessed the expression of the potential targets predicted by the bioinformatic pipeline, and changes in miR-590-5p expression using qRT-PCR. As expected, expression of the predicted miR-590-5p targets *Chd7*, *Msx1*, and *Bcl11b* (Fig. 4.g) significantly increased after antagomir transfection, and decreased after mimic transfection (Fig. 4h), whereas miR-590-5p detection significantly decreased with antagomir, and increased with mimic transfections (Fig. 4i).

As a final important additional feature of this protocol, in select cases where developmental assays revealed compelling and consistent phenotypes, we sought to biochemically validate predicted miRNA targets. From a number of methods to investigate miRNA and mRNA interactions (Suppl. Table 4), we developed a biotinylated miRNA mimic pulldown assay (miR-PD) to directly assess binding of miR-590-5p to its predicted odontogenic targets by determining whether its target mRNAs were enriched in the pulldown fraction (Suppl. Fig. 2). We transfected biotinylated miR-590-5p mimic into E13.5 mandibular explants, dissected the molar tooth germs for subsequent miR-PD assay, and detected significant enrichment for *Chd7*, *Msx1*, and *Bcl11b* mRNAs in the pulldown fractions using biotinylated miR-590-5p (Fig. 4j). In contrast, co-regulated genes involved in molar tooth germ morphogenesis such as *Shh*, *Sox2*, *Bmp4* were not enriched. We also found that *Pitx2*, a predicted miR-590-5p target, did not enrich in the pulldown fraction. Thus, the miR-PD assay may distinguish direct miRNA targets from mRNAs that might be simply co-regulated with target genes, or that are indirectly influenced by miRNA functional perturbations.

## Discussion

To devise a rapid method for identifying miRNAs with significant effects on organ morphogenesis, we developed a miRNA functional perturbation protocol that reliably reproduces expected effects on target miRNA expression, and as a result, on the predicted target mRNAs and on organ morphogenesis. Flow cytometry of epithelial and mesenchymal tissues that were enzymatically separated from mandibular explants transfected with fluorescently labeled antagomirs and dispersed to single cells revealed 80–90% antagomir uptake into both tissues. The fact that such a high proportion of organ explant cells are transfected means that intact explants should include a sufficient majority of treated cells to yield representative and reproducible effects on gene expression and morphology, as was observed. In addition, although it was necessary to use ~10x higher concentrations (50–100 nM) of antagomirs or mimcs to load nanoparticles or liposomes for transfection in organ explants than the concentrations typically employed in cultured cells (~10 nM), we did not find any obvious evidence of off-target effects. Although off-target effects are a well-known corollary of antagomir and mimic based perturbations, large-scale, quantitative gene expression experiments (*e.g.,* RNA-Seq) would likely be required to detect their existence. Since we did not detect obvious off-target effects among a panel of 15 miRNAs, and since the main purpose of this method is to prioritize miRNAs for futher *in vivo* analysis, we conclude that off target effects are not likely to be especially problematic.

In addition to changes in gene expression, we applied a series of developmental assays, including marker and flourescent transgenic reporter expression, EdU incorporation, and morphologic and histologic changes, to prioritize miRNAs for subsequent in depth evaluation via *in vivo* mutagenesis. Lastly, in some cases, using similar methods as for antagomir and mimic transfection, it is possible to use transfected biotinylated mimics in a streptavidin bead pull down assay to interrogate potential direct miRNA targets. Thus, the assay as developed is versatile, multi-faceted and capable of rapidly yielding abundant preliminary information on the function of indvidual miRNAs in organogenesis.

We chose to survey organ explants that recapitulate early morphogenetic processes in *ex vivo* culture using two systems: (i) explants of individual embryonic organs on Nuclepore floating filters^6^,^27^,^37^, and (ii) use of the classic Trowell-type organ culture system^8^. In our experience, epithelial branching organs were best studied using Nuclepore floating filters, while the early morphogenetic events of odontogenesis, when detailed histology was required, were best assessed using the Trowell-type organ culture. In a refinement of this method, we explanted embryonic mandibles directly on top of a metal grid without Nuclepore filters (Suppl. Fig. 9). This final streamlined procedure eliminated the need to dissect the tooth germs from the mandible, preserved their anatomical orientation, and facilitated the preparation of frozen sections.

In our explanted embryonic mandible cultures, we assessed the morphologic changes in molar tooth germs, starting from the E13.5 bud stage, continuing through the E14.5 cap stage, and concluding at the E15.5–16.5 bell stage^9^, using mandible frozen sections and immunofluorescence. Of note, when transfected with an off-target antagomir control (Fig. 4a) and analyzed after short-term (48–72 h) culture conditions, explants showed a normal developmental progression, including transition from epithelial bud to cap (48 h) and/or bell (72 h) stages, with proliferation in both dental epithelium and mesenchyme. Furthermore, we analyzed cell viability after antagomir transfections, using epithelial and mesenchymal single-cell suspensions and flow cytometry (Suppl. Fig. 5), and comparing two independent LNA-modified antagomir controls (Cy3-labeled and unlabeled off-target sequences) with untreated mandible explants, and did not find significant differences. All together, and in agreement with the negligible off-target efects previously described in applications using other types of LNA-modified oligos^38^,^39^, our results suggest that the off-target effects of LNA-antagomir treatments did not affect, at short-term, the morphogenetic processes that we set out to investigate.

In contrast to the results obtained after off-target antagomir transfection, mandibular explants, transfected with antagomirs targeting miR-429-3p, miR-325-3p, miR-590-5p, or miR-200c-3p exhibited striking changes in molar germ morphogenesis (Fig. 4a–e). miR-200c-3p antagomir increased epithelial molar tooth germ proliferation (Fig. 4e), in agreement with previous experiments that genetically removed miR-200c during murine tooth morphogenesis^31^. Although miR-590-5p expression was significantly downregulated after 24 h of antagomir transfection in molar tooth germ explants (Fig. 4i), caution must be used when analyzing the apparent increase in miR-590-5p expression after mimic transfections (Fig. 4i), since the exogenously transfected mimic and the endogenously expressed miR-590-5p sequences are indistinguishable by qRT-PCR. Regardless, we demonstrated a dose-response relationship for transfected mimics (Suppl. Fig. 10), and concentrations of mimics lower than 50 nM did not significantly alter the expression of the corresponding miR-590-5p targets (not shown).

The arrest in molar tooth morphogenesis caused by miR-590-5p mimic transfection (Fig. 4f, lower panel), resembles the phenotype of *Msx1* knockout mice^40^. This result is therefore consistent with the observed statistically significant decrease in expression of *Msx1* (Fig. 4h), a predicted miR-590-5p target gene. Of note, although endogenous miR-590-5p is preferentially expressed in molar tooth germ epithelium (Suppl. Fig. 3c) where it presumably contributes to the general downregulation of *Msx1* in that tissue, mimic transfection is expected to distribute into both epithelium and mesenchyme, thereby downregulatimg *Msx1* expression in the latter. Conversely, compared to off-target control (Fig. 4f, upper panel), miR-590-5p antagomir transfection produced a disorganized epithelium with no visible cap or bell stage morphologies or enamel knot formation (Fig. 4f, middle panel). These defects could reflect up-regulation of the miR-590-5p target genes *Chd7* and/or its co-factor *Sox2*^41^ in the epithelium (Fig. 4g, Suppl. Fig. 11). Both genes, which encode interacting co-factors, are required for dental epithelial proliferation, and their up-regulation would be consistent with a block in dental epithelial differentiation. These results thus suggest a functional role for miR-590-5p in early molar tooth development, and illustrate how this method can provide a potentially useful first-order tool for miRNA functional assessment.

As a final feature of this method, the pulldown protocol using biotinylated miRNA mimics (miR-PD)^42^ provides a straightforward method to validate miRNA targeting, when compared with other methods currently available such as Luciferase Reporter Assays^43^, Ribosome Profiling^11^, and Cross-Linking Immuno-Precipitation or CLIP^44^ (Suppl. Table 4). The predicted miR-590-5p targets *Chd7*, *Msx1*, and *Bcl11b*, which contain seed regions for miR-590-5p (Suppl. Fig. 14) were enriched in the pulldown fraction (Fig. 4j), whereas presumptive non-target genes such as *Shh*, *Sox2*, and *Bmp4* were not. Possible explanations for discrepancies between target prediction and validation, exemplified by the case of *Pitx2* which was not enriched in the pulldown fraction (Fig. 4j), include potential false-positives associated with all miRNA target prediction methods^45^, and the possibility that the *Pitx2* 3′-UTR region may not be physically exposed or available to allow binding due to secondary structure.

In sum, the combined use of miRNA loss- and gain-of-function and direct target validation provides a practical first-order functional assessment of selected miRNAs in embryonic organ explants. The versatility of embryonic organ explants allows verification of morphogenetic changes when interfering with miRNA function in real-time, which can be challenging using *in vivo* approaches. Potential off-target effects may occur, but our experiments suggest that these are not highly prevalent, and in any case need not interfere with the goal of this method to prioritize miRNAs for more faithful gene targeting approaches where such non-specific effects can be definitively addressed. Other advantages of the method include an improved transfection efficiency using nanoparticles, rapid gene expression analyses using qRT-PCR, a multiwell plate configuration that allows up to 12 different treatments (antagomirs and mimics) at a time and that it is scalable according to the size and the number of organ explants, the use of accessible, inexpensive reagents, the use of miR-PD as an efficient miRNA target validation method that requires only small amounts of RNA for qRT-PCR analysis (when screening for a selected number of predicted targets), and the convenient use of biotinylated mimics in both gain-of-function and pulldown assays.

At the same time, some potential limitations of this protocol exist. These include the limited lifespan of explanted embryonic organs and the potential developmental artifacts associated with organ culture, the inherent inaccuracy of miRNA target gene prediction programs that can generate false positives or exclude authentic targets, and the small amounts of total RNA available for miR-PD assays, which may require scaling up experiments or increasing the RNA yields when unbiased and high throughput analyse of gene expression such as RNA-Seq are required. Nonetheless, the method described here provides rapid, efficient first-order approach to assess miRNA function in several embryonic mouse organs, and in some cases, enables the simultaneous identification of miRNA target mRNAs. Furthermore, the method is scalable to moderate throughput, as organ explants can be adapted to multi-well formats and scored for developmental phenotypes in parallel. While the gold standard for the assessment of gene function remains targeted gene inactivation *in vivo,* even CRISPR^46^ and TALEN-mediated^47^ gene editing methods can be expensive and time-consuming. The method described here can instead serve as a highly effective and low cost first-order screening plaform for prioritizing miRNAs for futher in depth investigation.

## Methods

### Embryonic organ dissections

The methods were carried out in accordance with the guidelines and regulations of the protocol 750-R98 approved by the Harvard Medical School Institutional Animal Care and Use Committee. The embryonic stages for organ dissections were E11.5 for lungs, E12.5 for kidneys, E13.5 for salivary submandibular glands (SMGs), and E13.5 for molar germs, which represent the starting times for the corresponding explants (Suppl. Fig. 3a). Briefly, pregnant euthanized CD-1 mice were surgically open, the uterus from each mouse containing the embryos was removed and placed in a 150 mm Petri dish containing 15–20 mL of DMEM-F12/PS as previously described^27^. Embryos were dissected from the uterus, washed twice with DMEM-F12/PS, and further dissected under a stereomicroscope according to specific methodologies described for harvesting molar tooth germs^48^, SMGs^27^, lungs^6^, and kidneys^37^. Embryonic organs were used for explant organ cultures or total RNA extraction.

### Total RNA extraction

Each collection of embryonic organs (Suppl. Fig. 3a), was subjected to total RNA extraction using mirVana™ total RNA Isolation Kit (Ambion). Total RNA was quantified using a Nanodrop Spectrophotometer (ThermoFisher), distributed in aliquots, immediately frozen in dry ice, and stored at −80 °C prior to use.

### Screening of miRNA expression using TaqMan Low-Density Arrays

Reverse Transcription (RT) reactions were prepared by pipetting 500–1,000 ng aliquots of total RNA from each embryonic organ of interest in 0.7 mL PCR tubes, and pipetting the reagents following the specifications for Megaplex RT Primers and TaqMan® MiRNA Reverse Transcription Kit (Applied Biosystems). 6.0 μL aliquots of each RT reaction were pipetted into 1.7 mL tubes containing 450 μL of TaqMan Universal PCR Master Mix (Applied Biosystems) and 444 μL of RNase-free water, mixed by vortexing, and pipetted into microfluidic TaqMan® Rodent miRNA Array A Low-Density Arrays plates. PCR amplification was performed using an Applied Biosystems 7900HT Fast Real-Time System Thermocycler according to specifications described on MegaPlex^TM^ Pools microRNA Expression Analysis brochure (Applied Biosystems).

### Transfections of antagomirs and mimics using nanoparticles

Nanoparticle forming solutions (NFS) containing antagomirs or mimics (Suppl. Table 5) were prepared following the N-TER^TM^ Peptide protocol (SIGMA) with a few modifications (Suppl. Fig. 1). Briefly, 13.0 μL aliquots of antagomirs or mimics at 5 μM and 37.0 μL of Dilution Buffer were pipetted into 1.7 mL tubes and mixed (Solution 1). 8 μL of N-TER Peptide (SIGMA) and 42 μL of RNase-free water were pipetted into another 1.7 mL tube and mixed (Solution 2). Both solutions 1 and 2 were mixed to form the Nanoparticle Forming Solution (NFS), and incubated for 20 minutes at room temperature prior to transfection into organ explants. Appropriate volumes of NFS solution (Suppl. Fig. 1c) were pipetted into the culture medium of the organ explants prepared as previously described. For transfections of antagomirs and mimics using LFS (Liposome Forming Solution), RNAiFect (QIAGEN) was used following previously described procedures^17^.

### Analysis of early morphogenesis using immunofluorescence

Whole mount organ preparations were used to analyze organs explanted in the floating-filter system (SMGs, lungs, and kidneys), and cryosectioning was used to analyze molar germs in mandible explants, as described (Suppl. Table 6). Quantification of fluorescence was performed using ImageJ Software publically available at http://imagej.nih.gov/ij, according to previously described protocols^27^.

### Analysis of Antagomir-Cy3 uptake by flow cytometry

E13.5 mandibles from a mouse strain constitutively expressing ubiquitous EGFP (also known as Rosa26-Cas9 knockin, JAX 024858) were explanted, and transfected with a Cy3-labeled or unlabeled Off-Target Antagomirs as described above. Explants were cultured for 24 h, and the mandibles treated with Dispase neutral proteases and subjected to epithelial and mesenchymal tissue separation, and to single-cell suspension preparations as previously described. Epithelial and mesenchymal single-cell suspensions from approximately 4–6 mandibular explants were combined and subjected to flow cytometry analysis using an Accuri C6 Flow Cytometer and analyzing the data using the CFlow Plus Software (BD Biosciences).

### Prediction of miRNA target genes

All *in silico* predictions of miRNA target genes were performed using TargetScan (http://www.targetscan.org) and the total number of miRNA targets was filtered using the criteria found in our general prediction pipeline (Suppl. Fig. 6). Briefly, for miR-590–5p, a list containing predicted miRNA targets was generated using TargetScan, and the genes present during tooth morphogenesis were selected using a searchable database for molar tooth germ expression (http://compbio.med.harvard.edu/ToothCODE). The list of selected miRNA targets was subjected to Gene Ontology analysis using the application GeneMania version 3.2.1 found in the downloaded plugin CytoScape (http://www.cytoscape.org)^49^ by applying the following parameters: (i) Predicted Interactions; (ii) Physical Interactions; (iii) Co-Expression; (iv) Co-Localization; (v) Gene Interactions; and (vi). Pathways, Weighting window: GO Biolgical Process-Based. The miR590-5p targets were scored and ranked according to Gene Ontology Identifications (GO IDs) and the corresponding q-values (Suppl. Table 2, Suppl. Fig. 6). A selected group of miR-590-5p targets in the first two GO entries: *Chd7*, *Pitx2a*, *Msx1*, and *Bcl11b*, were selected for further qRT-PCR analysis, and the putative non-targets *Shh*, *Bmp4*, and *Sox2*, were also included.

### Analysis of miRNA expression by qRT-PCR

All Reverse Transcription reactions to analyze expression of individual miRNAs were performed using 200 ng of total RNA and reagents and specifications in the miScript II RT kit (QIAGEN). Detection of mature miRNA expression was performed using reagents and specifications found in the miScript SYBR PCR kit (QIAGEN), and the corresponding PCR primers (Suppl. Table 7).

### Analysis of mRNA expression by qRT-PCR

Reverse Transcription (RT) reactions were prepared using 200 ng of total RNA and reagents and specifications in the iScript cDNA Synthesis kit (BIORAD). Dilutions containing 1 ng of cDNA from the RT reactions were used to amplify the PCR products using iQ™ SYBR® Green Supermix (BIORAD). PCR reactions were performed in a Thermal Cycler CFX96 C100 (BIORAD) using the corresponding PCR primer sequences (Suppl. Table 7). Calculations of fold change in expression were performed as previously described^28^.

### miRNA pulldown (miR-PD)

The miR-PD was performed using an adapted protocol^42^ and following the specifications in the Dynabeads® MyOne™ Streptavidin T1 Reagent (Invitrogen). Briefly, a 100 μL aliquot of magnetic streptavidin beads was pipetted into a 1.7 mL tube, and placed on ice. The beads were washed (100 μL of miR-PD Lysis solution), and blocked (200 μL of miR-PD Blocking Solution, 2 h incubation, 4 °C, rocker). Explanted organs were collected into 1.7 mL tubes, lysed with 200 μL of miR-PD Lysis Buffer, and homogenized (10 seconds, on ice). The tubes were sealed with Parafilm and incubated (5 minutes, ice), centrifuged (10,000 rpm, 10 minutes), and the supernatant (Input Fraction) collected into a new 1.7 mL sterile tube. The blocking solution was removed from the beads using a MagnaRack for Microcentrifuge Tubes (Invitrogen), and the Input fractions were transferred into the tubes containing the beads and incubated (4 °C, 4 h). The tubes were placed in the MagnaRack, the supernatant (Input – PD fraction) was pipetted into a new 1.7 mL tube, and the remaining beads (PD fraction) were washed 5 times with ice-cold miR-PD Lysis Solution. RNA extraction was performed using a 50.0 μL aliquot of the Input-PD and the PD fractions, and gene expression assessed by qRT-PCR analysis.

## Additional Information

**How to cite this article**: Rebustini, I. T. *et al.* An integrated miRNA functional screening and target validation method for organ morphogenesis. *Sci. Rep.* **6**, 23215; doi: 10.1038/srep23215 (2016).

## Supplementary Material

Supplementary Information

## Figures and Tables

**Figure 1:**
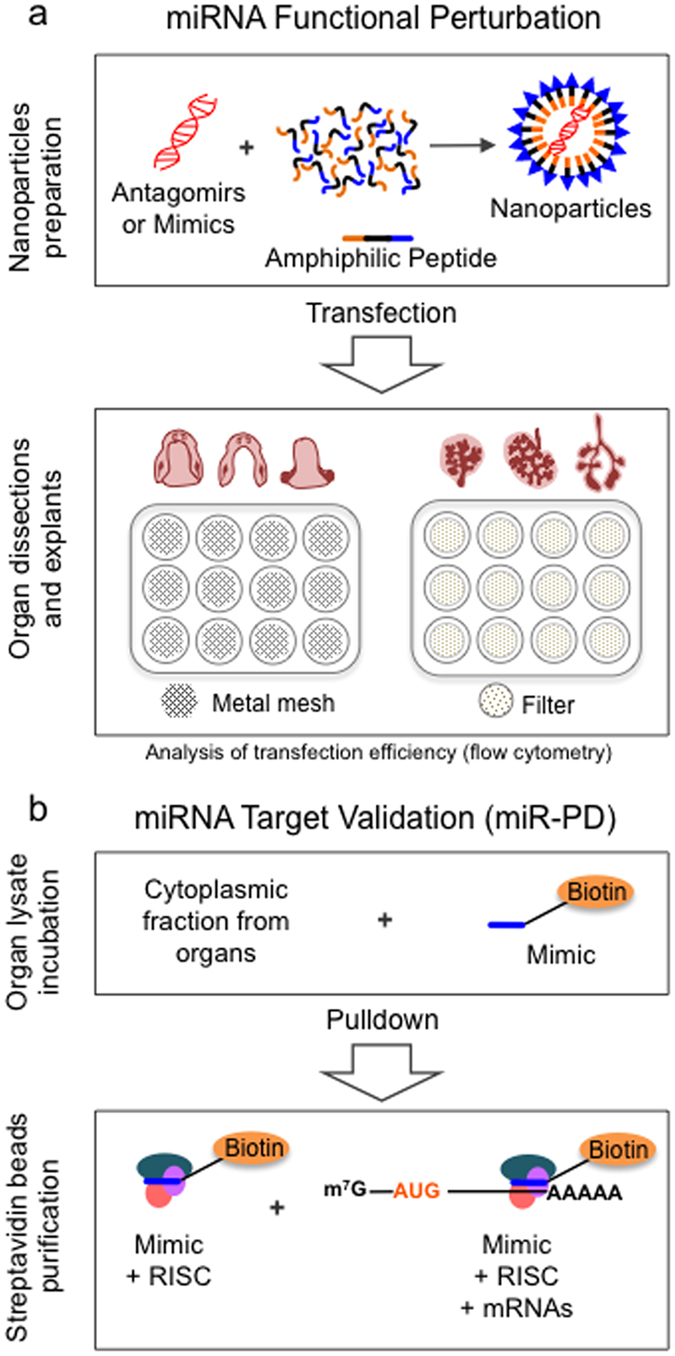
Integrated miRNA functional screening and target validation method. The method consists of two parts: **(a)** miRNA Functional Perturbation, and **(b)** miRNA Target Validation (miR-PD). **(a)** The miRNA Functional Perturbation involves preparation of Nanoparticles by loading an amphiphilic peptide with miRNA antagomir (inhibitor) or mimic (activator) to functionally target a miRNA of interest. Alternatively, Liposome transfection may be used (see Methods). Murine embryonic organs of interest are micro-dissected and explanted into multi-well culture plates, either directly onto metal mesh in a modified Trowel-type system (*e.g.,* intact manbibles, isolated mandibles, tongue) or directly onto floating Nuclepore filters (*e.g.,* using salivary glands, kidneys, lungs). This system provides moderate analytical throughput by allowing use of different experimental conditions and phenotypic assays in parallel. **(b)** The miRNA Target Validation (miR-PD; miRNA-Pull Down Assay) involves preparations of organ lysates and incubation of the cytoplasmic fraction with biotinylated-mimic miRNA (mimic-biotin), followed by a streptavidin bead purification step to recover the biotinylated-miRNA mimic and its target mRNA transcripts. RISC: RNA Interference Silencing Complex (necessary for miRNA:mRNA interactions).

## References

[b1] Landgraf P. *et al.* A Mammalian microRNA Expression Atlas Based on Small RNA Library Sequencing. Cell 129, 1401–1414 (2007). 17604727 10.1016/j.cell.2007.04.040 PMC2681231

[b2] Krützfeldt J. *et al.* Silencing of microRNAs *in vivo* with ‘antagomirs’. Nature 438, 685–689 (2005). 16258535 10.1038/nature04303

[b3] Xie J. *et al.* Long-term, efficient inhibition of microRNA function in mice using rAAV vectors. Nature Publishing Group 9, 403–409 (2012). 10.1038/nmeth.1903 PMC3420816 22388288

[b4] Park C. Y., Choi Y. S. & McManus M. T. Analysis of microRNA knockouts in mice. Human Molecular Genetics 19, R169–R175 (2010). 20805106 10.1093/hmg/ddq367 PMC2981466

[b5] Rebustini I. T. *et al.* MT2-MMP-dependent release of collagen IV NC1 domains regulates submandibular gland branching morphogenesis. Developmental Cell 17, 482–493 (2009). 19853562 10.1016/j.devcel.2009.07.016 PMC2768621

[b6] del Moral P.-M. & Warburton D. Explant culture of mouse embryonic whole lung, isolated epithelium, or mesenchyme under chemically defined conditions as a system to evaluate the molecular mechanism of branching morphogenesis and cellular differentiation. Methods Mol. Biol. 633, 71–79 (2010). 20204620 10.1007/978-1-59745-019-5_5 PMC3120103

[b7] Costantini F., Watanabe T., Lu B., Chi X. & Srinivas S. Imaging kidney development. Cold Spring Harb Protoc 2011, pdb.top109 (2011). 10.1101/pdb.top109 21536773

[b8] Munne P. M., Närhi K. & Michon F. In Methods in Molecular Biology 945, 401–416 (Humana Press, 2012). 23097120 10.1007/978-1-62703-125-7_24

[b9] Kavanagh K. D., Evans A. R. & Jernvall J. Predicting evolutionary patterns of mammalian teeth from development. Nature 449, 427–432 (2007). 17898761 10.1038/nature06153

[b10] Shamir E. R. & Ewald A. J. Three-dimensional organotypic culture: experimental models of mammalian biology and disease. Nat. Rev. Mol. Cell Biol. 15, 647–664 (2014). 25237826 10.1038/nrm3873 PMC4352326

[b11] Guo H., Ingolia N. T., Weissman J. S. & Bartel D. P. Mammalian microRNAs predominantly act to decrease target mRNA levels. Nature 466, 835–840 (2010). 20703300 10.1038/nature09267 PMC2990499

[b12] Zamore P. D. & Haley B. Ribo-gnome: the big world of small RNAs. Science 309, 1519–1524 (2005). 16141061 10.1126/science.1111444

[b13] Yin H. *et al.* Non-viral vectors for gene-based therapy. Nat. Rev. Genet. 15, 541–555 (2014). 25022906 10.1038/nrg3763

[b14] de Fougerolles A., Vornlocher H.-P., Maraganore J. & Lieberman J. Interfering with disease: a progress report on siRNA-based therapeutics. Nat Rev Drug Discov 6, 443–453 (2007). 17541417 10.1038/nrd2310 PMC7098199

[b15] Roberts P., Noerholm M., Ståhlberg N., Mouritzen P. & Glue C. miRCURY™ LNA research tools for microRNA. Nature Publishing Group 3 (2006).

[b16] Obernosterer G., Martinez J. & Alenius M. Locked nucleic acid-based *in situ* detection of microRNAs in mouse tissue sections. Nat Protoc 2, 1508–1514 (2007). 17571058 10.1038/nprot.2007.153

[b17] Rebustini I. T. *et al.* miR-200c regulates FGFR-dependent epithelial proliferation via Vldlr during submandibular gland branching morphogenesis. Development 139, 191–202 (2012). 22115756 10.1242/dev.070151 PMC3231777

[b18] Simeoni F., Morris M. C., Heitz F. & Divita G. Insight into the mechanism of the peptide-based gene delivery system MPG: implications for delivery of siRNA into mammalian cells. Nucleic Acids Res. 31, 2717–2724 (2003). 12771197 10.1093/nar/gkg385 PMC156720

[b19] Crombez L. *et al.* A non-covalent peptide-based strategy for siRNA delivery. Biochem. Soc. Trans. 35, 44–46 (2007). 17233597 10.1042/BST0350044

[b20] Liu X. S. *et al.* MicroRNA Profiling in Subventricular Zone after Stroke: MiR-124a Regulates Proliferation of Neural Progenitor Cells through Notch Signaling Pathway. PLoS ONE 6, e23461 (2011). 21887253 10.1371/journal.pone.0023461 PMC3162555

[b21] Colas A. R. *et al.* Whole-genome microRNA screening identifies let-7 and mir-18 as regulators of germ layer formation during early embryogenesis. Genes & Development 26, 2567–2579 (2012). 23152446 10.1101/gad.200758.112 PMC3521625

[b22] Thiagarajan R. D. *et al.* Refining transcriptional programs in kidney development by integration of deep RNA-sequencing and array-based spatial profiling. BMC Genomics 12, 441 (2011). 21888672 10.1186/1471-2164-12-441 PMC3180702

[b23] Narayan A., Bommakanti A. & Patel A. A. High-throughput RNA profiling via up-front sample parallelization. Nature Publishing Group, 10.1038/nmeth.3311 (2015). PMC4451056 25730493

[b24] Wang B. *et al.* Systematic evaluation of three microRNA profiling platforms: microarray, beads array, and quantitative real-time PCR array. PLoS ONE 6, e17167 (2011). 21347261 10.1371/journal.pone.0017167 PMC3037970

[b25] Michon F., Tummers M., Kyyrönen M., Frilander M. J. & Thesleff I. Tooth morphogenesis and ameloblast differentiation are regulated by micro-RNAs. Developmental Biology 340, 355–368 (2010). 20102707 10.1016/j.ydbio.2010.01.019

[b26] Simpson L. J. *et al.* A microRNA upregulated in asthma airway T cells promotes TH2 cytokine production. Nat. Immunol. 15, 1162–1170 (2014). 25362490 10.1038/ni.3026 PMC4233009

[b27] Rebustini I. T. & Hoffman M. P. ECM and FGF-dependent assay of embryonic SMG epithelial morphogenesis: investigating growth factor/matrix regulation of gene expression during submandibular gland development. Methods Mol. Biol. 522, 319–330 (2009). 19247608 10.1007/978-1-59745-413-1_21 PMC3375330

[b28] Schmittgen T. D. & Livak K. J. Analyzing real-time PCR data by the comparative C(T) method. Nat Protoc 3, 1101–1108 (2008). 18546601 10.1038/nprot.2008.73

[b29] Cao H. *et al.* MicroRNAs Play a Critical Role in Tooth Development. Journal of Dental Research 89, 779–784 (2010). 20505045 10.1177/0022034510369304 PMC3014323

[b30] Ren J. *et al.* Signature of circulating microRNAs as potential biomarkers in vulnerable coronary artery disease. PLoS ONE 8, e80738 (2013). 24339880 10.1371/journal.pone.0080738 PMC3855151

[b31] Cao H. *et al.* The Pitx2:miR-200c/141:noggin pathway regulates Bmp signaling and ameloblast differentiation. Development 140, 3348–3359 (2013). 23863486 10.1242/dev.089193 PMC3737717

[b32] Ahmed M. I., Mardaryev A. N., Lewis C. J., Sharov A. A. & Botchkareva N. V. MicroRNA-21 is an important downstream component of BMP signalling in epidermal keratinocytes. J. Cell. Sci. 124, 3399–3404 (2011). 21984808 10.1242/jcs.086710 PMC3196856

[b33] Zhou X. *et al.* Reduction of miR-21 induces glioma cell apoptosis via activating caspase 9 and 3. Oncol. Rep. 24, 195–201 (2010). 20514462 10.3892/or_00000846

[b34] Grimson A. *et al.* MicroRNA targeting specificity in mammals: determinants beyond seed pairing. Mol. Cell 27, 91–105 (2007). 17612493 10.1016/j.molcel.2007.06.017 PMC3800283

[b35] O’Connell D. J. *et al.* A Wnt-bmp feedback circuit controls intertissue signaling dynamics in tooth organogenesis. Sci Signal 5, ra4 (2012). 22234613 10.1126/scisignal.2002414 PMC10292157

[b36] Warde-Farley D. *et al.* The GeneMANIA prediction server: biological network integration for gene prioritization and predicting gene function. Nucleic Acids Res. 38, W214–20 (2010). 20576703 10.1093/nar/gkq537 PMC2896186

[b37] Costantini F., Watanabe T., Lu B., Chi X. & Srinivas S. Dissection of embryonic mouse kidney, culture *in vitro*, and imaging of the developing organ. Cold Spring Harb Protoc 2011, pdb.prot5613 (2011). 10.1101/pdb.prot5613 21536763

[b38] Obad S. *et al.* Silencing of microRNA families by seed-targeting tiny LNAs. Nat Genet 43, 371–378 (2011). 21423181 10.1038/ng.786 PMC3541685

[b39] Fluiter K., Mook O. R. F. & Baas F. The therapeutic potential of LNA-modified siRNAs: reduction of off-target effects by chemical modification of the siRNA sequence. Methods Mol. Biol. 487, 189–203 (2009). 19301648 10.1007/978-1-60327-547-7_9

[b40] Chen Y., Bei M., Woo I., Satokata I. & Maas R. Msx1 controls inductive signaling in mammalian tooth morphogenesis. Development 122, 3035–3044 (1996). 8898217 10.1242/dev.122.10.3035

[b41] Engelen E. *et al.* Sox2 cooperates with Chd7 to regulate genes that are mutated in human syndromes. Nat Genet 43, 607–611 (2011). 21532573 10.1038/ng.825

[b42] Lal A. *et al.* Capture of microRNA-bound mRNAs identifies the tumor suppressor miR-34a as a regulator of growth factor signaling. PLoS Genet 7, e1002363 (2011). 22102825 10.1371/journal.pgen.1002363 PMC3213160

[b43] Nicolas F. E. Experimental validation of microRNA targets using a luciferase reporter system. Methods Mol. Biol. 732, 139–152 (2011). 21431711 10.1007/978-1-61779-083-6_11

[b44] Moore M. J. *et al.* Mapping Argonaute and conventional RNA-binding protein interactions with RNA at single-nucleotide resolution using HITS-CLIP and CIMS analysis. Nat Protoc 9, 263–293 (2014). 24407355 10.1038/nprot.2014.012 PMC4156013

[b45] Hamzeiy H., Allmer J. & Yousef M. Computational methods for microRNA target prediction. Methods Mol. Biol. 1107, 207–221 (2014). 24272439 10.1007/978-1-62703-748-8_12

[b46] Mali P., Esvelt K. M. & Church G. M. Cas9 as a versatile tool for engineering biology. Nature Publishing Group 10, 957–963 (2013). 10.1038/nmeth.2649 PMC4051438 24076990

[b47] Sommer D., Peters A. E., Baumgart A.-K. & Beyer M. TALEN-mediated genome engineering to generate targeted mice. Chromosome Res. 10.1007/s10577-014-9457-1 (2015). 25596827

[b48] Närhi K. & Thesleff I. Explant culture of embryonic craniofacial tissues: analyzing effects of signaling molecules on gene expression. Methods Mol. Biol. 666, 253–267 (2010). 20717789 10.1007/978-1-60761-820-1_16

[b49] Montojo J. *et al.* GeneMANIA Cytoscape plugin: fast gene function predictions on the desktop. Bioinformatics 26, 2927–2928 (2010). 20926419 10.1093/bioinformatics/btq562 PMC2971582

