# Supplementary material for: An integrated miRNA functional screening and target validation method for organ morphogenesis
Source: Sci Rep. 2016 Mar 16;6:23215. doi: 10.1038/srep23215 (PMC4793243; doi:10.1038/srep23215)
Supplement: Supplementary Information [file srep23215-s1.pdf]

Supplementary Information

An integrated miRNA functional screening and target validation method for organ morphogenesis

[1] Ivan T. Rebustini, [1] Maryann Vlahos, [2] Trevor Packer, [2] Maria A. Kukuruzinska, and [1] Richard L. Maas*

[1] Division of Genetics, Department of Medicine, Brigham and Women's Hospital, Harvard Medical School, Boston, MA 02115, USA

[2] Department of Molecular and Cell Biology, Boston University Medical Center, Boston, MA 02118, USA

Correspondence should be addressed to maas@genetics.med.harvard.edu

Supplementary Figure 1

a

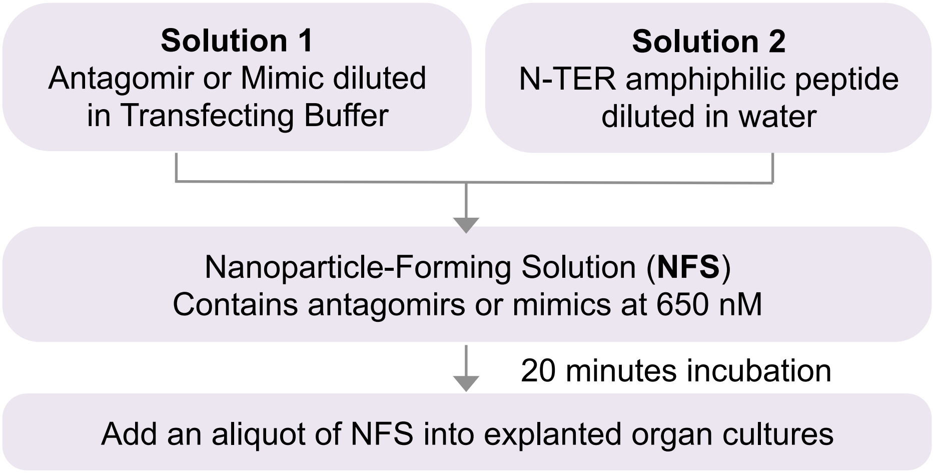

b

**Ratio of N-TER peptide : Antagomir or Mimic**	**Volume (uL) of stock Antagomir or Mimic + Transfection Buffer (Solution 1)**	**Volume (µL) of N-TER peptide + H_2O (Solution 2)**	**Concentration (in nM) of Antagomir or Mimic (NFS solution)**
2 : 1	32.5 + 17.5	8.0 + 42.0	1625
5 : 1	13.0 + 37.0	8.0 + 42.0	650
20 : 1	3.3 + 46.5	8.0 + 42.0	162.5

c

**Aliquots (µL) of NFS at 650 nM**	**Final Antagomir or Mimic concentration (nM)**
31	10
155	50
310	100

Supplementary Figure 2

a

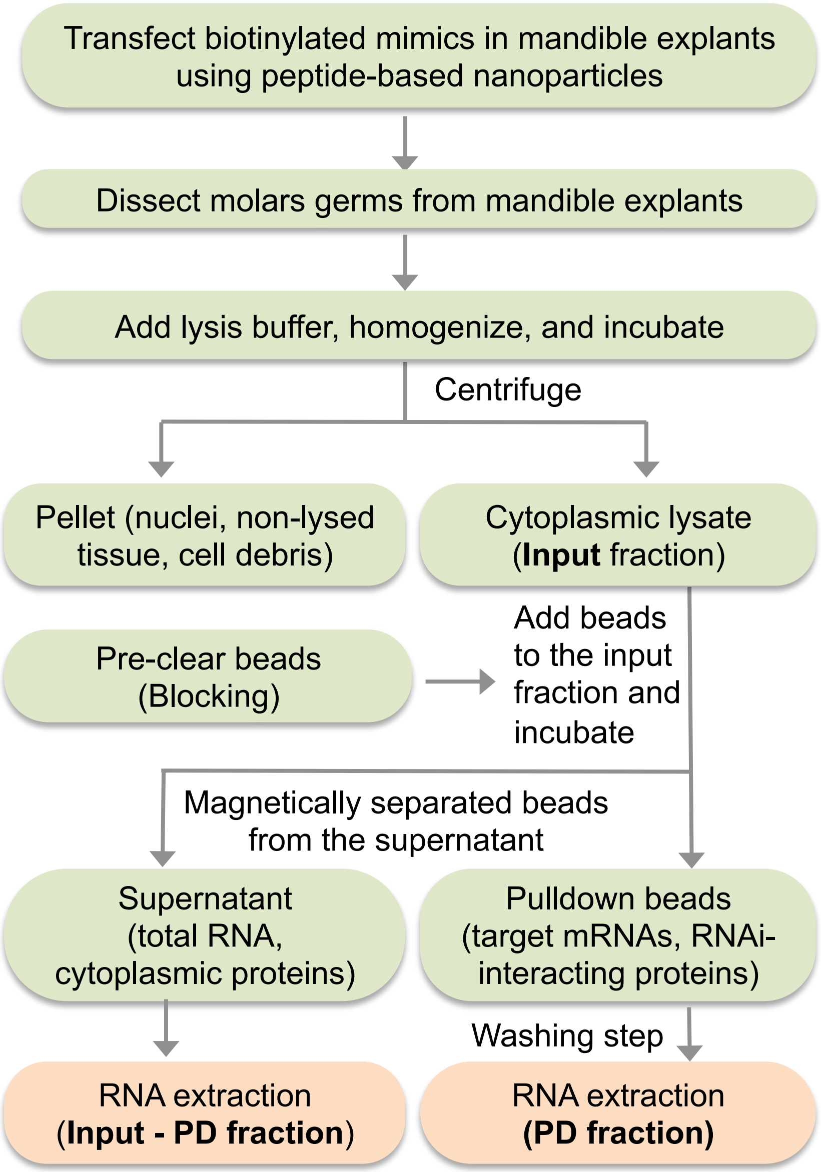

b

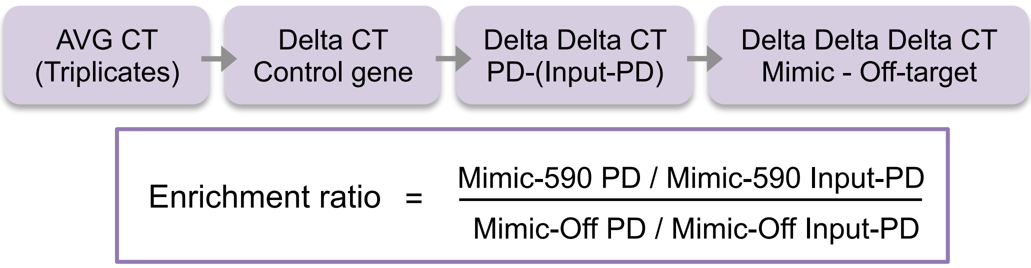

Supplementary Figure 3

a

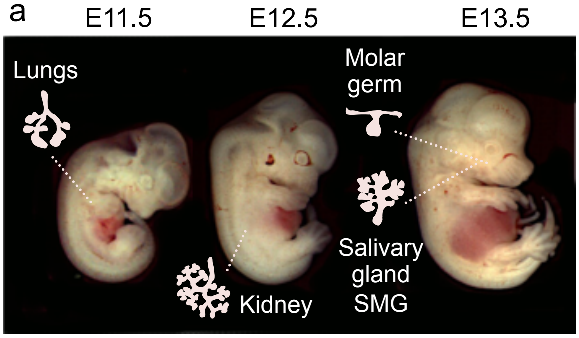

	Number of organs	RNA Concentration (ng/µL)	Amount of RNA recovered in 50 µL (ng)	RNA/organ (ng)
E11.5 Lungs	28	416.2	20810	743.2
E12.5 Kidneys	30	169.8	8490	283.0
E13.5 Molars	22	70.7	3535	160.7
E13.5 SMGs	22	129.7	6485	294.8

b

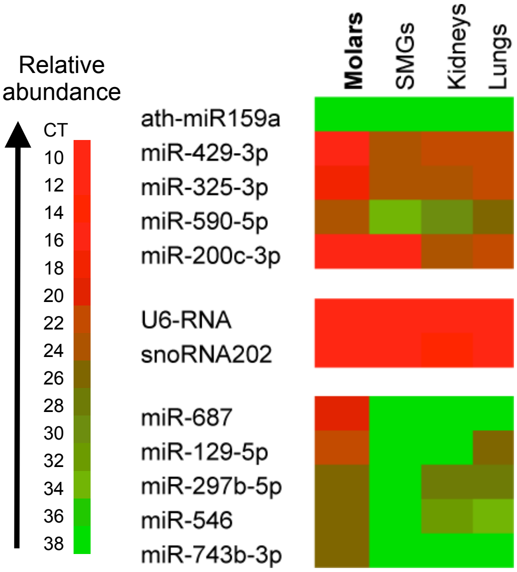

c

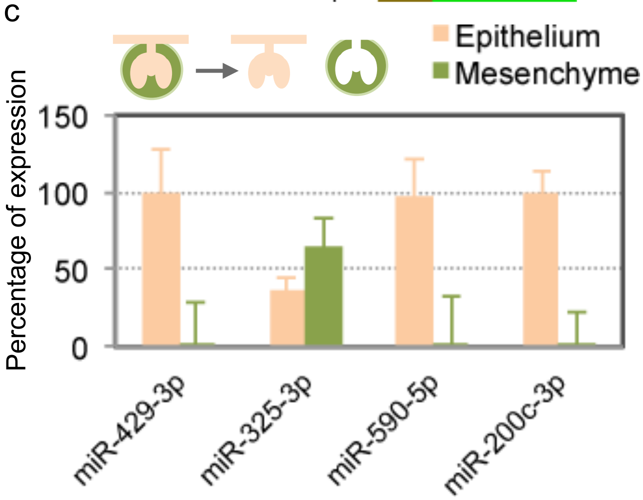

Supplementary Figure 4

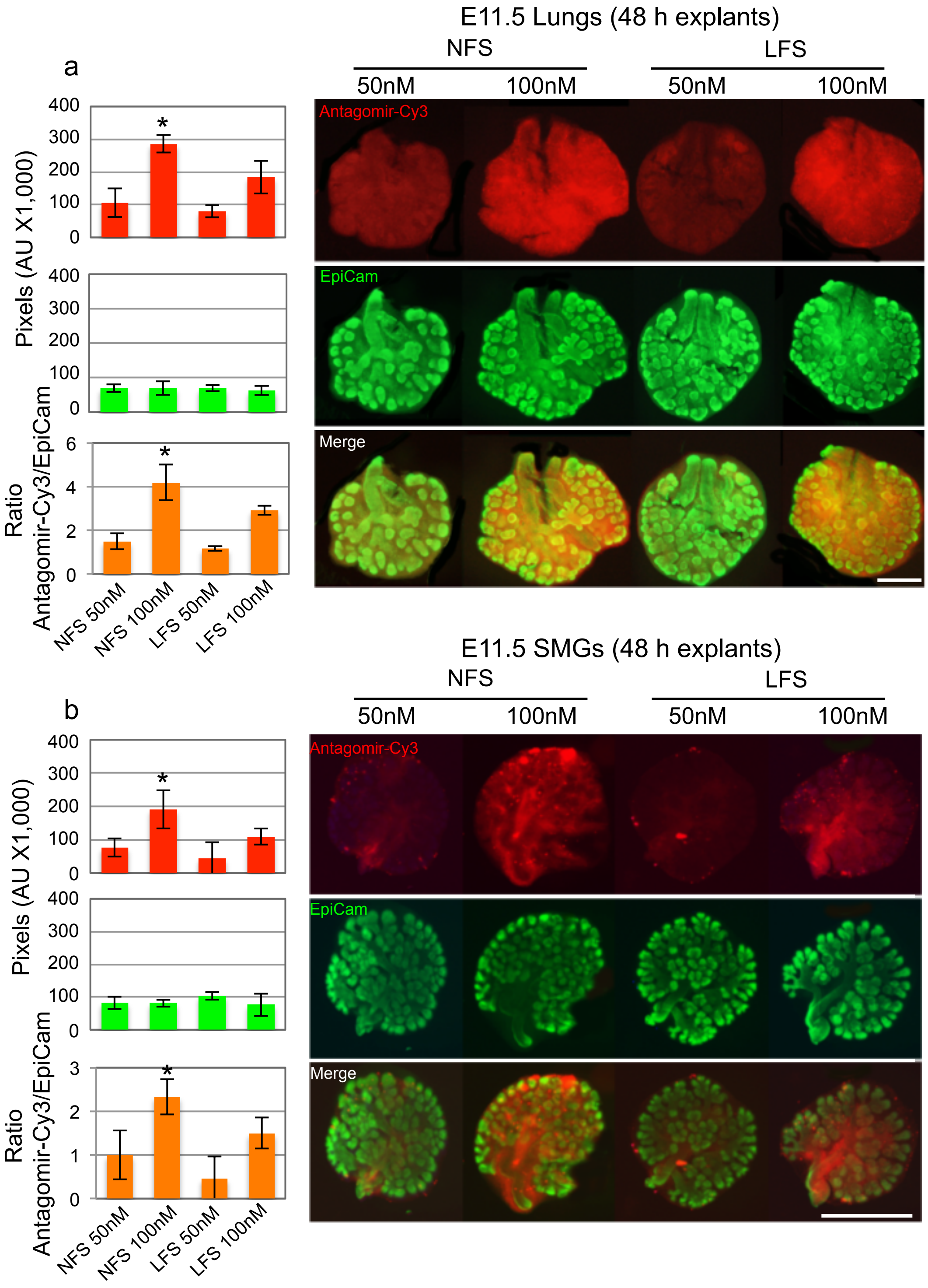

Supplementary Figure 4

c

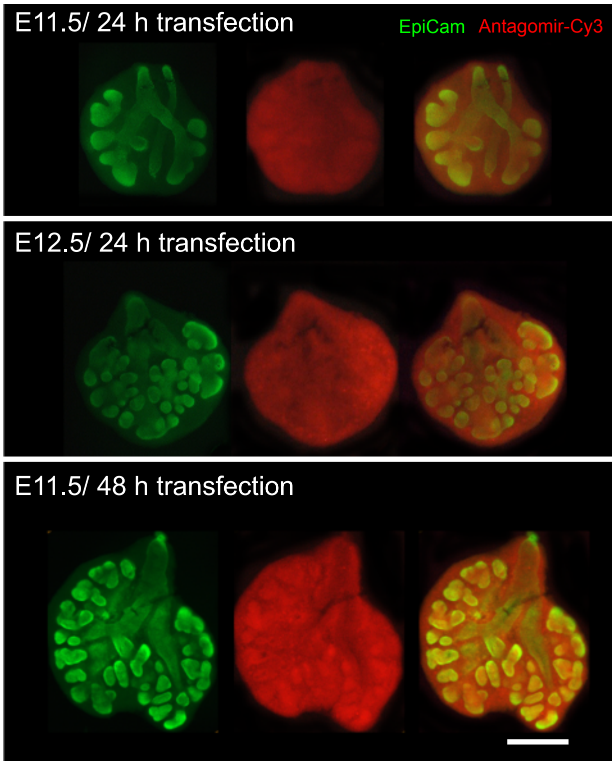

d

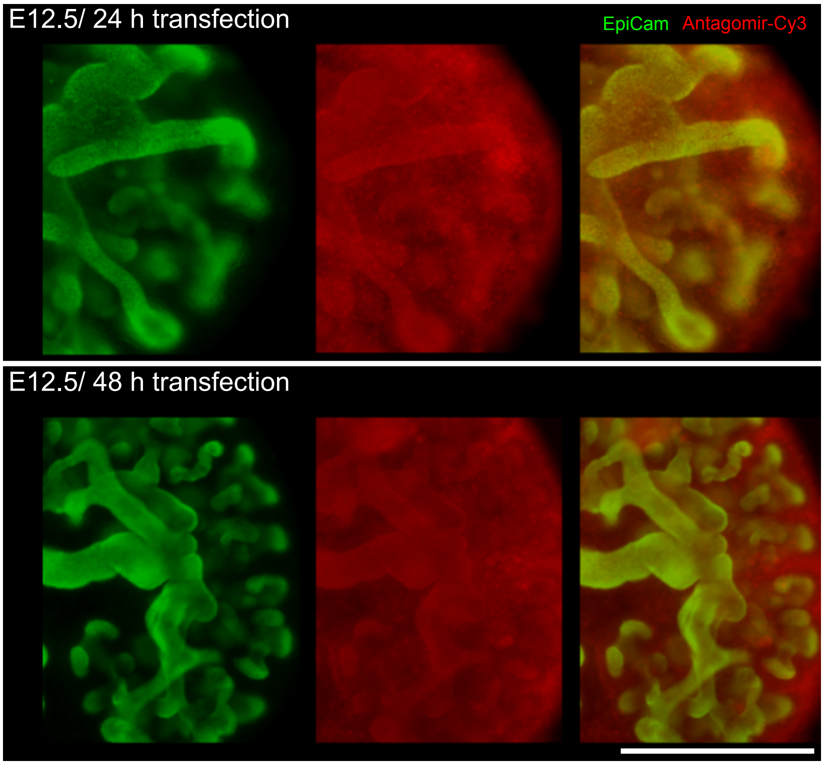

Supplementary Figure 5

a

Egfp-expressing E13.5 mandibular explants → Nanoparticle transfection of Off-Target Antagomirs → Epithelial and mesenchymal separation using Dispase II → Epithelial and mesenchymal single cell suspensions → Flow cytometry analysis of Cy3-miRNA uptake

Experimental groups:

1. Cy3-labeled Off-Target Antagomir
2. Unlabeled Off-Target Antagomir
3. Untreated explants

b

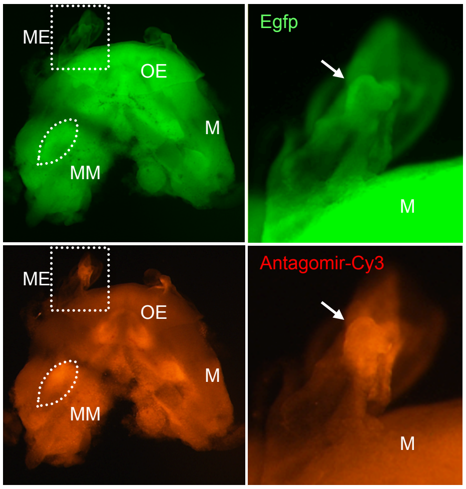

c

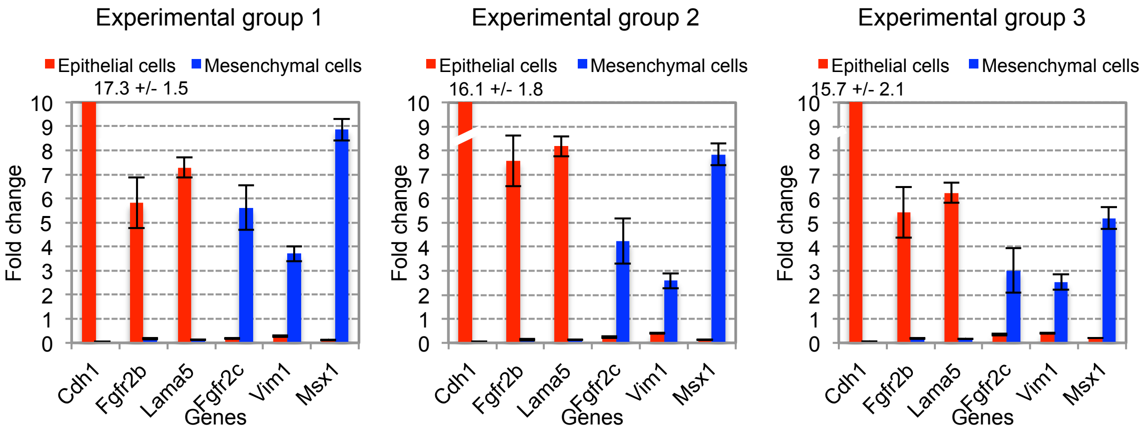

Supplementary Figure 5

d

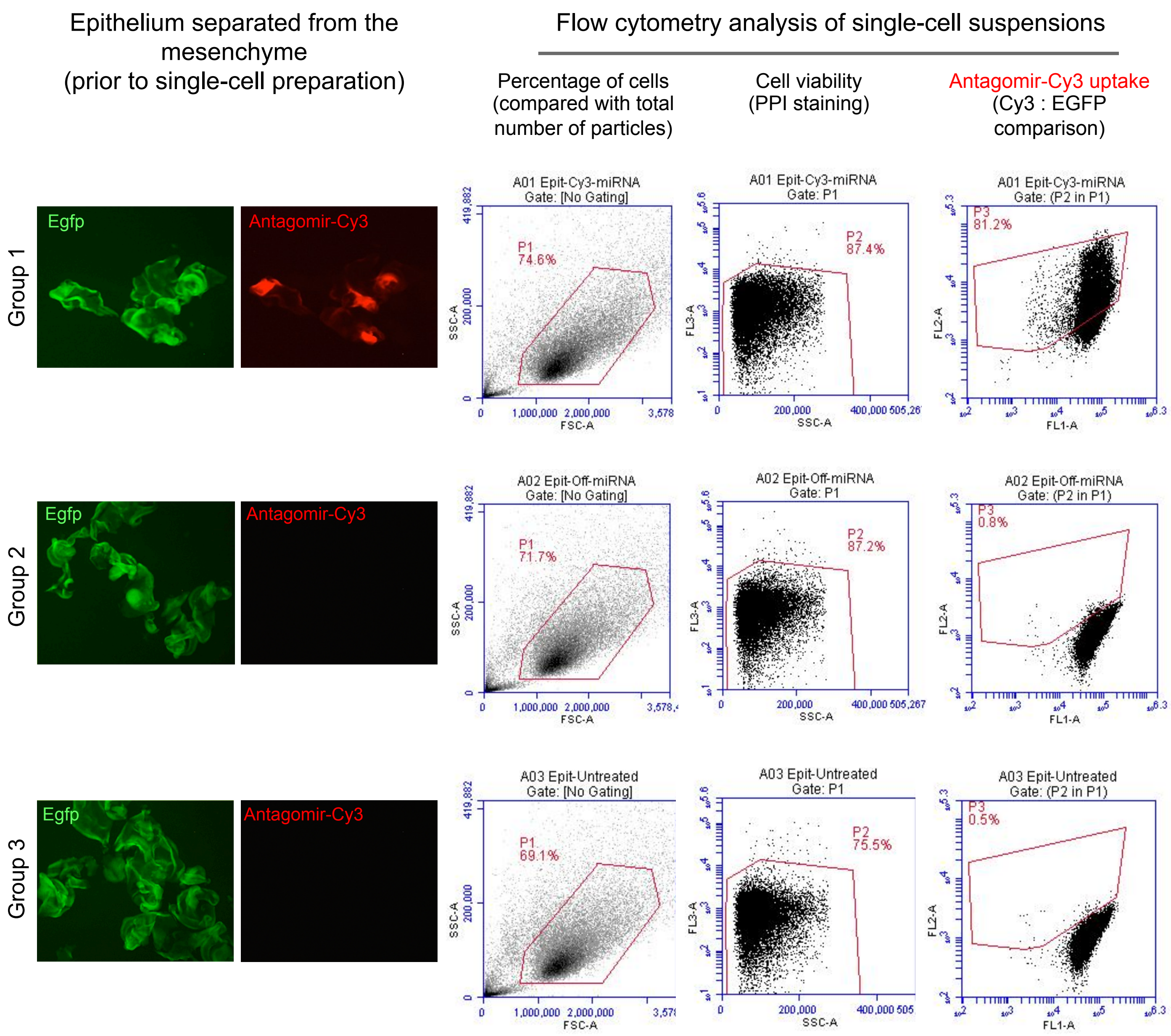

Experimental groups:

1. Cy3-labeled Off-Target Antagomir
2. Unlabeled Off-Target Antagomir
3. Untreated explants

The ability to detect dead cells is important in determining the effectiveness of cytotoxic agents in cell or tissue culture protocols. Propidium iodide (PPI) exclusion, which is analogous to the non-fluorescent trypan blue dye test for cell viability, was used to detect cell viability(and intact nuclei) after organ culture conditions and transfections).

Supplementary Figure 5

e

Mesenchyme separated from the epithelium
(prior to single-cell preparation)

Flow cytometry analysis of single-cell suspensions

Percentage of cells (compared with total number of particles)

Cell viability (PPI staining)

Antagomir-Cy3 uptake (Cy3 : EGFP comparison)

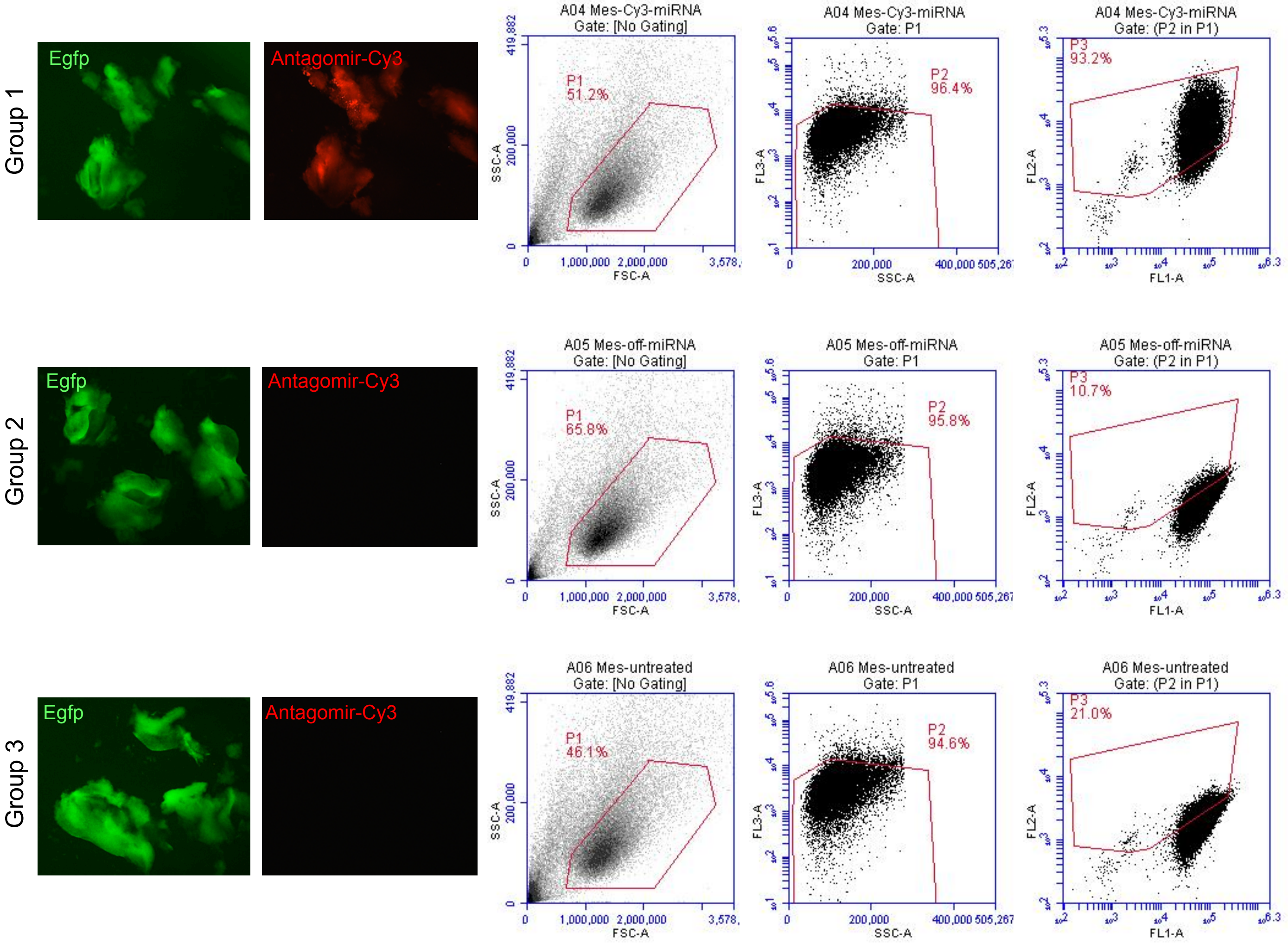

Experimental groups:

1. Cy3-labeled Off-Target Antagomir
2. Unlabeled Off-Target Antagomir
3. Untreated explants

The ability to detect dead cells is important in determining the effectiveness of cytotoxic agents in cell or tissue culture protocols. Propidium iodide (PPI) exclusion, which is analogous to the non-fluorescent trypan blue dye test for cell viability, was used to detect cell viability(and intact nuclei) after organ culture conditions and transfections).

Supplementary Figure 6

a

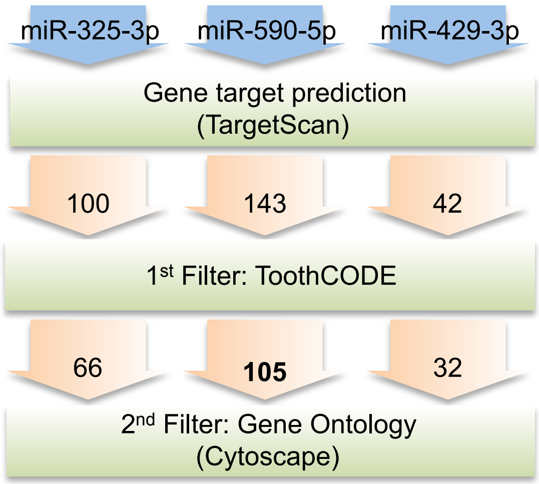

b

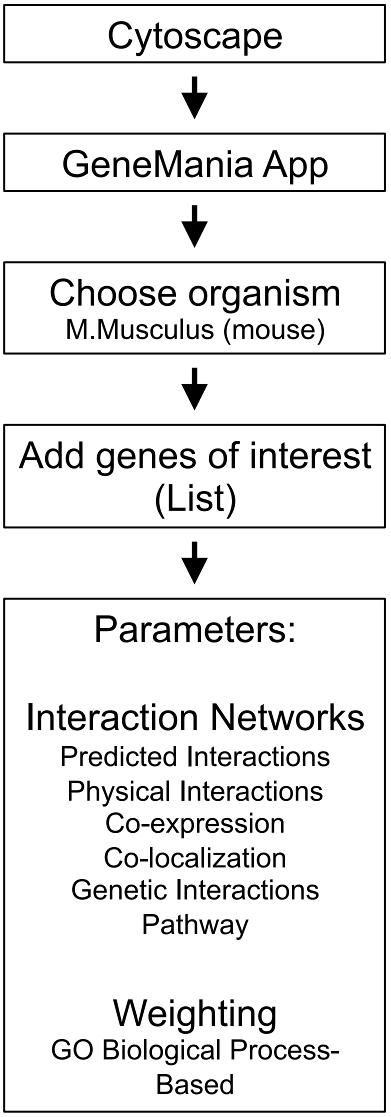

c

GO ID	Description	q-value
GO:0060021	Palate Development	0.002182048
GO:0042476	Odontogenesis	0.002200709

d

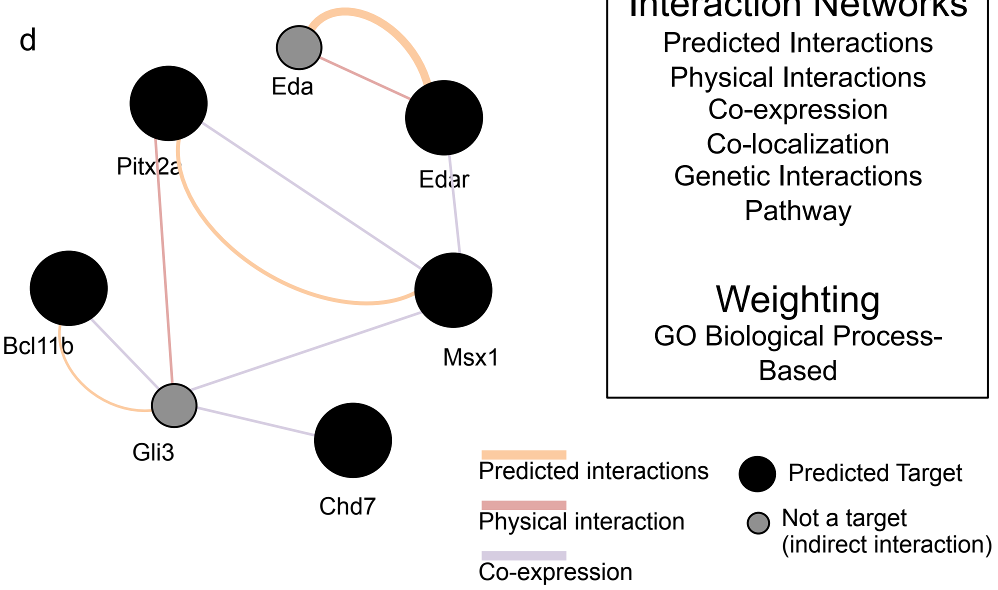

Supplementary Figure 7

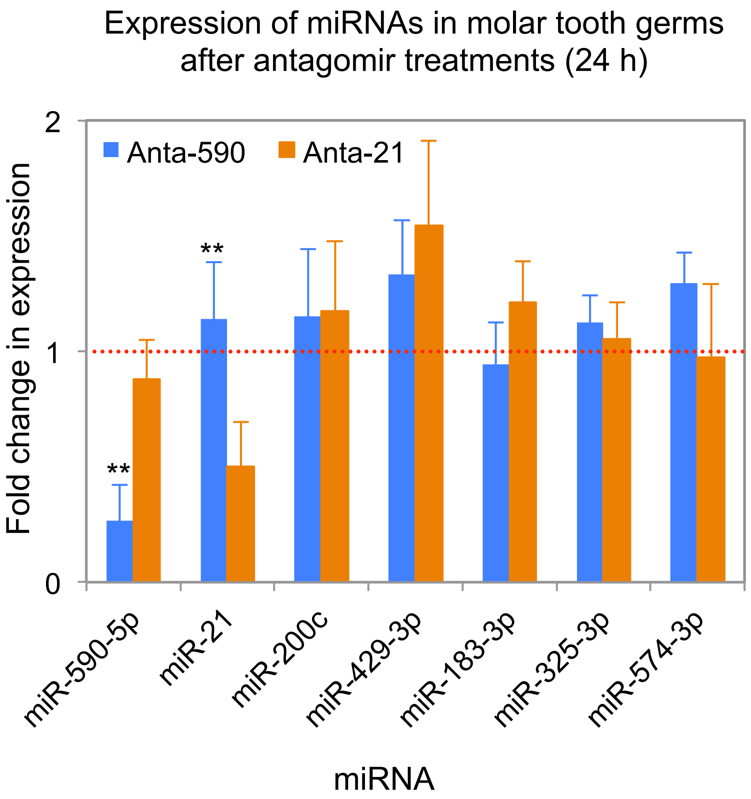

Student's T-Test:
Anta-590 versus Anta-21

miRNA	P value	Significance
miR-590-5p	0.01	**
miR-21	0.01	**
miR-200c	0.84	NS
miR-429-3p	0.23	NS
miR-183-3p	0.15	NS
miR-325-3p	0.26	NS
miR-574-3p	0.07	NS

Significance by default	
P > 0.05	NS
P < 0.05	*
P < 0.01	**
P < 0.001	***
P < 0.0001	****

Supplementary Figure 8

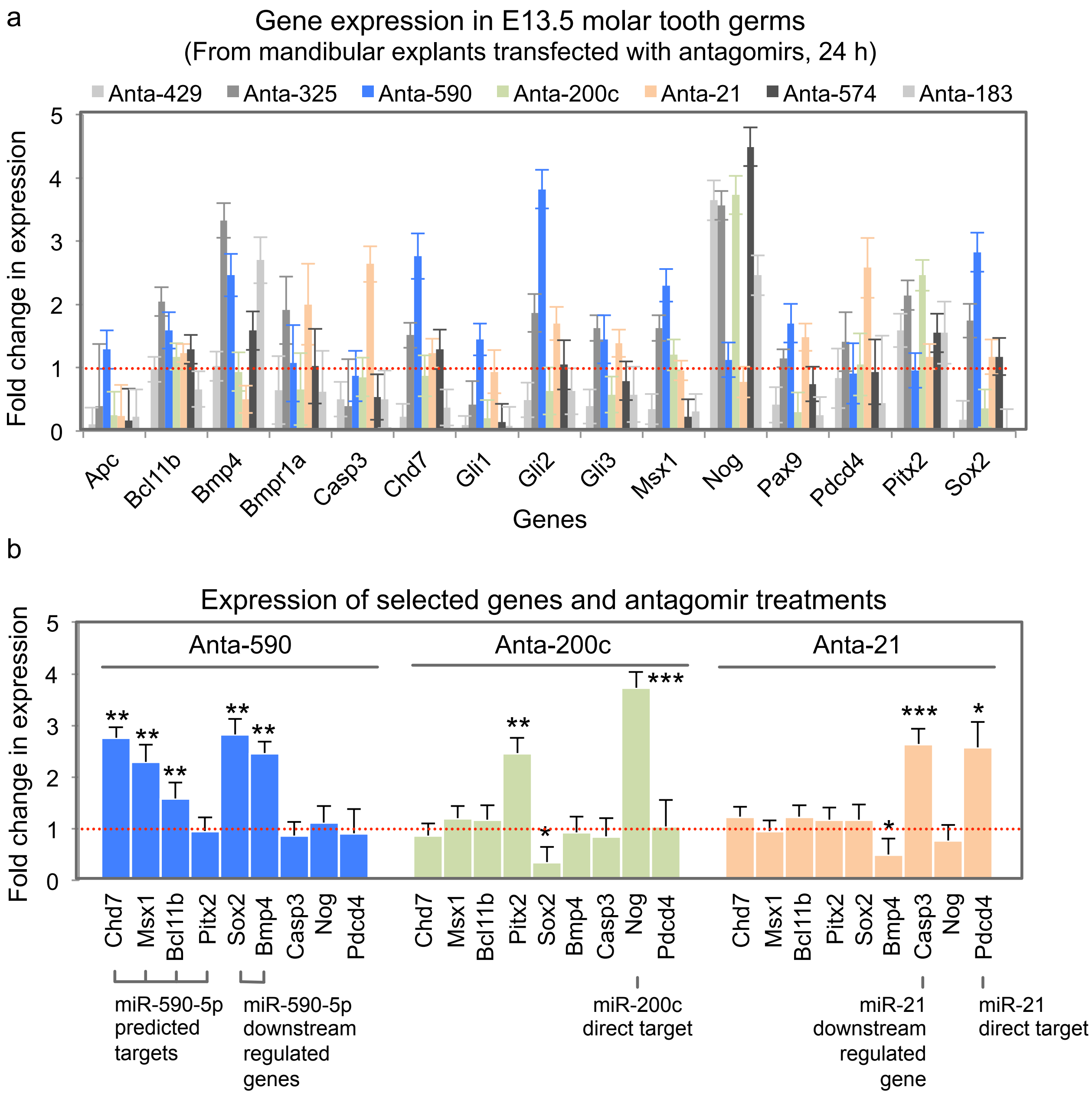

Supplementary Figure 9

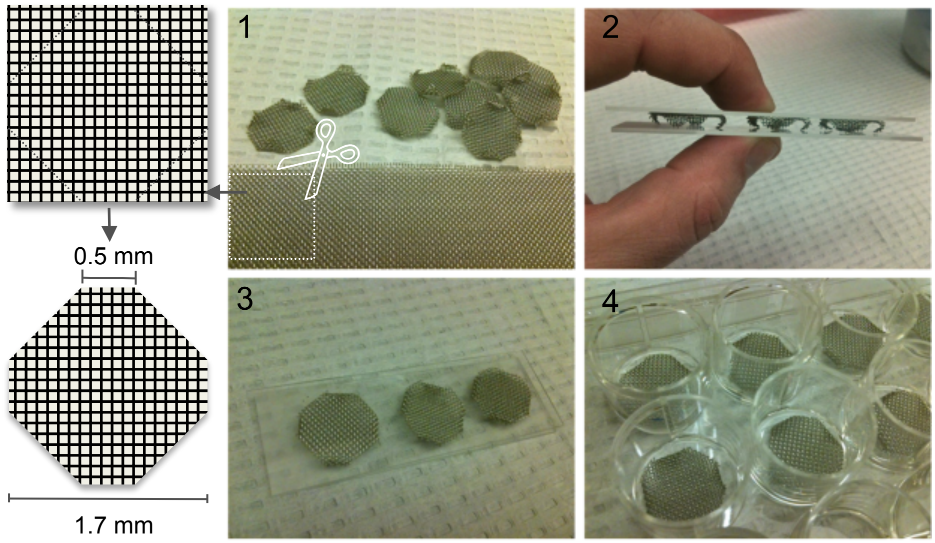

Supplementary Figure 10

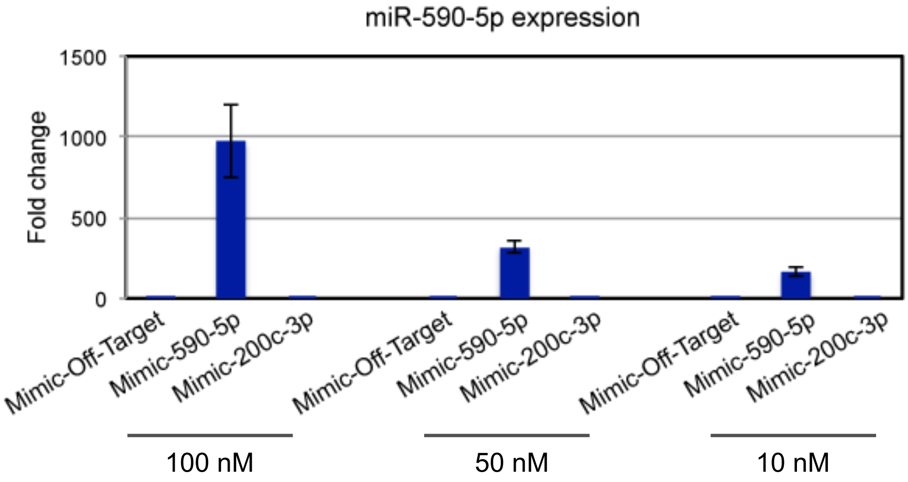

Supplementary Figure 11

E11.5 mandibular coronal frozen section

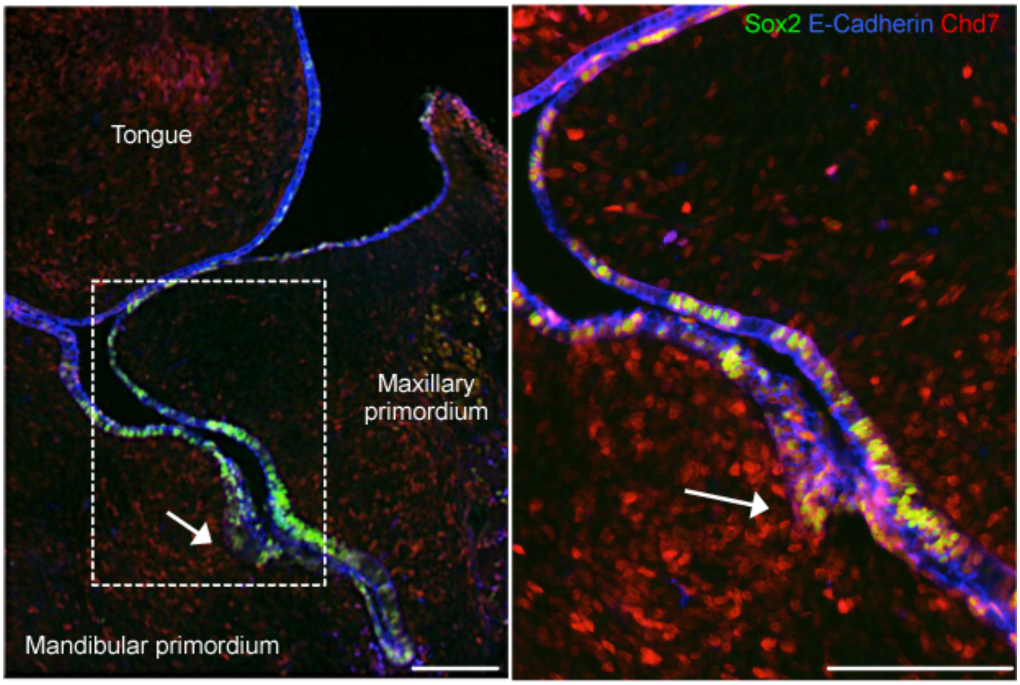

Arrow: oral epithelium placode (molar tooth germ)

Supplementary Figure 12

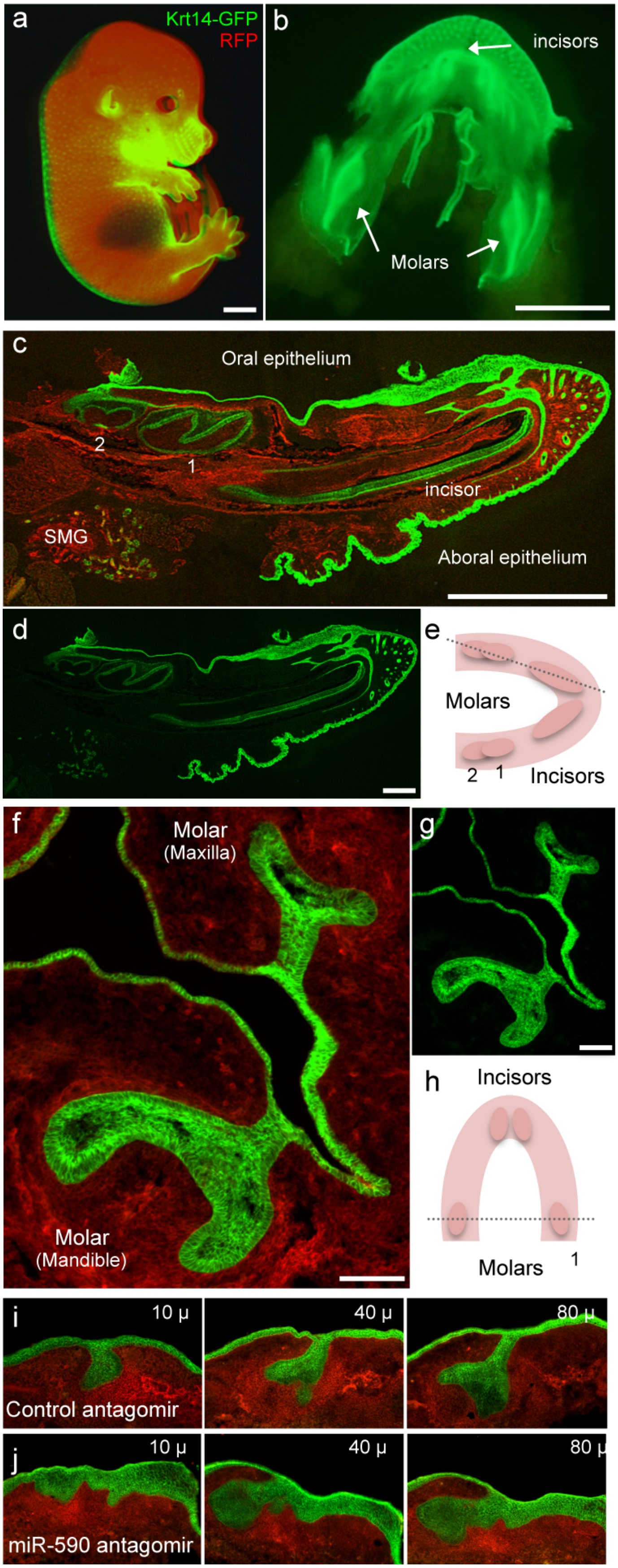

Supplementary Figure 13

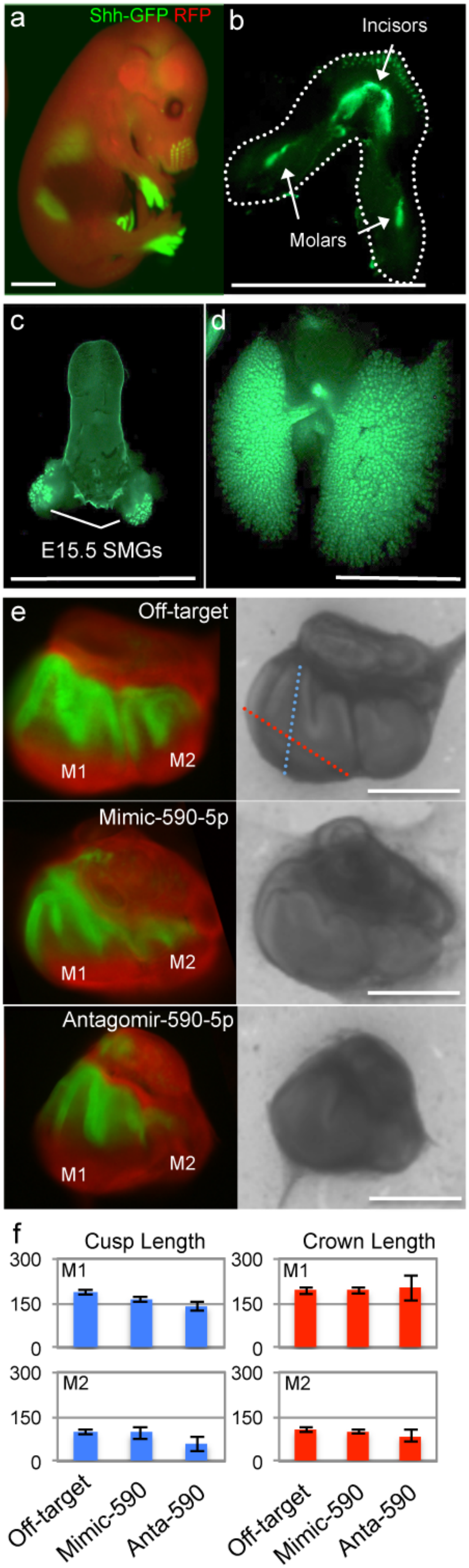

Supplementary Figure 14

a

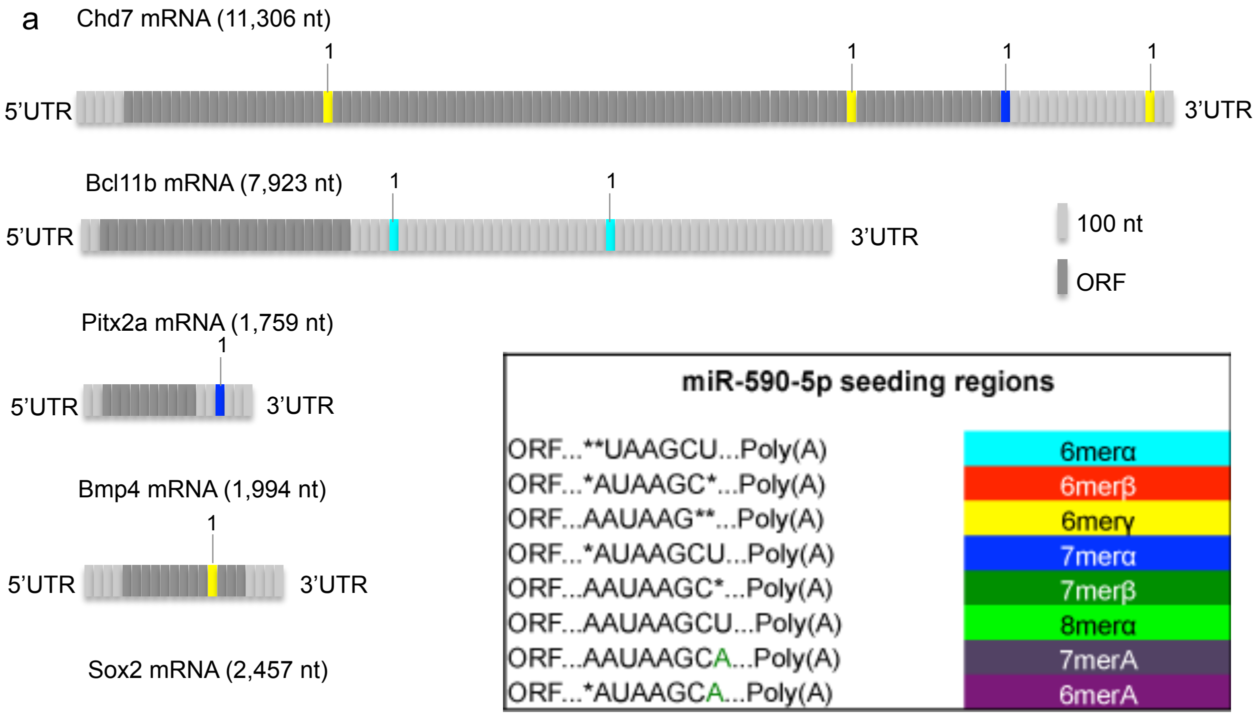

For seeding regions definition, refer to Grimson et al, *Molec.Cell* 2007

b

Examples of Synthetic Antagomir and Mimic

5' GAGCUUAUUCAUAAAAGUGCAG 3' → Mature miR-590-5p
3' CUCGAAUAAGUAUUUUCACGUC 5' → miR-590-5p Antagomir
5' GAGCUUAUUCAUAAAAGUGCAG 3' → miR-590-5p Mimic

c

Hydrophobic fragment — Hydrophilic fragment

GALFLGFLGA AGSTMGAWSQ**PKKKRKV**

HIV-1 gp41 protein — SV40 large T antigen

Supplementary Table 1 – TLDA Raw data and miRNA signature in multiple embryonic organs

Supplementary Table 1.1.1 Raw CT data E13.5 Molar Tooth Germs (NA = Not Available)

Well	miRNA ID	Task	CT AVG	ST DEV	Delta CT (Normalized to MammU6-4395470)	ST DEV
1	mmu-let-7b-4373168	Target	21.98	0.31	8.01	0.35
2	mmu-let-7c-4373167	Target	19.98	0.31	6.01	0.35
3	mmu-let-7d-4395394	Target	21.95	0.30	7.98	0.34
4	mmu-let-7e-4395517	Target	19.99	0.27	6.02	0.32
5	mmu-let-7g-4395393	Target	22.98	0.31	9.00	0.35
6	mmu-let-7i-4395332	Target	19.95	0.33	5.98	0.36
7	mmu-miR-1-4395333	Target	26.98	0.19	13.01	0.25
8	rno-miR-1-4395765	Target	26.97	0.30	13.00	0.34
9	mmu-miR-7a-4378130	Target	29.97	0.24	16.00	0.29
10	mmu-miR-7b-4395685	Target	31.97	0.20	18.00	0.26
11	MammU6-4395470	Control	13.98	0.16	0.00	0.23
12	MammU6-4395470	Control	13.73	0.17	-0.25	0.24
13	mmu-miR-9-4373285	Target	24.99	0.21	11.02	0.27
14	mmu-miR-10a-4373153	Target	27.91	0.08	13.94	0.18
15	mmu-miR-10b-4395329	Target	29.99	0.12	16.02	0.20
16	mmu-miR-15a-4373123	Target	23.97	0.15	9.99	0.22
17	mmu-miR-15b-4373122	Target	20.98	0.32	7.01	0.36
18	mmu-miR-16-4373121	Target	19.99	0.30	6.01	0.34
19	rno-miR-17-3p-4395779	Target	NA	NA	NA	NA
20	mmu-miR-18a-4395533	Target	22.97	0.30	8.99	0.34
21	mmu-miR-18b-4395596	Target	NA	NA	NA	NA
22	mmu-miR-19a-4373099	Target	19.96	0.23	5.99	0.28
23	mmu-miR-19b-4373098	Target	14.99	0.24	1.01	0.29
24	mmu-miR-20a-4373286	Target	17.99	0.28	4.02	0.32
25	mmu-miR-20b-4373263	Target	21.96	0.38	7.98	0.41
26	rno-miR-20b-3p-4395753	Target	NA	NA	NA	NA
27	mmu-miR-21-4373090	Target	22.98	0.28	9.01	0.33
28	mmu-miR-23b-4373073	Target	24.96	0.28	10.99	0.32
29	mmu-miR-24-4373072	Target	16.98	0.26	3.01	0.31
30	mmu-miR-25-4373071	Target	21.95	0.42	7.97	0.45
31	mmu-miR-26a-4395166	Target	18.98	0.27	5.00	0.32
32	mmu-miR-26b-4395167	Target	20.96	0.31	6.98	0.35
33	mmu-miR-27a-4373287	Target	22.98	0.40	9.01	0.43
34	mmu-miR-27b-4373068	Target	21.96	0.42	7.98	0.45
35	MammU6-4395470	Control	13.63	0.20	-0.34	0.26
36	MammU6-4395470	Control	13.77	0.17	-0.20	0.24
37	mmu-miR-28-4373067	Target	22.97	0.23	9.00	0.28
38	mmu-miR-29b-4373288	Target	30.97	0.26	16.99	0.31
39	mmu-miR-29c-4395171	Target	28.98	0.34	15.01	0.37
40	mmu-miR-30b-4373290	Target	17.98	0.36	4.00	0.39
41	mmu-miR-30c-4373060	Target	17.96	0.25	3.99	0.30
42	mmu-miR-30d-4373059	Target	23.97	0.25	9.99	0.30
43	mmu-miR-32-4395220	Target	27.97	0.31	14.00	0.35
44	mmu-miR-34a-4395168	Target	23.95	0.32	9.98	0.36
45	mmu-miR-34c-4373036	Target	29.01	0.23	15.03	0.28
46	mmu-miR-93-4373302	Target	20.96	0.37	6.99	0.40
47	mmu-miR-96-4373372	Target	26.97	0.35	12.99	0.39
48	mmu-miR-98-4373009	Target	23.97	0.10	10.00	0.19
49	mmu-miR-99a-4373008	Target	19.96	0.20	5.98	0.26
50	mmu-miR-99b-4373007	Target	18.98	0.13	5.01	0.21
51	mmu-miR-100-4373160	Target	20.00	0.23	6.02	0.28
52	mmu-miR-101a-4395364	Target	23.97	0.36	9.99	0.40
53	mmu-miR-103-4373158	Target	22.99	0.18	9.02	0.24

54	mmu-miR-105-4395595	Target	21.68	0.01	7.70	0.16
55	mmu-miR-107-4373154	Target	26.99	0.23	13.01	0.28
56	mmu-miR-122-4395356	Target	29.88	0.05	15.91	0.17
57	mmu-miR-124-4373295	Target	27.82	0.03	13.85	0.17
58	mmu-miR-125a-3p-4395310	Target	27.98	0.20	14.00	0.26
59	mmu-miR-125a-5p-4395309	Target	21.99	0.33	8.01	0.36
60	mmu-miR-125b-5p-4373148	Target	17.98	0.32	4.01	0.36
61	mmu-miR-126-3p-4395339	Target	18.99	0.27	5.02	0.32
62	mmu-miR-126-5p-4373269	Target	21.94	0.36	7.96	0.39
63	mmu-miR-127-4373147	Target	18.96	0.35	4.99	0.38
64	mmu-miR-128a-4395327	Target	25.97	0.23	11.99	0.28
65	mmu-miR-129-3p-4373297	Target	27.96	0.17	13.98	0.24
66	mmu-miR-130a-4373145	Target	20.97	0.30	7.00	0.34
67	mmu-miR-130b-4373144	Target	21.98	0.24	8.00	0.29
68	mmu-miR-132-4373143	Target	23.99	0.31	10.01	0.35
69	mmu-miR-133a-4395357	Target	22.95	0.18	8.98	0.25
70	mmu-miR-133b-4395358	Target	25.01	0.16	11.04	0.23
71	mmu-miR-134-4373299	Target	23.98	0.13	10.00	0.21
72	mmu-miR-135a-4373140	Target	25.99	0.25	12.02	0.30
73	mmu-miR-135b-4395372	Target	22.97	0.34	9.00	0.38
74	mmu-miR-136-4395641	Target	24.99	0.30	11.01	0.34
75	mmu-miR-137-4373301	Target	24.96	0.36	10.98	0.39
76	mmu-miR-138-4395395	Target	23.94	0.40	9.97	0.43
77	mmu-miR-139-3p-4395676	Target	22.29	0.01	8.31	0.16
78	mmu-miR-139-5p-4395400	Target	23.99	0.26	10.02	0.31
79	mmu-miR-140-4373374	Target	21.97	0.33	7.99	0.37
80	mmu-miR-141-4373137	Target	23.95	0.28	9.97	0.32
81	mmu-miR-142-3p-4373136	Target	23.97	0.34	10.00	0.38
82	mmu-miR-142-5p-4395359	Target	30.99	0.13	17.01	0.21
83	snoRNA135-4380912	Target	19.97	0.26	6.00	0.31
84	mmu-miR-145-4395389	Target	19.98	0.26	6.00	0.31
85	mmu-miR-146a-4373132	Target	23.96	0.34	9.98	0.38
86	mmu-miR-146b-4373178	Target	24.95	0.30	10.98	0.34
87	mmu-miR-147-4395373	Target	NA	NA	NA	NA
88	mmu-miR-148a-4373130	Target	21.97	0.28	7.99	0.32
89	mmu-miR-148b-4373129	Target	26.00	0.17	12.02	0.23
90	mmu-miR-150-4373127	Target	24.96	0.35	10.99	0.38
91	mmu-miR-151-3p-4373304	Target	24.97	0.14	10.99	0.22
92	mmu-miR-152-4395170	Target	20.96	0.45	6.98	0.48
93	mmu-miR-153-4373305	Target	35.99	0.06	22.01	0.17
94	mmu-miR-154-4373270	Target	27.93	0.07	13.95	0.18
95	mmu-miR-181a-4373117	Target	20.02	0.13	6.05	0.21
96	mmu-miR-181c-4373115	Target	23.96	0.09	9.98	0.19
97	mmu-miR-182-4395729	Target	19.97	0.29	6.00	0.33
98	mmu-miR-183-4395380	Target	23.01	0.11	9.04	0.20
99	mmu-miR-184-4373113	Target	25.96	0.27	11.98	0.32
100	mmu-miR-185-4395382	Target	27.00	0.23	13.02	0.28
101	mmu-miR-186-4395396	Target	23.96	0.34	9.98	0.38
102	mmu-miR-190-4373110	Target	26.98	0.28	13.01	0.32
103	mmu-miR-191-4395410	Target	20.00	0.35	6.03	0.39
104	mmu-miR-192-4373108	Target	24.97	0.32	10.99	0.36
105	mmu-miR-193-4395361	Target	30.98	0.17	17.00	0.24
106	mmu-miR-193b-4395597	Target	19.96	0.28	5.98	0.32
107	mmu-miR-194-4373106	Target	25.98	0.27	12.00	0.32
108	mmu-miR-195-4373105	Target	23.98	0.30	10.01	0.34
109	mmu-miR-196b-4395326	Target	NA	NA	NA	NA
110	rno-miR-196c-4395750	Target	5.26	0.01	-8.72	0.16
111	mmu-miR-197-4373102	Target	29.06	0.03	15.09	0.17
112	mmu-miR-199a-3p-4395415	Target	17.94	0.31	3.97	0.35

113	mmu-miR-199a-5p-4373272	Target	23.01	0.13	9.03	0.21
114	mmu-miR-200a-4378069	Target	20.97	0.32	6.99	0.36
115	mmu-miR-200b-4395362	Target	21.03	0.21	7.05	0.26
116	mmu-miR-200c-4395411	Target	19.98	0.26	6.01	0.30
117	mmu-miR-201-4395708	Target	25.61	0.00	11.64	0.16
118	mmu-miR-202-3p-4373311	Target	31.00	0.04	17.02	0.17
119	mmu-miR-202-5p-4395709	Target	NA	NA	NA	NA
120	mmu-miR-203-4373095	Target	20.98	0.28	7.01	0.32
121	mmu-miR-204-4373094	Target	23.96	0.28	9.99	0.33
122	mmu-miR-205-4373093	Target	19.01	0.26	5.03	0.31
123	mmu-miR-207-4373314	Target	NA	NA	NA	NA
124	rno-miR-207-4381096	Target	25.99	0.04	12.02	0.17
125	mmu-miR-208-4373091	Target	26.18	0.01	12.21	0.16
126	mmu-miR-208b-4395401	Target	NA	NA	NA	NA
127	mmu-miR-210-4373089	Target	20.99	0.14	7.01	0.21
128	mmu-miR-211-4373315	Target	24.91	0.10	10.93	0.19
129	mmu-miR-214-4395417	Target	17.98	0.33	4.00	0.37
130	mmu-miR-215-4373316	Target	36.03	0.06	22.06	0.17
131	snoRNA202-4380914	Target	15.95	0.40	1.98	0.43
132	mmu-miR-216a-4395331	Target	28.95	0.01	14.98	0.16
133	mmu-miR-216b-4395437	Target	29.00	0.17	15.02	0.24
134	mmu-miR-217-4395686	Target	31.04	0.07	17.07	0.18
135	mmu-miR-218-4373081	Target	17.95	0.40	3.98	0.43
136	mmu-miR-219-4373080	Target	28.98	0.10	15.00	0.19
137	mmu-miR-220-4395598	Target	NA	NA	NA	NA
138	mmu-miR-221-4373077	Target	23.98	0.28	10.00	0.32
139	mmu-miR-222-4395387	Target	23.95	0.33	9.98	0.37
140	mmu-miR-223-4395406	Target	23.98	0.31	10.00	0.35
141	mmu-miR-224-4395683	Target	24.98	0.31	11.00	0.35
142	mmu-miR-290-3p-4395721	Target	NA	NA	NA	NA
143	mmu-miR-291a-3p-4395722	Target	NA	NA	NA	NA
144	mmu-miR-291b-5p-4395667	Target	NA	NA	NA	NA
145	mmu-miR-292-3p-4395723	Target	30.97	0.27	16.99	0.32
146	mmu-miR-293-4386754	Target	33.02	0.11	19.04	0.20
147	mmu-miR-294-4373326	Target	33.00	0.08	19.02	0.18
148	mmu-miR-295-4373327	Target	32.97	0.24	19.00	0.29
149	mmu-miR-296-3p-4395212	Target	26.97	0.15	12.99	0.22
150	mmu-miR-296-5p-4373066	Target	21.01	0.17	7.03	0.23
151	mmu-miR-297c-4395610	Target	29.31	0.02	15.34	0.16
152	mmu-miR-297b-5p-4381049	Target	30.95	0.06	16.97	0.17
153	mmu-miR-298-4395728	Target	23.97	0.21	10.00	0.27
154	mmu-miR-301a-4373064	Target	19.99	0.24	6.01	0.29
155	mmu-miR-301b-4395730	Target	21.00	0.29	7.02	0.34
156	mmu-miR-302a-4378070	Target	31.98	0.14	18.00	0.22
157	mmu-miR-302c-4395688	Target	NA	NA	NA	NA
158	mmu-miR-302d-4373063	Target	31.00	0.16	17.03	0.23
159	mmu-miR-320-4395388	Target	22.97	0.31	9.00	0.35
160	mmu-miR-323-3p-4395338	Target	25.97	0.18	11.99	0.24
161	mmu-miR-324-5p-4373052	Target	22.95	0.26	8.98	0.31
162	mmu-miR-325-4395640	Target	23.56	0.01	9.59	0.16
163	rno-miR-327-4381108	Target	34.10	0.02	20.13	0.16
164	mmu-miR-328-4373049	Target	20.97	0.23	7.00	0.28
165	mmu-miR-329-4373336	Target	26.97	0.29	12.99	0.34
166	mmu-miR-330-4395341	Target	29.91	0.03	15.94	0.17
167	mmu-miR-331-3p-4373046	Target	21.00	0.24	7.02	0.29
168	mmu-miR-331-5p-4395344	Target	29.02	0.15	15.04	0.22
169	rno-miR-333-4381109	Target	32.08	0.01	18.10	0.16
170	mmu-miR-335-3p-4395296	Target	21.98	0.39	8.01	0.42
171	mmu-miR-335-5p-4373045	Target	19.97	0.23	6.00	0.29

172	rno-miR-336-4381111	Target	34.76	0.00	20.79	0.16
173	mmu-miR-337-3p-4395662	Target	26.97	0.28	13.00	0.32
174	mmu-miR-337-5p-4395645	Target	24.97	0.30	11.00	0.34
175	mmu-miR-338-3p-4395363	Target	30.98	0.18	17.00	0.24
176	mmu-miR-339-3p-4395663	Target	26.06	0.09	12.08	0.19
177	rno-miR-339-3p-4395760	Target	26.02	0.06	12.05	0.17
178	mmu-miR-339-5p-4395368	Target	25.00	0.11	11.02	0.20
179	U87-4386735	Target	21.97	0.21	8.00	0.26
180	mmu-miR-340-3p-4395370	Target	24.96	0.32	10.98	0.36
181	mmu-miR-340-5p-4395369	Target	23.98	0.35	10.00	0.39
182	mmu-miR-342-3p-4395371	Target	20.99	0.23	7.01	0.28
183	mmu-miR-342-5p-4395657	Target	28.99	0.10	15.02	0.19
184	rno-miR-343-4381123	Target	NA	NA	NA	NA
185	mmu-miR-344-4373340	Target	28.00	0.12	14.02	0.20
186	rno-miR-344-3p-4381112	Target	NA	NA	NA	NA
187	rno-miR-344-5p-4395761	Target	NA	NA	NA	NA
188	mmu-miR-345-3p-4395659	Target	28.97	0.16	15.00	0.23
189	rno-miR-345-3p-4395762	Target	27.97	0.29	13.99	0.33
190	mmu-miR-345-5p-4395658	Target	25.98	0.19	12.01	0.25
191	mmu-miR-346-4373342	Target	NA	NA	NA	NA
192	rno-miR-346-4381113	Target	25.98	0.01	12.01	0.16
193	rno-miR-347-4381114	Target	NA	NA	NA	NA
194	rno-miR-349-4381115	Target	NA	NA	NA	NA
195	mmu-miR-350-4395660	Target	25.98	0.28	12.00	0.33
196	rno-miR-351-4395764	Target	23.99	0.20	10.02	0.25
197	mmu-miR-361-4373035	Target	25.97	0.30	12.00	0.35
198	mmu-miR-362-3p-4395746	Target	26.96	0.26	12.99	0.31
199	mmu-miR-363-4378090	Target	33.97	0.03	19.99	0.17
200	mmu-miR-365-4373194	Target	21.98	0.21	8.01	0.27
201	mmu-miR-367-4373034	Target	31.97	0.19	17.99	0.25
202	mmu-miR-369-3p-4373032	Target	28.98	0.36	15.00	0.39
203	mmu-miR-369-5p-4373195	Target	26.95	0.26	12.97	0.31
204	mmu-miR-370-4395386	Target	22.99	0.23	9.01	0.28
205	mmu-miR-375-4373027	Target	24.98	0.30	11.01	0.34
206	mmu-miR-376a-4373347	Target	23.99	0.29	10.01	0.33
207	mmu-miR-376b-4395582	Target	23.94	0.10	9.96	0.19
208	mmu-miR-376c-4395580	Target	22.96	0.30	8.98	0.34
209	mmu-miR-377-4373025	Target	32.01	0.06	18.04	0.17
210	rno-miR-377-4381100	Target	NA	NA	NA	NA
211	mmu-miR-379-4373349	Target	21.95	0.29	7.97	0.33
212	mmu-miR-380-3p-4373350	Target	31.00	0.11	17.03	0.20
213	mmu-miR-380-5p-4395731	Target	25.99	0.28	12.02	0.32
214	mmu-miR-381-4373020	Target	29.96	0.33	15.98	0.36
215	mmu-miR-382-4373019	Target	20.99	0.23	7.02	0.28
216	mmu-miR-383-4381093	Target	28.97	0.25	14.99	0.30
217	mmu-miR-384-3p-4395733	Target	29.98	0.18	16.00	0.24
218	mmu-miR-384-5p-4395732	Target	26.95	0.30	12.97	0.34
219	mmu-miR-409-3p-4395443	Target	21.97	0.27	8.00	0.31
220	mmu-miR-409-5p-4395442	Target	26.07	0.10	12.10	0.19
221	mmu-miR-410-4378093	Target	22.96	0.29	8.99	0.33
222	mmu-miR-411-4381013	Target	19.96	0.33	5.99	0.37
223	rno-miR-421-4381122	Target	NA	NA	NA	NA
224	mmu-miR-423-5p-4395451	Target	25.98	0.16	12.01	0.23
225	mmu-miR-425-4380926	Target	22.06	0.03	8.09	0.17
226	mmu-miR-429-4373355	Target	20.96	0.25	6.98	0.30
227	Y1-4386739	Target	21.00	0.20	7.02	0.26
228	mmu-miR-431-4395173	Target	23.00	0.23	9.03	0.28
229	mmu-miR-433-4373205	Target	24.02	0.21	10.05	0.27
230	mmu-miR-434-3p-4395734	Target	21.98	0.32	8.01	0.36

231	mmu-miR-434-5p-4395711	Target	25.96	0.24	11.99	0.29
232	mmu-miR-448-4373206	Target	32.94	0.10	18.96	0.19
233	mmu-miR-449a-4373207	Target	30.96	0.27	16.99	0.31
234	mmu-miR-449b-4395669	Target	NA	NA	NA	NA
235	mmu-miR-450a-5p-4395414	Target	26.97	0.32	12.99	0.36
236	mmu-miR-450b-5p-4386779	Target	NA	NA	NA	NA
237	mmu-miR-451-4373360	Target	25.95	0.35	11.98	0.38
238	mmu-miR-453-4395614	Target	33.10	0.02	19.13	0.16
239	mmu-miR-455-4395585	Target	24.01	0.28	10.03	0.33
240	mmu-miR-465a-3p-4395574	Target	32.97	0.20	18.99	0.25
241	mmu-miR-465a-5p-4373363	Target	NA	NA	NA	NA
242	mmu-miR-465b-5p-4395615	Target	32.99	0.03	19.02	0.17
243	rno-miR-466b-4395767	Target	NA	NA	NA	NA
244	rno-miR-466c-4395768	Target	31.39	0.03	17.42	0.17
245	mmu-miR-466h-4395646	Target	27.26	0.02	13.28	0.16
246	mmu-miR-467a-4395717	Target	24.99	0.32	11.01	0.36
247	mmu-miR-467c-4395647	Target	26.00	0.22	12.02	0.27
248	mmu-miR-467d-4395648	Target	28.99	0.25	15.02	0.30
249	mmu-miR-467e-4395698	Target	27.99	0.22	14.01	0.27
250	mmu-miR-470-4395718	Target	NA	NA	NA	NA
251	mmu-miR-484-4381032	Target	18.96	0.35	4.98	0.39
252	mmu-miR-486-4378096	Target	27.70	0.01	13.72	0.16
253	mmu-miR-487b-4378102	Target	35.97	0.08	21.99	0.18
254	mmu-miR-489-4378114	Target	30.97	0.25	16.99	0.30
255	mmu-miR-491-4381053	Target	27.97	0.23	13.99	0.28
256	mmu-miR-493-4395649	Target	32.00	0.13	18.02	0.21
257	mmu-miR-494-4395476	Target	24.98	0.27	11.00	0.32
258	mmu-miR-495-4381078	Target	22.98	0.32	9.00	0.36
259	mmu-miR-497-4381046	Target	26.00	0.31	12.03	0.35
260	mmu-miR-499-4381047	Target	33.18	0.01	19.20	0.16
261	mmu-miR-500-4395736	Target	26.99	0.19	13.02	0.25
262	mmu-miR-501-3p-4381069	Target	23.99	0.09	10.01	0.18
263	mmu-miR-503-4395586	Target	23.98	0.31	10.01	0.35
264	mmu-miR-504-4395195	Target	27.98	0.05	14.00	0.17
265	mmu-miR-505-4381071	Target	NA	NA	NA	NA
266	mmu-miR-509-3p-4395651	Target	36.02	0.01	22.05	0.16
267	mmu-miR-509-5p-4395650	Target	NA	NA	NA	NA
268	mmu-miR-511-4395679	Target	31.00	0.05	17.03	0.17
269	mmu-miR-532-3p-4395466	Target	22.98	0.26	9.00	0.31
270	mmu-miR-532-5p-4380928	Target	22.98	0.37	9.00	0.41
271	rno-miR-532-5p-4395752	Target	23.96	0.07	9.98	0.18
272	mmu-miR-539-4378103	Target	32.95	0.15	18.97	0.22
273	mmu-miR-540-3p-4378119	Target	NA	NA	NA	NA
274	mmu-miR-540-5p-4395691	Target	29.97	0.08	16.00	0.18
275	ath-miR159a-4373390	Target	NA	NA	NA	NA
276	mmu-miR-542-3p-4378101	Target	27.98	0.28	14.01	0.33
277	mmu-miR-542-5p-4395693	Target	28.98	0.14	15.01	0.22
278	mmu-miR-543-4395487	Target	24.96	0.28	10.99	0.33
279	rno-miR-543-4395766	Target	NA	NA	NA	NA
280	mmu-miR-544-4395680	Target	27.97	0.29	13.99	0.33
281	mmu-miR-551b-4380945	Target	31.98	0.27	18.01	0.32
282	mmu-miR-574-3p-4395460	Target	22.97	0.34	8.99	0.38
283	mmu-miR-582-3p-4395697	Target	31.95	0.16	17.98	0.23
284	mmu-miR-582-5p-4395696	Target	28.96	0.13	14.99	0.21
285	mmu-miR-590-5p-4395176	Target	29.84	0.01	15.86	0.16
286	mmu-miR-598-4395606	Target	28.97	0.22	15.00	0.27
287	rno-miR-598-5p-4395754	Target	32.96	0.02	18.98	0.16
288	mmu-miR-615-3p-4386777	Target	NA	NA	NA	NA
289	mmu-miR-615-5p-4395464	Target	NA	NA	NA	NA

290	mmu-miR-654-3p-4395350	Target	NA	NA	NA	NA
291	mmu-miR-654-5p-4395652	Target	NA	NA	NA	NA
292	mmu-miR-665-4395737	Target	29.99	0.17	16.02	0.24
293	mmu-miR-666-5p-4386770	Target	28.93	0.07	14.96	0.18
294	mmu-miR-669a-4381091	Target	27.01	0.24	13.04	0.29
295	mmu-miR-671-3p-4395433	Target	25.97	0.33	12.00	0.37
296	mmu-miR-672-4395438	Target	24.97	0.23	10.99	0.28
297	rno-miR-673-4395755	Target	NA	NA	NA	NA
298	mmu-miR-674-4395193	Target	24.92	0.18	10.95	0.24
299	mmu-miR-708-4395452	Target	22.96	0.36	8.98	0.40
300	mmu-miR-741-4395587	Target	32.02	0.06	18.04	0.17
301	mmu-miR-742-4395573	Target	NA	NA	NA	NA
302	rno-miR-742-4395756	Target	NA	NA	NA	NA
303	mmu-miR-743b-3p-4395601	Target	31.76	0.01	17.79	0.16
304	mmu-miR-743b-5p-4395600	Target	28.19	0.01	14.21	0.16
305	mmu-miR-743a-4395599	Target	26.74	0.01	12.76	0.16
306	rno-miR-743b-4395769	Target	33.06	0.01	19.09	0.16
307	mmu-miR-744-4395435	Target	22.97	0.38	9.00	0.41
308	rno-miR-758-4395180	Target	27.98	0.21	14.01	0.27
309	rno-miR-760-5p-4395758	Target	NA	NA	NA	NA
310	mmu-miR-770-3p-4395564	Target	29.96	0.03	15.98	0.17
311	mmu-miR-802-4395566	Target	34.97	0.06	21.00	0.17
312	mmu-miR-871-4395465	Target	NA	NA	NA	NA
313	rno-miR-871-4395770	Target	NA	NA	NA	NA
314	mmu-miR-872-4395375	Target	24.96	0.34	10.99	0.37
315	mmu-miR-873-4395467	Target	34.01	0.10	20.04	0.19
316	mmu-miR-874-4395379	Target	NA	NA	NA	NA
317	mmu-miR-875-3p-4395677	Target	26.98	0.26	13.01	0.31
318	mmu-miR-876-3p-4395594	Target	NA	NA	NA	NA
319	mmu-miR-876-5p-4395593	Target	NA	0.00	NA	0.16
320	rno-miR-878-4395771	Target	32.55	0.01	18.58	0.16
321	mmu-miR-878-5p-4395670	Target	NA	NA	NA	NA
322	mmu-miR-879-4395602	Target	34.00	0.09	20.02	0.19
323	mmu-miR-881-4395739	Target	NA	NA	NA	NA
324	rno-miR-881-4395773	Target	NA	NA	NA	NA
325	mmu-miR-883a-3p-4395591	Target	NA	NA	NA	NA
326	mmu-miR-883b-3p-4395695	Target	NA	NA	NA	NA
327	mmu-miR-883a-5p-4395741	Target	NA	NA	NA	NA
328	mmu-miR-125b-3p-4395489	Target	26.97	0.17	12.99	0.24
329	mmu-miR-143-4395360	Target	22.97	0.33	8.99	0.37
330	rno-miR-219-1-3p-4395778	Target	28.98	0.06	15.01	0.17
331	rno-miR-219-2-3p-4395501	Target	NA	NA	NA	NA
332	rno-miR-224-4373187	Target	23.97	0.20	9.99	0.26
333	mmu-miR-324-3p-4395639	Target	23.98	0.22	10.01	0.27
334	mmu-miR-351-4373345	Target	22.97	0.17	9.00	0.24
335	rno-miR-381-4381102	Target	28.00	0.22	14.02	0.27
336	rno-miR-409-5p-4381098	Target	NA	NA	NA	NA
337	rno-miR-450a-4381124	Target	29.00	0.16	15.03	0.23
338	mmu-miR-452-4373281	Target	NA	NA	NA	NA
339	mmu-miR-464-4373362	Target	NA	NA	NA	NA
340	mmu-miR-467b-4381084	Target	25.98	0.19	12.01	0.25
341	mmu-miR-468-4373366	Target	NA	NA	NA	NA
342	mmu-miR-469-4373367	Target	NA	NA	NA	NA
343	mmu-miR-488-4381074	Target	29.93	0.12	15.96	0.20
344	mmu-miR-490-4373215	Target	NA	NA	NA	NA
345	mmu-miR-496-4386771	Target	29.99	0.12	16.01	0.20
346	rno-miR-505-4381097	Target	NA	NA	NA	NA
347	mmu-miR-546-4381044	Target	31.82	0.01	17.84	0.16
348	mmu-miR-547-4395694	Target	32.03	0.08	18.05	0.18

349	mmu-miR-652-4395463	Target	22.99	0.28	9.02	0.32
350	mmu-miR-667-4386769	Target	24.97	0.24	10.99	0.29
351	mmu-miR-668-4386767	Target	26.97	0.13	12.99	0.21
352	mmu-miR-670-4395561	Target	35.09	0.01	21.11	0.16
353	mmu-miR-675-3p-4386762	Target	25.99	0.18	12.02	0.25
354	mmu-miR-675-5p-4386761	Target	NA	NA	NA	NA
355	mmu-miR-676-4386776	Target	22.99	0.29	9.01	0.33
356	mmu-miR-677-4381075	Target	32.97	0.09	19.00	0.19
357	mmu-miR-679-4381077	Target	31.00	0.05	17.02	0.17
358	mmu-miR-680-4381079	Target	28.96	0.03	14.98	0.17
359	mmu-miR-682-4381081	Target	28.95	0.04	14.98	0.17
360	mmu-miR-683-4381082	Target	NA	NA	NA	NA
361	mmu-miR-684-4381083	Target	32.93	0.11	18.95	0.20
362	mmu-miR-685-4386748	Target	25.90	0.06	11.93	0.17
363	mmu-miR-686-4381085	Target	NA	NA	NA	NA
364	mmu-miR-687-4386750	Target	24.85	0.01	10.87	0.16
365	mmu-let-7a-4373169	Target	21.01	0.15	7.03	0.22
366	mmu-let-7f-4373164	Target	22.99	0.13	9.02	0.21
367	mmu-miR-106b-4373155	Target	19.96	0.28	5.98	0.32
368	mmu-miR-155-4395701	Target	28.94	0.31	14.97	0.35
369	mmu-miR-17-4395419	Target	17.96	0.34	3.98	0.38
370	mmu-miR-23a-4373074	Target	24.01	0.01	10.04	0.16
371	mmu-miR-29a-4395223	Target	24.93	0.26	10.95	0.31
372	mmu-miR-30a-4373061	Target	21.95	0.31	7.97	0.35
373	mmu-miR-30e-4395334	Target	22.97	0.33	8.99	0.37
374	mmu-miR-31-4373331	Target	19.98	0.29	6.01	0.33
375	mmu-miR-34b-3p-4395748	Target	26.97	0.28	13.00	0.32
376	mmu-miR-92a-4373013	Target	18.96	0.36	4.99	0.39
377	rno-miR-190b-4395749	Target	28.97	0.30	14.99	0.34
378	mmu-miR-129-5p-4373171	Target	27.62	0.01	13.65	0.16
379	mmu-miR-187-4373307	Target	27.05	0.05	13.08	0.17
380	mmu-miR-188-3p-4395217	Target	NA	NA	NA	NA
381	mmu-miR-106a-4395589	Target	17.97	0.39	4.00	0.42
382	mmu-miR-188-5p-4395431	Target	26.95	0.32	12.98	0.36
383	mmu-miR-302b-4378071	Target	32.98	0.32	19.01	0.36
384	mmu-miR-322-4378107	Target	22.98	0.34	9.01	0.38

Supplementary Table 1.1.2 Heat Map for Molar Tooth Germ miRNA-Signature

Relative abundance:

High Low

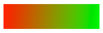

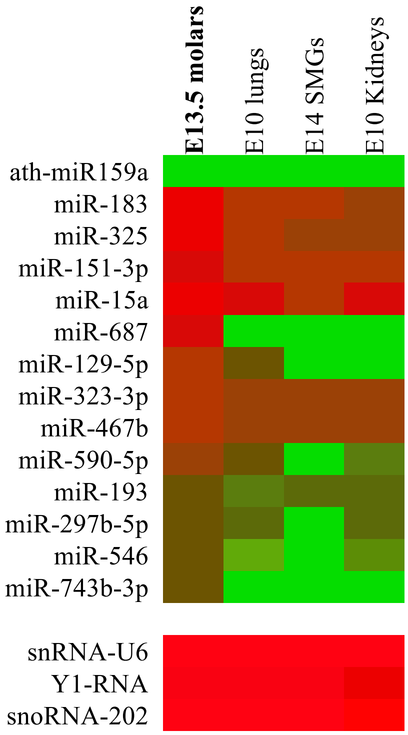

Supplementary Table 1.2.1 Raw CT data E13.5 Submandibular Salivary Glands (SMGs) (NA = Not Available)

Well	miRNA ID	Task	CT AVG	ST DEV	Delta CT (Normalized to MammU6-4395470)	ST DEV
1	mmu-let-7b-4373168	Target	17.39	0.31	6.30	0.36
2	mmu-let-7c-4373167	Target	15.95	0.33	4.86	0.38
3	mmu-let-7d-4395394	Target	18.76	0.31	7.66	0.35
4	mmu-let-7e-4395517	Target	16.59	0.28	5.50	0.33
5	mmu-let-7g-4395393	Target	19.04	0.33	7.94	0.38
6	mmu-let-7i-4395332	Target	17.21	0.33	6.12	0.37
7	mmu-miR-1-4395333	Target	22.78	0.20	11.69	0.26
8	rno-miR-1-4395765	Target	21.52	0.30	10.43	0.35
9	mmu-miR-7a-4378130	Target	23.41	0.29	12.32	0.34
10	mmu-miR-7b-4395685	Target	24.23	0.30	13.14	0.35
11	MammU6-4395470	Control	11.09	0.18	0.00	0.25
12	MammU6-4395470	Control	10.54	0.18	-0.56	0.25
13	mmu-miR-9-4373285	Target	19.26	0.25	8.16	0.30
14	mmu-miR-10a-4373153	Target	24.49	0.14	13.40	0.22
15	mmu-miR-10b-4395329	Target	23.34	0.26	12.25	0.31
16	mmu-miR-15a-4373123	Target	23.29	0.13	12.19	0.22
17	mmu-miR-15b-4373122	Target	18.17	0.32	7.08	0.36
18	mmu-miR-16-4373121	Target	17.37	0.33	6.28	0.37
19	rno-miR-17-3p-4395779	Target	NA	NA	NA	NA
20	mmu-miR-18a-4395533	Target	17.68	0.32	6.59	0.37
21	mmu-miR-18b-4395596	Target	31.51	0.04	20.42	0.18
22	mmu-miR-19a-4373099	Target	16.97	0.25	5.87	0.31
23	mmu-miR-19b-4373098	Target	12.96	0.27	1.87	0.33
24	mmu-miR-20a-4373286	Target	11.83	0.27	0.74	0.32
25	mmu-miR-20b-4373263	Target	18.59	0.36	7.49	0.40
26	rno-miR-20b-3p-4395753	Target	NA	NA	NA	NA
27	mmu-miR-21-4373090	Target	18.96	0.27	7.87	0.32
28	mmu-miR-23b-4373073	Target	20.37	0.22	9.28	0.28
29	mmu-miR-24-4373072	Target	12.66	0.26	1.57	0.31
30	mmu-miR-25-4373071	Target	14.94	0.41	3.85	0.44
31	mmu-miR-26a-4395166	Target	13.99	0.24	2.90	0.30
32	mmu-miR-26b-4395167	Target	13.86	0.26	2.77	0.31
33	mmu-miR-27a-4373287	Target	18.13	0.36	7.04	0.40
34	mmu-miR-27b-4373068	Target	15.88	0.41	4.79	0.44
35	MammU6-4395470	Control	10.43	0.20	-0.66	0.26
36	MammU6-4395470	Control	10.96	0.17	-0.13	0.25
37	mmu-miR-28-4373067	Target	15.75	0.27	4.66	0.32
38	mmu-miR-29b-4373288	Target	24.41	0.26	13.32	0.32
39	mmu-miR-29c-4395171	Target	21.56	0.38	10.47	0.42
40	mmu-miR-30b-4373290	Target	12.26	0.38	1.17	0.41
41	mmu-miR-30c-4373060	Target	11.31	0.29	0.22	0.34
42	mmu-miR-30d-4373059	Target	17.80	0.27	6.71	0.32
43	mmu-miR-32-4395220	Target	24.15	0.29	13.06	0.34
44	mmu-miR-34a-4395168	Target	19.84	0.25	8.75	0.31
45	mmu-miR-34c-4373036	Target	22.03	0.28	10.94	0.33
46	mmu-miR-93-4373302	Target	18.60	0.34	7.50	0.38
47	mmu-miR-96-4373372	Target	22.30	0.32	11.21	0.36
48	mmu-miR-98-4373009	Target	22.40	0.11	11.31	0.20
49	mmu-miR-99a-4373008	Target	18.22	0.17	7.13	0.24
50	mmu-miR-99b-4373007	Target	14.20	0.15	3.11	0.23
51	mmu-miR-100-4373160	Target	17.46	0.22	6.37	0.28
52	mmu-miR-101a-4395364	Target	18.52	0.36	7.43	0.40
53	mmu-miR-103-4373158	Target	18.57	0.19	7.48	0.26
54	mmu-miR-105-4395595	Target	NA	NA	NA	NA
55	mmu-miR-107-4373154	Target	23.82	0.19	12.73	0.26

56	mmu-miR-122-4395356	Target	29.91	0.05	18.82	0.18
57	mmu-miR-124-4373295	Target	29.04	0.07	17.95	0.19
58	mmu-miR-125a-3p-4395310	Target	24.70	0.20	13.61	0.27
59	mmu-miR-125a-5p-4395309	Target	15.92	0.30	4.83	0.35
60	mmu-miR-125b-5p-4373148	Target	11.21	0.31	0.12	0.35
61	mmu-miR-126-3p-4395339	Target	15.94	0.28	4.85	0.33
62	mmu-miR-126-5p-4373269	Target	15.31	0.32	4.22	0.36
63	mmu-miR-127-4373147	Target	11.20	0.38	0.11	0.41
64	mmu-miR-128a-4395327	Target	22.95	0.22	11.86	0.28
65	mmu-miR-129-3p-4373297	Target	24.91	0.19	13.82	0.26
66	mmu-miR-130a-4373145	Target	14.50	0.30	3.41	0.35
67	mmu-miR-130b-4373144	Target	16.42	0.25	5.33	0.30
68	mmu-miR-132-4373143	Target	16.17	0.33	5.07	0.37
69	mmu-miR-133a-4395357	Target	17.36	0.21	6.27	0.27
70	mmu-miR-133b-4395358	Target	21.80	0.16	10.71	0.24
71	mmu-miR-134-4373299	Target	19.73	0.17	8.64	0.24
72	mmu-miR-135a-4373140	Target	18.01	0.27	6.91	0.32
73	mmu-miR-135b-4395372	Target	20.04	0.27	8.95	0.33
74	mmu-miR-136-4395641	Target	17.70	0.40	6.61	0.44
75	mmu-miR-137-4373301	Target	21.28	0.32	10.19	0.36
76	mmu-miR-138-4395395	Target	16.94	0.38	5.85	0.42
77	mmu-miR-139-3p-4395676	Target	NA	NA	NA	NA
78	mmu-miR-139-5p-4395400	Target	16.98	0.27	5.89	0.32
79	mmu-miR-140-4373374	Target	18.95	0.30	7.86	0.35
80	mmu-miR-141-4373137	Target	20.96	0.21	9.87	0.27
81	mmu-miR-142-3p-4373136	Target	19.49	0.29	8.40	0.34
82	mmu-miR-142-5p-4395359	Target	27.93	0.15	16.84	0.23
83	snoRNA135-4380912	Target	17.09	0.23	6.00	0.29
84	mmu-miR-145-4395389	Target	16.18	0.26	5.09	0.31
85	mmu-miR-146a-4373132	Target	19.78	0.29	8.69	0.34
86	mmu-miR-146b-4373178	Target	22.14	0.23	11.04	0.29
87	mmu-miR-147-4395373	Target	NA	NA	NA	NA
88	mmu-miR-148a-4373130	Target	16.66	0.27	5.57	0.32
89	mmu-miR-148b-4373129	Target	22.02	0.17	10.92	0.24
90	mmu-miR-150-4373127	Target	18.13	0.32	7.04	0.37
91	mmu-miR-151-3p-4373304	Target	23.84	0.12	12.75	0.21
92	mmu-miR-152-4395170	Target	14.32	0.40	3.22	0.44
93	mmu-miR-153-4373305	Target	34.65	0.05	23.56	0.18
94	mmu-miR-154-4373270	Target	21.88	0.19	10.79	0.26
95	mmu-miR-181a-4373117	Target	14.37	0.15	3.28	0.23
96	mmu-miR-181c-4373115	Target	20.17	0.10	9.08	0.20
97	mmu-miR-182-4395729	Target	15.24	0.33	4.15	0.37
98	mmu-miR-183-4395380	Target	23.28	0.11	12.19	0.21
99	mmu-miR-184-4373113	Target	20.40	0.27	9.31	0.32
100	mmu-miR-185-4395382	Target	23.89	0.17	12.79	0.24
101	mmu-miR-186-4395396	Target	19.94	0.33	8.84	0.38
102	mmu-miR-190-4373110	Target	19.73	0.28	8.64	0.33
103	mmu-miR-191-4395410	Target	15.16	0.34	4.07	0.39
104	mmu-miR-192-4373108	Target	18.98	0.33	7.89	0.37
105	mmu-miR-193-4395361	Target	29.59	0.11	18.50	0.21
106	mmu-miR-193b-4395597	Target	16.14	0.28	5.05	0.33
107	mmu-miR-194-4373106	Target	22.66	0.24	11.57	0.30
108	mmu-miR-195-4373105	Target	21.42	0.26	10.32	0.32
109	mmu-miR-196b-4395326	Target	32.58	0.05	21.49	0.18
110	rno-miR-196c-4395750	Target	25.15	0.15	14.06	0.23
111	mmu-miR-197-4373102	Target	32.08	0.03	20.99	0.18
112	mmu-miR-199a-3p-4395415	Target	15.42	0.26	4.32	0.32
113	mmu-miR-199a-5p-4373272	Target	19.82	0.14	8.73	0.22
114	mmu-miR-200a-4378069	Target	17.02	0.27	5.93	0.32

115	mmu-miR-200b-4395362	Target	19.14	0.18	8.05	0.25
116	mmu-miR-200c-4395411	Target	17.88	0.22	6.79	0.28
117	mmu-miR-201-4395708	Target	NA	NA	NA	NA
118	mmu-miR-202-3p-4373311	Target	31.86	0.04	20.77	0.18
119	mmu-miR-202-5p-4395709	Target	NA	NA	NA	NA
120	mmu-miR-203-4373095	Target	14.96	0.33	3.87	0.37
121	mmu-miR-204-4373094	Target	16.51	0.31	5.42	0.35
122	mmu-miR-205-4373093	Target	16.05	0.30	4.95	0.35
123	mmu-miR-207-4373314	Target	NA	NA	NA	NA
124	rno-miR-207-4381096	Target	26.77	0.07	15.68	0.19
125	mmu-miR-208-4373091	Target	38.96	0.02	27.87	0.18
126	mmu-miR-208b-4395401	Target	NA	NA	NA	NA
127	mmu-miR-210-4373089	Target	19.45	0.13	8.35	0.22
128	mmu-miR-211-4373315	Target	NA	NA	NA	NA
129	mmu-miR-214-4395417	Target	15.83	0.25	4.74	0.30
130	mmu-miR-215-4373316	Target	31.28	0.11	20.19	0.21
131	snoRNA202-4380914	Target	13.35	0.32	2.26	0.36
132	mmu-miR-216a-4395331	Target	NA	NA	NA	NA
133	mmu-miR-216b-4395437	Target	27.92	0.10	16.83	0.20
134	mmu-miR-217-4395686	Target	34.06	0.04	22.97	0.18
135	mmu-miR-218-4373081	Target	11.77	0.34	0.68	0.39
136	mmu-miR-219-4373080	Target	31.03	0.05	19.94	0.18
137	mmu-miR-220-4395598	Target	NA	NA	NA	NA
138	mmu-miR-221-4373077	Target	17.90	0.31	6.81	0.35
139	mmu-miR-222-4395387	Target	19.78	0.31	8.69	0.36
140	mmu-miR-223-4395406	Target	19.60	0.29	8.51	0.34
141	mmu-miR-224-4395683	Target	20.23	0.32	9.14	0.37
142	mmu-miR-290-3p-4395721	Target	NA	NA	NA	NA
143	mmu-miR-291a-3p-4395722	Target	26.14	0.28	15.05	0.33
144	mmu-miR-291b-5p-4395667	Target	NA	NA	NA	NA
145	mmu-miR-292-3p-4395723	Target	18.84	0.40	7.75	0.44
146	mmu-miR-293-4386754	Target	24.01	0.27	12.92	0.32
147	mmu-miR-294-4373326	Target	25.36	0.25	14.27	0.31
148	mmu-miR-295-4373327	Target	21.85	0.35	10.76	0.39
149	mmu-miR-296-3p-4395212	Target	24.11	0.15	13.02	0.23
150	mmu-miR-296-5p-4373066	Target	15.57	0.17	4.48	0.24
151	mmu-miR-297c-4395610	Target	NA	NA	NA	NA
152	mmu-miR-297b-5p-4381049	Target	NA	NA	NA	NA
153	mmu-miR-298-4395728	Target	22.52	0.20	11.43	0.27
154	mmu-miR-301a-4373064	Target	17.47	0.26	6.38	0.31
155	mmu-miR-301b-4395730	Target	15.19	0.28	4.10	0.33
156	mmu-miR-302a-4378070	Target	25.06	0.20	13.97	0.26
157	mmu-miR-302c-4395688	Target	NA	NA	NA	NA
158	mmu-miR-302d-4373063	Target	25.24	0.21	14.15	0.28
159	mmu-miR-320-4395388	Target	18.90	0.27	7.80	0.32
160	mmu-miR-323-3p-4395338	Target	24.74	0.17	13.65	0.25
161	mmu-miR-324-5p-4373052	Target	15.82	0.23	4.73	0.29
162	mmu-miR-325-4395640	Target	25.76	0.12	14.67	0.21
163	rno-miR-327-4381108	Target	NA	NA	NA	NA
164	mmu-miR-328-4373049	Target	18.07	0.20	6.98	0.27
165	mmu-miR-329-4373336	Target	18.34	0.32	7.25	0.36
166	mmu-miR-330-4395341	Target	29.34	0.09	18.25	0.20
167	mmu-miR-331-3p-4373046	Target	15.91	0.29	4.82	0.34
168	mmu-miR-331-5p-4395344	Target	27.89	0.10	16.80	0.20
169	rno-miR-333-4381109	Target	33.13	0.06	22.04	0.19
170	mmu-miR-335-3p-4395296	Target	16.21	0.35	5.12	0.39
171	mmu-miR-335-5p-4373045	Target	13.28	0.23	2.19	0.29
172	rno-miR-336-4381111	Target	NA	NA	NA	NA
173	mmu-miR-337-3p-4395662	Target	18.96	0.29	7.87	0.34

174	mmu-miR-337-5p-4395645	Target	16.54	0.28	5.45	0.33
175	mmu-miR-338-3p-4395363	Target	28.70	0.17	17.61	0.24
176	mmu-miR-339-3p-4395663	Target	29.51	0.05	18.42	0.18
177	rno-miR-339-3p-4395760	Target	28.20	0.06	17.11	0.18
178	mmu-miR-339-5p-4395368	Target	21.92	0.13	10.83	0.22
179	U87-4386735	Target	18.41	0.23	7.31	0.29
180	mmu-miR-340-3p-4395370	Target	21.42	0.28	10.33	0.33
181	mmu-miR-340-5p-4395369	Target	19.92	0.31	8.83	0.35
182	mmu-miR-342-3p-4395371	Target	21.12	0.22	10.02	0.28
183	mmu-miR-342-5p-4395657	Target	NA	NA	NA	NA
184	rno-miR-343-4381123	Target	NA	NA	NA	NA
185	mmu-miR-344-4373340	Target	29.88	0.06	18.78	0.18
186	rno-miR-344-3p-4381112	Target	NA	NA	NA	NA
187	rno-miR-344-5p-4395761	Target	37.24	0.03	26.15	0.18
188	mmu-miR-345-3p-4395659	Target	26.75	0.16	15.65	0.23
189	rno-miR-345-3p-4395762	Target	25.67	0.25	14.58	0.31
190	mmu-miR-345-5p-4395658	Target	22.91	0.18	11.82	0.25
191	mmu-miR-346-4373342	Target	NA	NA	NA	NA
192	rno-miR-346-4381113	Target	NA	NA	NA	NA
193	rno-miR-347-4381114	Target	NA	NA	NA	NA
194	rno-miR-349-4381115	Target	NA	NA	NA	NA
195	mmu-miR-350-4395660	Target	22.22	0.26	11.13	0.31
196	rno-miR-351-4395764	Target	20.68	0.23	9.59	0.29
197	mmu-miR-361-4373035	Target	18.90	0.35	7.81	0.39
198	mmu-miR-362-3p-4395746	Target	18.02	0.30	6.93	0.35
199	mmu-miR-363-4378090	Target	34.75	0.05	23.66	0.18
200	mmu-miR-365-4373194	Target	18.07	0.25	6.98	0.30
201	mmu-miR-367-4373034	Target	25.65	0.23	14.56	0.29
202	mmu-miR-369-3p-4373032	Target	17.00	0.40	5.91	0.44
203	mmu-miR-369-5p-4373195	Target	23.96	0.28	12.87	0.33
204	mmu-miR-370-4395386	Target	17.32	0.27	6.22	0.32
205	mmu-miR-375-4373027	Target	18.69	0.33	7.60	0.37
206	mmu-miR-376a-4373347	Target	18.55	0.27	7.46	0.32
207	mmu-miR-376b-4395582	Target	20.69	0.17	9.60	0.24
208	mmu-miR-376c-4395580	Target	14.84	0.30	3.75	0.35
209	mmu-miR-377-4373025	Target	25.71	0.14	14.62	0.22
210	rno-miR-377-4381100	Target	34.86	0.04	23.77	0.18
211	mmu-miR-379-4373349	Target	16.55	0.28	5.46	0.33
212	mmu-miR-380-3p-4373350	Target	25.55	0.18	14.46	0.25
213	mmu-miR-380-5p-4395731	Target	18.49	0.32	7.39	0.36
214	mmu-miR-381-4373020	Target	19.74	0.37	8.65	0.41
215	mmu-miR-382-4373019	Target	13.84	0.25	2.74	0.31
216	mmu-miR-383-4381093	Target	25.99	0.24	14.90	0.30
217	mmu-miR-384-3p-4395733	Target	25.04	0.23	13.94	0.29
218	mmu-miR-384-5p-4395732	Target	17.36	0.39	6.27	0.43
219	mmu-miR-409-3p-4395443	Target	15.88	0.28	4.79	0.33
220	mmu-miR-409-5p-4395442	Target	20.46	0.21	9.37	0.27
221	mmu-miR-410-4378093	Target	13.86	0.31	2.77	0.35
222	mmu-miR-411-4381013	Target	12.36	0.35	1.27	0.39
223	rno-miR-421-4381122	Target	NA	NA	NA	NA
224	mmu-miR-423-5p-4395451	Target	24.73	0.12	13.64	0.21
225	mmu-miR-425-4380926	Target	29.00	0.04	17.91	0.18
226	mmu-miR-429-4373355	Target	20.00	0.17	8.90	0.24
227	Y1-4386739	Target	17.91	0.25	6.81	0.31
228	mmu-miR-431-4395173	Target	17.23	0.30	6.14	0.35
229	mmu-miR-433-4373205	Target	19.32	0.24	8.23	0.30
230	mmu-miR-434-3p-4395734	Target	12.76	0.36	1.67	0.40
231	mmu-miR-434-5p-4395711	Target	20.80	0.25	9.71	0.30
232	mmu-miR-448-4373206	Target	29.74	0.11	18.65	0.21

233	mmu-miR-449a-4373207	Target	26.11	0.32	15.02	0.37
234	mmu-miR-449b-4395669	Target	NA	NA	NA	NA
235	mmu-miR-450a-5p-4395414	Target	21.60	0.33	10.50	0.37
236	mmu-miR-450b-5p-4386779	Target	32.01	0.04	20.92	0.18
237	mmu-miR-451-4373360	Target	18.99	0.32	7.90	0.37
238	mmu-miR-453-4395614	Target	32.48	0.04	21.39	0.18
239	mmu-miR-455-4395585	Target	18.99	0.31	7.90	0.36
240	mmu-miR-465a-3p-4395574	Target	32.41	0.05	21.31	0.18
241	mmu-miR-465a-5p-4373363	Target	NA	NA	NA	NA
242	mmu-miR-465b-5p-4395615	Target	32.31	0.05	21.22	0.18
243	rno-miR-466b-4395767	Target	NA	NA	NA	NA
244	rno-miR-466c-4395768	Target	NA	NA	NA	NA
245	mmu-miR-466h-4395646	Target	NA	NA	NA	NA
246	mmu-miR-467a-4395717	Target	19.66	0.34	8.57	0.38
247	mmu-miR-467c-4395647	Target	24.49	0.18	13.40	0.25
248	mmu-miR-467d-4395648	Target	24.55	0.16	13.46	0.24
249	mmu-miR-467e-4395698	Target	25.20	0.20	14.10	0.26
250	mmu-miR-470-4395718	Target	NA	NA	NA	NA
251	mmu-miR-484-4381032	Target	14.61	0.33	3.52	0.37
252	mmu-miR-486-4378096	Target	NA	NA	NA	NA
253	mmu-miR-487b-4378102	Target	32.46	0.17	21.37	0.24
254	mmu-miR-489-4378114	Target	25.11	0.22	14.02	0.28
255	mmu-miR-491-4381053	Target	24.81	0.15	13.72	0.23
256	mmu-miR-493-4395649	Target	26.90	0.23	15.81	0.29
257	mmu-miR-494-4395476	Target	20.00	0.26	8.91	0.32
258	mmu-miR-495-4381078	Target	16.99	0.30	5.90	0.35
259	mmu-miR-497-4381046	Target	21.39	0.25	10.30	0.30
260	mmu-miR-499-4381047	Target	32.66	0.05	21.57	0.18
261	mmu-miR-500-4395736	Target	20.31	0.27	9.22	0.32
262	mmu-miR-501-3p-4381069	Target	19.40	0.17	8.31	0.24
263	mmu-miR-503-4395586	Target	17.65	0.36	6.56	0.40
264	mmu-miR-504-4395195	Target	31.41	0.04	20.32	0.18
265	mmu-miR-505-4381071	Target	NA	NA	NA	NA
266	mmu-miR-509-3p-4395651	Target	36.27	0.03	25.18	0.18
267	mmu-miR-509-5p-4395650	Target	NA	NA	NA	NA
268	mmu-miR-511-4395679	Target	36.98	0.03	25.88	0.18
269	mmu-miR-532-3p-4395466	Target	14.98	0.31	3.89	0.36
270	mmu-miR-532-5p-4380928	Target	19.13	0.37	8.04	0.41
271	rno-miR-532-5p-4395752	Target	25.28	0.08	14.19	0.19
272	mmu-miR-539-4378103	Target	28.32	0.24	17.23	0.29
273	mmu-miR-540-3p-4378119	Target	38.75	0.02	27.66	0.18
274	mmu-miR-540-5p-4395691	Target	27.12	0.12	16.03	0.21
275	ath-miR159a-4373390	Target	NA	NA	NA	NA
276	mmu-miR-542-3p-4378101	Target	18.98	0.27	7.89	0.32
277	mmu-miR-542-5p-4395693	Target	25.35	0.19	14.26	0.26
278	mmu-miR-543-4395487	Target	18.83	0.29	7.73	0.34
279	rno-miR-543-4395766	Target	28.48	0.05	17.39	0.18
280	mmu-miR-544-4395680	Target	19.58	0.26	8.49	0.31
281	mmu-miR-551b-4380945	Target	26.32	0.28	15.23	0.33
282	mmu-miR-574-3p-4395460	Target	21.25	0.29	10.16	0.34
283	mmu-miR-582-3p-4395697	Target	27.70	0.19	16.61	0.26
284	mmu-miR-582-5p-4395696	Target	26.14	0.15	15.05	0.23
285	mmu-miR-590-5p-4395176	Target	38.82	0.02	27.73	0.18
286	mmu-miR-598-4395606	Target	25.15	0.22	14.06	0.28
287	rno-miR-598-5p-4395754	Target	32.26	0.07	21.17	0.19
288	mmu-miR-615-3p-4386777	Target	NA	NA	NA	NA
289	mmu-miR-615-5p-4395464	Target	NA	NA	NA	NA
290	mmu-miR-654-3p-4395350	Target	37.39	0.03	26.30	0.18
291	mmu-miR-654-5p-4395652	Target	NA	NA	NA	NA

292	mmu-miR-665-4395737	Target	24.38	0.25	13.29	0.31
293	mmu-miR-666-5p-4386770	Target	26.54	0.09	15.45	0.20
294	mmu-miR-669a-4381091	Target	23.84	0.21	12.74	0.27
295	mmu-miR-671-3p-4395433	Target	22.57	0.33	11.48	0.37
296	mmu-miR-672-4395438	Target	19.32	0.25	8.23	0.31
297	rno-miR-673-4395755	Target	28.47	0.16	17.38	0.24
298	mmu-miR-674-4395193	Target	23.54	0.14	12.45	0.23
299	mmu-miR-708-4395452	Target	18.80	0.31	7.71	0.35
300	mmu-miR-741-4395587	Target	29.32	0.08	18.23	0.19
301	mmu-miR-742-4395573	Target	NA	NA	NA	NA
302	rno-miR-742-4395756	Target	NA	NA	NA	NA
303	mmu-miR-743b-3p-4395601	Target	NA	NA	NA	NA
304	mmu-miR-743b-5p-4395600	Target	NA	NA	NA	NA
305	mmu-miR-743a-4395599	Target	NA	NA	NA	NA
306	rno-miR-743b-4395769	Target	NA	NA	NA	NA
307	mmu-miR-744-4395435	Target	18.57	0.33	7.48	0.37
308	rno-miR-758-4395180	Target	22.17	0.29	11.08	0.34
309	rno-miR-760-5p-4395758	Target	NA	NA	NA	NA
310	mmu-miR-770-3p-4395564	Target	31.42	0.04	20.33	0.18
311	mmu-miR-802-4395566	Target	33.48	0.05	22.39	0.18
312	mmu-miR-871-4395465	Target	NA	NA	NA	NA
313	rno-miR-871-4395770	Target	NA	NA	NA	NA
314	mmu-miR-872-4395375	Target	21.82	0.31	10.73	0.36
315	mmu-miR-873-4395467	Target	31.23	0.11	20.14	0.21
316	mmu-miR-874-4395379	Target	NA	NA	NA	NA
317	mmu-miR-875-3p-4395677	Target	NA	NA	NA	NA
318	mmu-miR-876-3p-4395594	Target	NA	NA	NA	NA
319	mmu-miR-876-5p-4395593	Target	NA	NA	NA	NA
320	rno-miR-878-4395771	Target	NA	NA	NA	NA
321	mmu-miR-878-5p-4395670	Target	NA	NA	NA	NA
322	mmu-miR-879-4395602	Target	31.57	0.10	20.48	0.20
323	mmu-miR-881-4395739	Target	NA	NA	NA	NA
324	rno-miR-881-4395773	Target	NA	NA	NA	NA
325	mmu-miR-883a-3p-4395591	Target	NA	NA	NA	NA
326	mmu-miR-883b-3p-4395695	Target	NA	NA	NA	NA
327	mmu-miR-883a-5p-4395741	Target	NA	NA	NA	NA
328	mmu-miR-125b-3p-4395489	Target	25.55	0.14	14.46	0.22
329	mmu-miR-143-4395360	Target	18.72	0.32	7.63	0.36
330	rno-miR-219-1-3p-4395778	Target	36.40	0.03	25.31	0.18
331	rno-miR-219-2-3p-4395501	Target	NA	NA	NA	NA
332	rno-miR-224-4373187	Target	18.39	0.21	7.30	0.27
333	mmu-miR-324-3p-4395639	Target	19.51	0.21	8.42	0.28
334	mmu-miR-351-4373345	Target	21.04	0.18	9.95	0.25
335	rno-miR-381-4381102	Target	22.22	0.30	11.13	0.34
336	rno-miR-409-5p-4381098	Target	NA	NA	NA	NA
337	rno-miR-450a-4381124	Target	25.15	0.17	14.06	0.24
338	mmu-miR-452-4373281	Target	NA	NA	NA	NA
339	mmu-miR-464-4373362	Target	NA	NA	NA	NA
340	mmu-miR-467b-4381084	Target	25.12	0.17	14.03	0.24
341	mmu-miR-468-4373366	Target	NA	NA	NA	NA
342	mmu-miR-469-4373367	Target	NA	NA	NA	NA
343	mmu-miR-488-4381074	Target	24.33	0.17	13.24	0.24
344	mmu-miR-490-4373215	Target	NA	NA	NA	NA
345	mmu-miR-496-4386771	Target	23.65	0.15	12.56	0.23
346	rno-miR-505-4381097	Target	32.22	0.06	21.12	0.18
347	mmu-miR-546-4381044	Target	39.71	0.02	28.62	0.18
348	mmu-miR-547-4395694	Target	31.11	0.05	20.02	0.18
349	mmu-miR-652-4395463	Target	15.71	0.28	4.61	0.33
350	mmu-miR-667-4386769	Target	20.81	0.23	9.71	0.29

351	mmu-miR-668-4386767	Target	23.49	0.19	12.40	0.26
352	mmu-miR-670-4395561	Target	NA	NA	NA	NA
353	mmu-miR-675-3p-4386762	Target	20.45	0.20	9.36	0.26
354	mmu-miR-675-5p-4386761	Target	NA	NA	NA	NA
355	mmu-miR-676-4386776	Target	19.14	0.24	8.05	0.30
356	mmu-miR-677-4381075	Target	29.29	0.20	18.20	0.26
357	mmu-miR-679-4381077	Target	26.59	0.13	15.50	0.22
358	mmu-miR-680-4381079	Target	NA	NA	NA	NA
359	mmu-miR-682-4381081	Target	27.78	0.06	16.69	0.19
360	mmu-miR-683-4381082	Target	NA	NA	NA	NA
361	mmu-miR-684-4381083	Target	30.96	0.09	19.87	0.20
362	mmu-miR-685-4386748	Target	25.23	0.07	14.14	0.19
363	mmu-miR-686-4381085	Target	NA	NA	NA	NA
364	mmu-miR-687-4386750	Target	NA	NA	NA	NA
365	mmu-let-7a-4373169	Target	17.59	0.16	6.50	0.24
366	mmu-let-7f-4373164	Target	20.31	0.13	9.22	0.22
367	mmu-miR-106b-4373155	Target	14.06	0.29	2.97	0.34
368	mmu-miR-155-4395701	Target	23.96	0.32	12.87	0.37
369	mmu-miR-17-4395419	Target	13.88	0.28	2.79	0.33
370	mmu-miR-23a-4373074	Target	NA	NA	NA	NA
371	mmu-miR-29a-4395223	Target	21.57	0.27	10.48	0.32
372	mmu-miR-30a-4373061	Target	15.17	0.28	4.08	0.33
373	mmu-miR-30e-4395334	Target	18.22	0.34	7.13	0.38
374	mmu-miR-31-4373331	Target	17.24	0.25	6.15	0.31
375	mmu-miR-34b-3p-4395748	Target	20.97	0.30	9.88	0.35
376	mmu-miR-92a-4373013	Target	12.22	0.40	1.13	0.44
377	rno-miR-190b-4395749	Target	23.70	0.29	12.61	0.34
378	mmu-miR-129-5p-4373171	Target	NA	NA	NA	NA
379	mmu-miR-187-4373307	Target	39.78	0.02	28.69	0.18
380	mmu-miR-188-3p-4395217	Target	35.04	0.03	23.95	0.18
381	mmu-miR-106a-4395589	Target	13.91	0.30	2.82	0.35
382	mmu-miR-188-5p-4395431	Target	21.93	0.29	10.83	0.34
383	mmu-miR-302b-4378071	Target	23.22	0.35	12.13	0.40
384	mmu-miR-322-4378107	Target	19.81	0.28	8.72	0.33

Supplementary Table 1.2.2 Heat Map for Submandibular Salivary Gland (SMG) miRNA-Signature

Relative abundance:

High Low

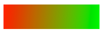

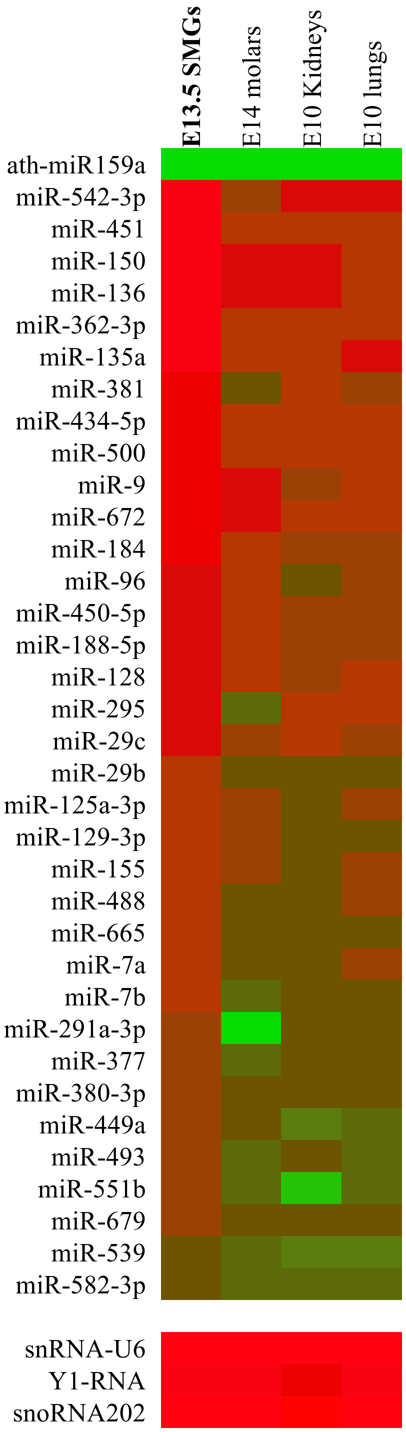

Supplementary Table 1.3.1 Raw CT data E12.5 Kidneys (NA = Not Available)

Well	miRNA ID	Task	CT AVG	ST DEV	Delta CT (Normalized to MammU6-4395470)	ST DEV
1	mmu-let-7b-4373168	Target	21.98	0.25	11.25	0.32
2	mmu-let-7c-4373167	Target	20.95	0.34	10.22	0.40
3	mmu-let-7d-4395394	Target	23.99	0.28	13.25	0.35
4	mmu-let-7e-4395517	Target	21.00	0.29	10.27	0.35
5	mmu-let-7g-4395393	Target	24.00	0.31	13.27	0.37
6	mmu-let-7i-4395332	Target	23.98	0.28	13.25	0.35
7	mmu-miR-1-4395333	Target	23.98	0.22	13.25	0.30
8	rno-miR-1-4395765	Target	24.00	0.36	13.27	0.41
9	mmu-miR-7a-4378130	Target	25.97	0.32	15.24	0.38
10	mmu-miR-7b-4395685	Target	27.97	0.32	17.24	0.38
11	MammU6-4395470	Control	10.73	0.21	0.00	0.29
12	MammU6-4395470	Control	10.67	0.20	-0.07	0.29
13	mmu-miR-9-4373285	Target	24.97	0.13	14.23	0.24
14	mmu-miR-10a-4373153	Target	20.97	0.26	10.24	0.33
15	mmu-miR-10b-4395329	Target	24.00	0.32	13.27	0.38
16	mmu-miR-15a-4373123	Target	21.99	0.13	11.26	0.24
17	mmu-miR-15b-4373122	Target	19.98	0.39	9.25	0.44
18	mmu-miR-16-4373121	Target	18.98	0.33	8.24	0.39
19	rno-miR-17-3p-4395779	Target	NA	NA	NA	NA
20	mmu-miR-18a-4395533	Target	17.96	0.31	7.23	0.37
21	mmu-miR-18b-4395596	Target	NA	NA	NA	NA
22	mmu-miR-19a-4373099	Target	16.99	0.29	6.25	0.35
23	mmu-miR-19b-4373098	Target	13.98	0.32	3.24	0.38
24	mmu-miR-20a-4373286	Target	12.99	0.34	2.26	0.40
25	mmu-miR-20b-4373263	Target	19.96	0.41	9.22	0.46
26	rno-miR-20b-3p-4395753	Target	20.59	0.01	9.85	0.21
27	mmu-miR-21-4373090	Target	20.98	0.25	10.24	0.32
28	mmu-miR-23b-4373073	Target	21.97	0.12	11.24	0.24
29	mmu-miR-24-4373072	Target	15.99	0.29	5.26	0.36
30	mmu-miR-25-4373071	Target	17.98	0.44	7.25	0.48
31	mmu-miR-26a-4395166	Target	15.98	0.27	5.25	0.34
32	mmu-miR-26b-4395167	Target	15.97	0.32	5.23	0.38
33	mmu-miR-27a-4373287	Target	20.98	0.38	10.25	0.43
34	mmu-miR-27b-4373068	Target	19.95	0.45	9.22	0.49
35	MammU6-4395470	Control	10.96	0.20	0.23	0.29
36	MammU6-4395470	Control	10.58	0.21	-0.15	0.30
37	mmu-miR-28-4373067	Target	17.97	0.30	7.24	0.36
38	mmu-miR-29b-4373288	Target	26.00	0.29	15.26	0.36
39	mmu-miR-29c-4395171	Target	23.99	0.43	13.25	0.48
40	mmu-miR-30b-4373290	Target	15.00	0.36	4.26	0.42
41	mmu-miR-30c-4373060	Target	13.95	0.31	3.22	0.37
42	mmu-miR-30d-4373059	Target	19.96	0.29	9.23	0.36
43	mmu-miR-32-4395220	Target	25.99	0.31	15.26	0.37
44	mmu-miR-34a-4395168	Target	21.00	0.11	10.27	0.23
45	mmu-miR-34c-4373036	Target	22.95	0.29	12.22	0.36
46	mmu-miR-93-4373302	Target	19.98	0.36	9.25	0.42
47	mmu-miR-96-4373372	Target	26.97	0.29	16.24	0.35
48	mmu-miR-98-4373009	Target	25.25	0.02	14.52	0.21
49	mmu-miR-99a-4373008	Target	20.01	0.18	9.27	0.27
50	mmu-miR-99b-4373007	Target	14.08	0.15	3.35	0.25
51	mmu-miR-100-4373160	Target	19.97	0.21	9.24	0.30
52	mmu-miR-101a-4395364	Target	19.94	0.39	9.21	0.44
53	mmu-miR-103-4373158	Target	19.99	0.20	9.26	0.29
54	mmu-miR-105-4395595	Target	NA	NA	NA	NA
55	mmu-miR-107-4373154	Target	24.97	0.20	14.23	0.28

56	mmu-miR-122-4395356	Target	15.95	0.32	5.21	0.38
57	mmu-miR-124-4373295	Target	23.04	0.02	12.31	0.21
58	mmu-miR-125a-3p-4395310	Target	25.97	0.20	15.24	0.29
59	mmu-miR-125a-5p-4395309	Target	18.98	0.33	8.25	0.39
60	mmu-miR-125b-5p-4373148	Target	14.97	0.35	4.23	0.40
61	mmu-miR-126-3p-4395339	Target	16.97	0.29	6.23	0.35
62	mmu-miR-126-5p-4373269	Target	16.99	0.34	6.25	0.40
63	mmu-miR-127-4373147	Target	13.96	0.40	3.22	0.45
64	mmu-miR-128a-4395327	Target	24.96	0.24	14.22	0.31
65	mmu-miR-129-3p-4373297	Target	26.00	0.10	15.27	0.23
66	mmu-miR-130a-4373145	Target	16.97	0.36	6.24	0.41
67	mmu-miR-130b-4373144	Target	17.98	0.28	7.25	0.35
68	mmu-miR-132-4373143	Target	20.06	0.12	9.32	0.24
69	mmu-miR-133a-4395357	Target	18.00	0.22	7.26	0.30
70	mmu-miR-133b-4395358	Target	24.04	0.11	13.30	0.23
71	mmu-miR-134-4373299	Target	20.00	0.20	9.27	0.28
72	mmu-miR-135a-4373140	Target	22.97	0.28	12.23	0.35
73	mmu-miR-135b-4395372	Target	23.98	0.28	13.25	0.35
74	mmu-miR-136-4395641	Target	21.98	0.36	11.25	0.42
75	mmu-miR-137-4373301	Target	29.01	0.15	18.28	0.25
76	mmu-miR-138-4395395	Target	20.94	0.40	10.20	0.45
77	mmu-miR-139-3p-4395676	Target	NA	NA	NA	NA
78	mmu-miR-139-5p-4395400	Target	18.99	0.28	8.25	0.35
79	mmu-miR-140-4373374	Target	21.97	0.30	11.23	0.37
80	mmu-miR-141-4373137	Target	24.02	0.15	13.28	0.25
81	mmu-miR-142-3p-4373136	Target	17.99	0.33	7.25	0.38
82	mmu-miR-142-5p-4395359	Target	25.98	0.22	15.24	0.30
83	snoRNA135-4380912	Target	18.01	0.25	7.27	0.33
84	mmu-miR-145-4395389	Target	16.96	0.28	6.23	0.35
85	mmu-miR-146a-4373132	Target	22.98	0.30	12.25	0.37
86	mmu-miR-146b-4373178	Target	23.99	0.23	13.25	0.31
87	mmu-miR-147-4395373	Target	NA	NA	NA	NA
88	mmu-miR-148a-4373130	Target	18.93	0.27	8.20	0.34
89	mmu-miR-148b-4373129	Target	22.98	0.20	12.24	0.29
90	mmu-miR-150-4373127	Target	20.98	0.38	10.24	0.43
91	mmu-miR-151-3p-4373304	Target	23.95	0.11	13.21	0.23
92	mmu-miR-152-4395170	Target	17.96	0.45	7.23	0.50
93	mmu-miR-153-4373305	Target	NA	NA	NA	NA
94	mmu-miR-154-4373270	Target	22.01	0.13	11.28	0.24
95	mmu-miR-181a-4373117	Target	17.00	0.17	6.27	0.27
96	mmu-miR-181c-4373115	Target	19.94	0.11	9.21	0.23
97	mmu-miR-182-4395729	Target	19.99	0.34	9.26	0.40
98	mmu-miR-183-4395380	Target	24.98	0.09	14.25	0.23
99	mmu-miR-184-4373113	Target	24.96	0.33	14.23	0.39
100	mmu-miR-185-4395382	Target	23.98	0.15	13.24	0.26
101	mmu-miR-186-4395396	Target	21.96	0.34	11.22	0.40
102	mmu-miR-190-4373110	Target	21.95	0.31	11.22	0.37
103	mmu-miR-191-4395410	Target	16.95	0.39	6.22	0.44
104	mmu-miR-192-4373108	Target	17.97	0.38	7.23	0.43
105	mmu-miR-193-4395361	Target	29.00	0.04	18.27	0.21
106	mmu-miR-193b-4395597	Target	18.00	0.33	7.26	0.39
107	mmu-miR-194-4373106	Target	22.00	0.32	11.26	0.38
108	mmu-miR-195-4373105	Target	22.95	0.31	12.22	0.37
109	mmu-miR-196b-4395326	Target	27.95	0.13	17.22	0.24
110	rno-miR-196c-4395750	Target	23.98	0.19	13.25	0.28
111	mmu-miR-197-4373102	Target	22.10	0.04	11.37	0.21
112	mmu-miR-199a-3p-4395415	Target	19.96	0.30	9.23	0.36
113	mmu-miR-199a-5p-4373272	Target	21.02	0.12	10.28	0.24
114	mmu-miR-200a-4378069	Target	19.94	0.13	9.21	0.24

115	mmu-miR-200b-4395362	Target	21.96	0.15	11.22	0.25
116	mmu-miR-200c-4395411	Target	22.01	0.18	11.28	0.27
117	mmu-miR-201-4395708	Target	NA	NA	NA	NA
118	mmu-miR-202-3p-4373311	Target	25.99	0.05	15.26	0.21
119	mmu-miR-202-5p-4395709	Target	32.03	0.07	21.29	0.22
120	mmu-miR-203-4373095	Target	20.97	0.32	10.24	0.38
121	mmu-miR-204-4373094	Target	23.00	0.14	12.27	0.25
122	mmu-miR-205-4373093	Target	24.96	0.24	14.23	0.32
123	mmu-miR-207-4373314	Target	26.15	0.01	15.42	0.21
124	rno-miR-207-4381096	Target	22.00	0.04	11.27	0.21
125	mmu-miR-208-4373091	Target	22.93	0.03	12.20	0.21
126	mmu-miR-208b-4395401	Target	27.92	0.04	17.18	0.21
127	mmu-miR-210-4373089	Target	18.95	0.13	8.21	0.24
128	mmu-miR-211-4373315	Target	NA	NA	NA	NA
129	mmu-miR-214-4395417	Target	18.98	0.23	8.25	0.31
130	mmu-miR-215-4373316	Target	29.98	0.16	19.25	0.26
131	snoRNA202-4380914	Target	14.97	0.34	4.24	0.40
132	mmu-miR-216a-4395331	Target	NA	NA	NA	NA
133	mmu-miR-216b-4395437	Target	23.96	0.20	13.23	0.29
134	mmu-miR-217-4395686	Target	29.01	0.06	18.28	0.22
135	mmu-miR-218-4373081	Target	15.96	0.37	5.23	0.42
136	mmu-miR-219-4373080	Target	25.86	0.01	15.13	0.21
137	mmu-miR-220-4395598	Target	NA	NA	NA	NA
138	mmu-miR-221-4373077	Target	20.99	0.27	10.26	0.34
139	mmu-miR-222-4395387	Target	22.95	0.33	12.21	0.39
140	mmu-miR-223-4395406	Target	18.97	0.32	8.24	0.38
141	mmu-miR-224-4395683	Target	22.97	0.32	12.24	0.38
142	mmu-miR-290-3p-4395721	Target	26.08	0.06	15.35	0.22
143	mmu-miR-291a-3p-4395722	Target	26.98	0.34	16.24	0.40
144	mmu-miR-291b-5p-4395667	Target	NA	NA	NA	NA
145	mmu-miR-292-3p-4395723	Target	19.95	0.46	9.21	0.50
146	mmu-miR-293-4386754	Target	23.97	0.32	13.23	0.38
147	mmu-miR-294-4373326	Target	25.98	0.28	15.25	0.35
148	mmu-miR-295-4373327	Target	22.97	0.34	12.24	0.40
149	mmu-miR-296-3p-4395212	Target	25.04	0.10	14.30	0.23
150	mmu-miR-296-5p-4373066	Target	15.96	0.19	5.22	0.28
151	mmu-miR-297c-4395610	Target	22.91	0.02	12.18	0.21
152	mmu-miR-297b-5p-4381049	Target	30.37	0.02	19.64	0.21
153	mmu-miR-298-4395728	Target	23.97	0.23	13.24	0.31
154	mmu-miR-301a-4373064	Target	19.98	0.25	9.25	0.32
155	mmu-miR-301b-4395730	Target	17.97	0.29	7.24	0.36
156	mmu-miR-302a-4378070	Target	20.99	0.25	10.26	0.33
157	mmu-miR-302c-4395688	Target	23.93	0.08	13.20	0.22
158	mmu-miR-302d-4373063	Target	21.95	0.29	11.22	0.36
159	mmu-miR-320-4395388	Target	21.97	0.27	11.24	0.34
160	mmu-miR-323-3p-4395338	Target	24.98	0.17	14.25	0.27
161	mmu-miR-324-5p-4373052	Target	17.94	0.27	7.21	0.34
162	mmu-miR-325-4395640	Target	24.99	0.05	14.25	0.21
163	rno-miR-327-4381108	Target	28.18	0.03	17.44	0.21
164	mmu-miR-328-4373049	Target	20.00	0.24	9.26	0.31
165	mmu-miR-329-4373336	Target	20.98	0.32	10.25	0.38
166	mmu-miR-330-4395341	Target	27.03	0.05	16.30	0.21
167	mmu-miR-331-3p-4373046	Target	17.97	0.28	7.24	0.34
168	mmu-miR-331-5p-4395344	Target	26.02	0.10	15.28	0.23
169	rno-miR-333-4381109	Target	30.99	0.02	20.25	0.21
170	mmu-miR-335-3p-4395296	Target	19.00	0.37	8.27	0.43
171	mmu-miR-335-5p-4373045	Target	15.97	0.27	5.24	0.34
172	rno-miR-336-4381111	Target	NA	NA	NA	NA
173	mmu-miR-337-3p-4395662	Target	19.97	0.35	9.24	0.41

174	mmu-miR-337-5p-4395645	Target	18.99	0.31	8.26	0.37
175	mmu-miR-338-3p-4395363	Target	31.08	0.03	20.34	0.21
176	mmu-miR-339-3p-4395663	Target	22.96	0.04	12.22	0.21
177	rno-miR-339-3p-4395760	Target	20.19	0.04	9.46	0.21
178	mmu-miR-339-5p-4395368	Target	21.07	0.13	10.34	0.24
179	U87-4386735	Target	18.97	0.25	8.24	0.32
180	mmu-miR-340-3p-4395370	Target	23.98	0.32	13.25	0.38
181	mmu-miR-340-5p-4395369	Target	22.99	0.29	12.26	0.36
182	mmu-miR-342-3p-4395371	Target	21.98	0.23	11.25	0.31
183	mmu-miR-342-5p-4395657	Target	20.81	0.03	10.07	0.21
184	rno-miR-343-4381123	Target	NA	NA	NA	NA
185	mmu-miR-344-4373340	Target	25.93	0.04	15.19	0.21
186	rno-miR-344-3p-4381112	Target	NA	NA	NA	NA
187	rno-miR-344-5p-4395761	Target	29.16	0.02	18.42	0.21
188	mmu-miR-345-3p-4395659	Target	26.95	0.14	16.22	0.25
189	rno-miR-345-3p-4395762	Target	27.95	0.30	17.22	0.37
190	mmu-miR-345-5p-4395658	Target	24.01	0.13	13.28	0.24
191	mmu-miR-346-4373342	Target	NA	NA	NA	NA
192	rno-miR-346-4381113	Target	25.91	0.01	15.18	0.21
193	rno-miR-347-4381114	Target	21.55	0.01	10.82	0.21
194	rno-miR-349-4381115	Target	25.87	0.02	15.14	0.21
195	mmu-miR-350-4395660	Target	23.97	0.27	13.24	0.34
196	rno-miR-351-4395764	Target	24.00	0.22	13.26	0.30
197	mmu-miR-361-4373035	Target	21.98	0.35	11.24	0.41
198	mmu-miR-362-3p-4395746	Target	22.94	0.33	12.21	0.39
199	mmu-miR-363-4378090	Target	31.13	0.04	20.40	0.21
200	mmu-miR-365-4373194	Target	19.96	0.23	9.23	0.31
201	mmu-miR-367-4373034	Target	20.96	0.32	10.23	0.38
202	mmu-miR-369-3p-4373032	Target	19.98	0.44	9.25	0.49
203	mmu-miR-369-5p-4373195	Target	26.99	0.31	16.26	0.37
204	mmu-miR-370-4395386	Target	18.96	0.28	8.22	0.35
205	mmu-miR-375-4373027	Target	20.98	0.39	10.25	0.44
206	mmu-miR-376a-4373347	Target	20.99	0.28	10.25	0.35
207	mmu-miR-376b-4395582	Target	20.00	0.18	9.27	0.27
208	mmu-miR-376c-4395580	Target	17.98	0.37	7.25	0.42
209	mmu-miR-377-4373025	Target	25.96	0.12	15.22	0.24
210	rno-miR-377-4381100	Target	29.13	0.03	18.40	0.21
211	mmu-miR-379-4373349	Target	19.97	0.31	9.23	0.37
212	mmu-miR-380-3p-4373350	Target	26.97	0.13	16.24	0.24
213	mmu-miR-380-5p-4395731	Target	19.98	0.29	9.25	0.35
214	mmu-miR-381-4373020	Target	22.97	0.33	12.23	0.39
215	mmu-miR-382-4373019	Target	15.97	0.26	5.24	0.33
216	mmu-miR-383-4381093	Target	30.02	0.09	19.28	0.22
217	mmu-miR-384-3p-4395733	Target	27.00	0.16	16.27	0.26
218	mmu-miR-384-5p-4395732	Target	21.96	0.43	11.23	0.47
219	mmu-miR-409-3p-4395443	Target	16.96	0.30	6.22	0.36
220	mmu-miR-409-5p-4395442	Target	21.99	0.18	11.26	0.27
221	mmu-miR-410-4378093	Target	15.98	0.33	5.25	0.39
222	mmu-miR-411-4381013	Target	14.96	0.43	4.23	0.47
223	rno-miR-421-4381122	Target	36.03	0.05	25.29	0.21
224	mmu-miR-423-5p-4395451	Target	23.02	0.09	12.29	0.23
225	mmu-miR-425-4380926	Target	18.30	0.03	7.57	0.21
226	mmu-miR-429-4373355	Target	21.96	0.09	11.23	0.23
227	Y1-4386739	Target	19.00	0.26	8.26	0.33
228	mmu-miR-431-4395173	Target	20.01	0.29	9.27	0.36
229	mmu-miR-433-4373205	Target	20.98	0.28	10.24	0.35
230	mmu-miR-434-3p-4395734	Target	15.99	0.37	5.25	0.42
231	mmu-miR-434-5p-4395711	Target	22.99	0.27	12.25	0.34
232	mmu-miR-448-4373206	Target	NA	NA	NA	NA

233	mmu-miR-449a-4373207	Target	31.00	0.20	20.26	0.29
234	mmu-miR-449b-4395669	Target	32.46	0.00	21.73	0.21
235	mmu-miR-450a-5p-4395414	Target	24.96	0.31	14.22	0.37
236	mmu-miR-450b-5p-4386779	Target	27.74	0.01	17.00	0.21
237	mmu-miR-451-4373360	Target	23.99	0.35	13.26	0.41
238	mmu-miR-453-4395614	Target	24.90	0.01	14.16	0.21
239	mmu-miR-455-4395585	Target	20.97	0.27	10.24	0.34
240	mmu-miR-465a-3p-4395574	Target	27.96	0.08	17.22	0.22
241	mmu-miR-465a-5p-4373363	Target	32.92	0.00	22.19	0.21
242	mmu-miR-465b-5p-4395615	Target	28.05	0.04	17.32	0.21
243	rno-miR-466b-4395767	Target	5.59	0.01	-5.15	0.21
244	rno-miR-466c-4395768	Target	13.79	0.01	3.05	0.21
245	mmu-miR-466h-4395646	Target	NA	NA	NA	NA
246	mmu-miR-467a-4395717	Target	20.96	0.38	10.23	0.43
247	mmu-miR-467c-4395647	Target	23.93	0.23	13.20	0.31
248	mmu-miR-467d-4395648	Target	23.97	0.16	13.23	0.26
249	mmu-miR-467e-4395698	Target	26.00	0.17	15.27	0.27
250	mmu-miR-470-4395718	Target	NA	NA	NA	NA
251	mmu-miR-484-4381032	Target	16.96	0.35	6.23	0.40
252	mmu-miR-486-4378096	Target	NA	NA	NA	NA
253	mmu-miR-487b-4378102	Target	34.98	0.18	24.25	0.27
254	mmu-miR-489-4378114	Target	24.98	0.26	14.25	0.33
255	mmu-miR-491-4381053	Target	24.97	0.07	14.24	0.22
256	mmu-miR-493-4395649	Target	27.99	0.25	17.26	0.33
257	mmu-miR-494-4395476	Target	20.98	0.30	10.25	0.37
258	mmu-miR-495-4381078	Target	18.97	0.38	8.24	0.43
259	mmu-miR-497-4381046	Target	23.96	0.29	13.23	0.35
260	mmu-miR-499-4381047	Target	27.21	0.02	16.48	0.21
261	mmu-miR-500-4395736	Target	23.98	0.21	13.24	0.29
262	mmu-miR-501-3p-4381069	Target	19.99	0.08	9.26	0.22
263	mmu-miR-503-4395586	Target	20.97	0.28	10.24	0.35
264	mmu-miR-504-4395195	Target	26.93	0.02	16.20	0.21
265	mmu-miR-505-4381071	Target	NA	NA	NA	NA
266	mmu-miR-509-3p-4395651	Target	27.97	0.03	17.24	0.21
267	mmu-miR-509-5p-4395650	Target	NA	NA	NA	NA
268	mmu-miR-511-4395679	Target	26.08	0.04	15.34	0.21
269	mmu-miR-532-3p-4395466	Target	18.97	0.34	8.23	0.39
270	mmu-miR-532-5p-4380928	Target	23.96	0.42	13.23	0.47
271	rno-miR-532-5p-4395752	Target	26.00	0.06	15.26	0.21
272	mmu-miR-539-4378103	Target	30.99	0.23	20.26	0.31
273	mmu-miR-540-3p-4378119	Target	33.81	0.01	23.07	0.21
274	mmu-miR-540-5p-4395691	Target	27.03	0.12	16.30	0.24
275	ath-miR159a-4373390	Target	NA	NA	NA	NA
276	mmu-miR-542-3p-4378101	Target	22.00	0.29	11.27	0.36
277	mmu-miR-542-5p-4395693	Target	27.98	0.17	17.24	0.27
278	mmu-miR-543-4395487	Target	19.96	0.34	9.23	0.39
279	rno-miR-543-4395766	Target	22.95	0.02	12.21	0.21
280	mmu-miR-544-4395680	Target	21.96	0.26	11.22	0.33
281	mmu-miR-551b-4380945	Target	36.04	0.03	25.31	0.21
282	mmu-miR-574-3p-4395460	Target	24.98	0.27	14.24	0.34
283	mmu-miR-582-3p-4395697	Target	28.95	0.10	18.22	0.23
284	mmu-miR-582-5p-4395696	Target	26.04	0.09	15.31	0.22
285	mmu-miR-590-5p-4395176	Target	31.99	0.05	21.26	0.21
286	mmu-miR-598-4395606	Target	27.96	0.12	17.23	0.24
287	rno-miR-598-5p-4395754	Target	32.02	0.04	21.29	0.21
288	mmu-miR-615-3p-4386777	Target	22.02	0.02	11.29	0.21
289	mmu-miR-615-5p-4395464	Target	33.82	0.02	23.08	0.21
290	mmu-miR-654-3p-4395350	Target	30.00	0.07	19.26	0.22
291	mmu-miR-654-5p-4395652	Target	NA	NA	NA	NA

292	mmu-miR-665-4395737	Target	25.97	0.27	15.23	0.34
293	mmu-miR-666-5p-4386770	Target	25.01	0.11	14.28	0.23
294	mmu-miR-669a-4381091	Target	23.99	0.23	13.25	0.31
295	mmu-miR-671-3p-4395433	Target	24.97	0.31	14.24	0.37
296	mmu-miR-672-4395438	Target	23.06	0.08	12.33	0.22
297	rno-miR-673-4395755	Target	28.99	0.11	18.26	0.23
298	mmu-miR-674-4395193	Target	24.03	0.11	13.30	0.23
299	mmu-miR-708-4395452	Target	22.98	0.39	12.24	0.44
300	mmu-miR-741-4395587	Target	28.02	0.09	17.29	0.22
301	mmu-miR-742-4395573	Target	NA	NA	NA	NA
302	rno-miR-742-4395756	Target	NA	NA	NA	NA
303	mmu-miR-743b-3p-4395601	Target	NA	NA	NA	NA
304	mmu-miR-743b-5p-4395600	Target	22.74	0.01	12.01	0.21
305	mmu-miR-743a-4395599	Target	22.08	0.02	11.34	0.21
306	rno-miR-743b-4395769	Target	35.19	0.01	24.45	0.21
307	mmu-miR-744-4395435	Target	20.97	0.37	10.23	0.42
308	rno-miR-758-4395180	Target	22.99	0.39	12.25	0.45
309	rno-miR-760-5p-4395758	Target	NA	NA	NA	NA
310	mmu-miR-770-3p-4395564	Target	26.11	0.03	15.38	0.21
311	mmu-miR-802-4395566	Target	29.89	0.02	19.16	0.21
312	mmu-miR-871-4395465	Target	NA	NA	NA	NA
313	rno-miR-871-4395770	Target	NA	NA	NA	NA
314	mmu-miR-872-4395375	Target	24.96	0.33	14.23	0.39
315	mmu-miR-873-4395467	Target	33.02	0.09	22.28	0.23
316	mmu-miR-874-4395379	Target	NA	NA	NA	NA
317	mmu-miR-875-3p-4395677	Target	NA	NA	NA	NA
318	mmu-miR-876-3p-4395594	Target	NA	NA	NA	NA
319	mmu-miR-876-5p-4395593	Target	NA	NA	NA	NA
320	rno-miR-878-4395771	Target	28.88	0.01	18.15	0.21
321	mmu-miR-878-5p-4395670	Target	31.32	0.01	20.58	0.21
322	mmu-miR-879-4395602	Target	NA	NA	NA	NA
323	mmu-miR-881-4395739	Target	NA	NA	NA	NA
324	rno-miR-881-4395773	Target	NA	NA	NA	NA
325	mmu-miR-883a-3p-4395591	Target	NA	NA	NA	NA
326	mmu-miR-883b-3p-4395695	Target	NA	NA	NA	NA
327	mmu-miR-883a-5p-4395741	Target	NA	NA	NA	NA
328	mmu-miR-125b-3p-4395489	Target	26.98	0.13	16.24	0.24
329	mmu-miR-143-4395360	Target	20.97	0.36	10.24	0.42
330	rno-miR-219-1-3p-4395778	Target	24.98	0.03	14.25	0.21
331	rno-miR-219-2-3p-4395501	Target	NA	NA	NA	NA
332	rno-miR-224-4373187	Target	18.96	0.26	8.22	0.33
333	mmu-miR-324-3p-4395639	Target	20.00	0.21	9.27	0.29
334	mmu-miR-351-4373345	Target	22.97	0.13	12.23	0.24
335	rno-miR-381-4381102	Target	24.01	0.30	13.28	0.37
336	rno-miR-409-5p-4381098	Target	8.25	0.02	-2.48	0.21
337	rno-miR-450a-4381124	Target	27.02	0.11	16.29	0.23
338	mmu-miR-452-4373281	Target	NA	NA	NA	NA
339	mmu-miR-464-4373362	Target	NA	NA	NA	NA
340	mmu-miR-467b-4381084	Target	24.98	0.19	14.25	0.28
341	mmu-miR-468-4373366	Target	NA	NA	NA	NA
342	mmu-miR-469-4373367	Target	29.88	0.01	19.15	0.21
343	mmu-miR-488-4381074	Target	25.96	0.13	15.23	0.25
344	mmu-miR-490-4373215	Target	25.23	0.01	14.49	0.21
345	mmu-miR-496-4386771	Target	24.01	0.17	13.28	0.27
346	rno-miR-505-4381097	Target	30.08	0.02	19.35	0.21
347	mmu-miR-546-4381044	Target	32.88	0.01	22.14	0.21
348	mmu-miR-547-4395694	Target	26.00	0.05	15.26	0.21
349	mmu-miR-652-4395463	Target	18.99	0.34	8.26	0.40
350	mmu-miR-667-4386769	Target	21.96	0.27	11.23	0.34

351	mmu-miR-668-4386767	Target	24.98	0.13	14.24	0.24
352	mmu-miR-670-4395561	Target	NA	NA	NA	NA
353	mmu-miR-675-3p-4386762	Target	21.98	0.18	11.24	0.27
354	mmu-miR-675-5p-4386761	Target	NA	NA	NA	NA
355	mmu-miR-676-4386776	Target	23.03	0.17	12.29	0.26
356	mmu-miR-677-4381075	Target	29.99	0.19	19.25	0.28
357	mmu-miR-679-4381077	Target	25.99	0.21	15.26	0.29
358	mmu-miR-680-4381079	Target	24.82	0.01	14.08	0.21
359	mmu-miR-682-4381081	Target	21.98	0.09	11.25	0.22
360	mmu-miR-683-4381082	Target	NA	NA	NA	NA
361	mmu-miR-684-4381083	Target	28.94	0.14	18.20	0.25
362	mmu-miR-685-4386748	Target	20.03	0.07	9.30	0.22
363	mmu-miR-686-4381085	Target	NA	NA	NA	NA
364	mmu-miR-687-4386750	Target	NA	NA	NA	NA
365	mmu-let-7a-4373169	Target	19.99	0.07	9.26	0.22
366	mmu-let-7f-4373164	Target	20.88	0.06	10.14	0.21
367	mmu-miR-106b-4373155	Target	15.97	0.31	5.23	0.37
368	mmu-miR-155-4395701	Target	26.96	0.38	16.23	0.43
369	mmu-miR-17-4395419	Target	14.98	0.33	4.25	0.39
370	mmu-miR-23a-4373074	Target	NA	NA	NA	NA
371	mmu-miR-29a-4395223	Target	22.95	0.31	12.21	0.37
372	mmu-miR-30a-4373061	Target	18.97	0.37	8.23	0.42
373	mmu-miR-30e-4395334	Target	21.95	0.36	11.22	0.41
374	mmu-miR-31-4373331	Target	19.98	0.31	9.24	0.37
375	mmu-miR-34b-3p-4395748	Target	21.96	0.26	11.22	0.33
376	mmu-miR-92a-4373013	Target	13.97	0.49	3.23	0.53
377	rno-miR-190b-4395749	Target	25.95	0.31	15.22	0.37
378	mmu-miR-129-5p-4373171	Target	NA	NA	NA	NA
379	mmu-miR-187-4373307	Target	19.90	0.02	9.17	0.21
380	mmu-miR-188-3p-4395217	Target	NA	NA	NA	NA
381	mmu-miR-106a-4395589	Target	14.98	0.34	4.24	0.40
382	mmu-miR-188-5p-4395431	Target	24.96	0.29	14.23	0.36
383	mmu-miR-302b-4378071	Target	19.98	0.40	9.24	0.45
384	mmu-miR-322-4378107	Target	21.98	0.33	11.24	0.39

Supplementary Table 1.3.2 Heat Map for Kidney miRNA-Signature

Relative abundance:

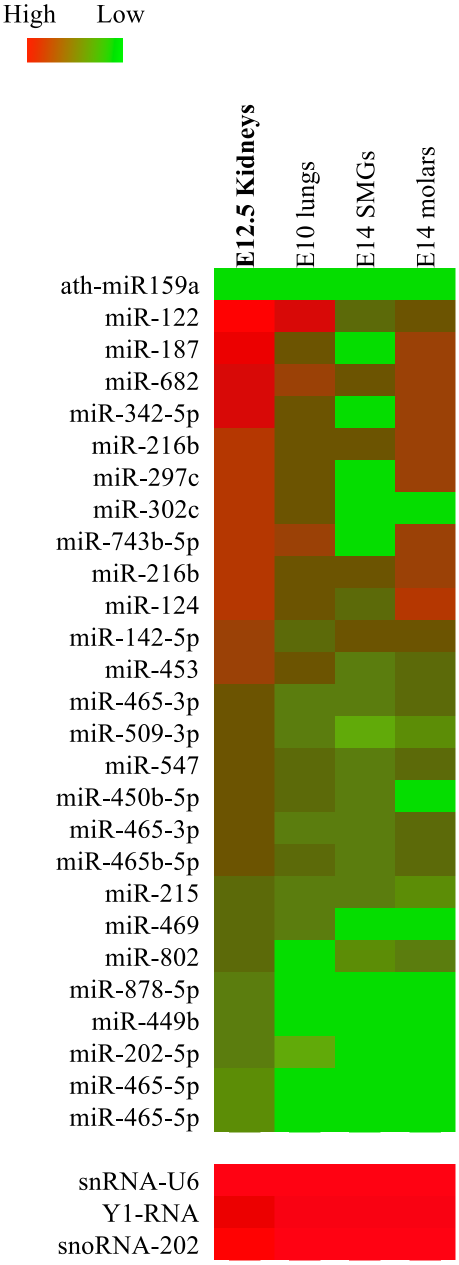

Supplementary Table 1.4.1 Raw CT data E11.5 Lungs (NA = Not Available)

Well	miRNA ID	Task	CT AVG	ST DEV	Delta CT (Normalized to MammU6-4395470)	ST DEV
1	mmu-let-7b-4373168	Target	20.96	3.07	11.02	3.57
2	mmu-let-7c-4373167	Target	19.96	3.40	10.02	3.86
3	mmu-let-7d-4395394	Target	23.98	2.83	14.04	3.36
4	mmu-let-7e-4395517	Target	19.97	3.28	10.03	3.75
5	mmu-let-7g-4395393	Target	23.95	3.10	14.01	3.60
6	mmu-let-7i-4395332	Target	22.97	2.70	13.03	3.26
7	mmu-miR-1-4395333	Target	21.98	2.31	12.04	2.94
8	rno-miR-1-4395765	Target	20.94	3.69	11.00	4.11
9	mmu-miR-7a-4378130	Target	24.96	3.06	15.02	3.56
10	mmu-miR-7b-4395685	Target	26.99	3.17	17.04	3.66
11	MammU6-4395470	Control	9.94	1.81	0.00	2.56
12	MammU6-4395470	Control	9.51	1.91	-0.43	2.63
13	mmu-miR-9-4373285	Target	22.97	2.16	13.03	2.82
14	mmu-miR-10a-4373153	Target	18.99	2.50	9.05	3.09
15	mmu-miR-10b-4395329	Target	22.97	2.77	13.02	3.31
16	mmu-miR-15a-4373123	Target	20.98	1.50	11.03	2.36
17	mmu-miR-15b-4373122	Target	17.98	3.45	8.03	3.89
18	mmu-miR-16-4373121	Target	16.95	3.41	7.01	3.86
19	rno-miR-17-3p-4395779	Target	NA	NA	NA	NA
20	mmu-miR-18a-4395533	Target	16.97	3.10	7.03	3.59
21	mmu-miR-18b-4395596	Target	24.91	0.28	14.97	1.83
22	mmu-miR-19a-4373099	Target	14.95	2.33	5.01	2.96
23	mmu-miR-19b-4373098	Target	11.98	2.11	2.04	2.78
24	mmu-miR-20a-4373286	Target	10.99	1.94	1.05	2.66
25	mmu-miR-20b-4373263	Target	17.95	4.57	8.01	4.92
26	rno-miR-20b-3p-4395753	Target	NA	NA	NA	NA
27	mmu-miR-21-4373090	Target	20.98	3.07	11.03	3.57
28	mmu-miR-23b-4373073	Target	20.97	2.87	11.03	3.39
29	mmu-miR-24-4373072	Target	12.98	2.97	3.03	3.48
30	mmu-miR-25-4373071	Target	15.97	4.30	6.03	4.67
31	mmu-miR-26a-4395166	Target	14.98	2.82	5.04	3.35
32	mmu-miR-26b-4395167	Target	14.97	2.82	5.03	3.35
33	mmu-miR-27a-4373287	Target	20.98	3.16	11.03	3.64
34	mmu-miR-27b-4373068	Target	16.94	4.16	7.00	4.54
35	MammU6-4395470	Control	9.26	2.30	-0.68	2.93
36	MammU6-4395470	Control	9.33	2.05	-0.61	2.74
37	mmu-miR-28-4373067	Target	15.96	2.78	6.02	3.32
38	mmu-miR-29b-4373288	Target	26.98	2.29	17.04	2.92
39	mmu-miR-29c-4395171	Target	23.98	3.81	14.04	4.22
40	mmu-miR-30b-4373290	Target	12.98	3.24	3.04	3.71
41	mmu-miR-30c-4373060	Target	11.96	2.57	2.02	3.14
42	mmu-miR-30d-4373059	Target	18.95	2.67	9.01	3.22
43	mmu-miR-32-4395220	Target	24.98	3.25	15.04	3.72
44	mmu-miR-34a-4395168	Target	20.97	1.82	11.02	2.57
45	mmu-miR-34c-4373036	Target	20.97	2.94	11.03	3.46
46	mmu-miR-93-4373302	Target	17.98	2.68	8.04	3.24
47	mmu-miR-96-4373372	Target	24.98	2.60	15.04	3.17
48	mmu-miR-98-4373009	Target	NA	NA	NA	NA
49	mmu-miR-99a-4373008	Target	18.01	2.18	8.07	2.84
50	mmu-miR-99b-4373007	Target	11.06	1.58	1.12	2.40
51	mmu-miR-100-4373160	Target	17.93	2.46	7.99	3.05
52	mmu-miR-101a-4395364	Target	19.99	4.05	10.05	4.44
53	mmu-miR-103-4373158	Target	17.97	2.08	8.03	2.76
54	mmu-miR-105-4395595	Target	NA	NA	NA	NA
55	mmu-miR-107-4373154	Target	23.98	2.19	14.04	2.84
56	mmu-miR-122-4395356	Target	20.98	2.63	11.04	3.19

57	mmu-miR-124-4373295	Target	26.05	0.32	16.11	1.84
58	mmu-miR-125a-3p-4395310	Target	24.01	2.27	14.07	2.91
59	mmu-miR-125a-5p-4395309	Target	15.95	3.40	6.01	3.86
60	mmu-miR-125b-5p-4373148	Target	12.99	3.36	3.05	3.82
61	mmu-miR-126-3p-4395339	Target	15.99	2.83	6.05	3.36
62	mmu-miR-126-5p-4373269	Target	15.97	3.00	6.03	3.51
63	mmu-miR-127-4373147	Target	14.98	3.93	5.04	4.33
64	mmu-miR-128a-4395327	Target	22.98	2.58	13.04	3.15
65	mmu-miR-129-3p-4373297	Target	25.98	1.71	16.04	2.49
66	mmu-miR-130a-4373145	Target	14.96	2.96	5.02	3.47
67	mmu-miR-130b-4373144	Target	15.99	2.57	6.04	3.14
68	mmu-miR-132-4373143	Target	18.98	2.97	9.04	3.48
69	mmu-miR-133a-4395357	Target	16.00	2.17	6.06	2.83
70	mmu-miR-133b-4395358	Target	22.00	0.94	12.06	2.04
71	mmu-miR-134-4373299	Target	19.96	1.38	10.02	2.28
72	mmu-miR-135a-4373140	Target	19.96	1.80	10.02	2.56
73	mmu-miR-135b-4395372	Target	20.98	3.40	11.04	3.86
74	mmu-miR-136-4395641	Target	22.98	3.83	13.03	4.24
75	mmu-miR-137-4373301	Target	24.95	3.58	15.01	4.01
76	mmu-miR-138-4395395	Target	18.98	4.33	9.04	4.70
77	mmu-miR-139-3p-4395676	Target	21.30	0.14	11.36	1.82
78	mmu-miR-139-5p-4395400	Target	17.96	2.87	8.02	3.39
79	mmu-miR-140-4373374	Target	20.97	3.31	11.03	3.78
80	mmu-miR-141-4373137	Target	21.99	1.86	12.05	2.60
81	mmu-miR-142-3p-4373136	Target	21.98	3.17	12.04	3.65
82	mmu-miR-142-5p-4395359	Target	28.98	1.16	19.04	2.15
83	snoRNA135-4380912	Target	15.97	2.50	6.03	3.09
84	mmu-miR-145-4395389	Target	14.95	2.88	5.00	3.40
85	mmu-miR-146a-4373132	Target	21.97	2.89	12.03	3.41
86	mmu-miR-146b-4373178	Target	22.98	2.54	13.04	3.12
87	mmu-miR-147-4395373	Target	25.98	0.22	16.04	1.83
88	mmu-miR-148a-4373130	Target	17.98	2.70	8.04	3.25
89	mmu-miR-148b-4373129	Target	21.97	1.97	12.02	2.68
90	mmu-miR-150-4373127	Target	21.96	2.98	12.02	3.49
91	mmu-miR-151-3p-4373304	Target	21.98	1.47	12.03	2.33
92	mmu-miR-152-4395170	Target	16.96	3.94	7.02	4.34
93	mmu-miR-153-4373305	Target	31.95	0.84	22.00	2.00
94	mmu-miR-154-4373270	Target	22.09	0.71	12.15	1.95
95	mmu-miR-181a-4373117	Target	15.01	1.02	5.07	2.08
96	mmu-miR-181c-4373115	Target	17.87	0.76	7.93	1.97
97	mmu-miR-182-4395729	Target	15.97	3.68	6.03	4.11
98	mmu-miR-183-4395380	Target	22.01	1.18	12.06	2.16
99	mmu-miR-184-4373113	Target	24.98	2.93	15.04	3.44
100	mmu-miR-185-4395382	Target	22.96	1.51	13.02	2.36
101	mmu-miR-186-4395396	Target	19.98	3.60	10.04	4.03
102	mmu-miR-190-4373110	Target	21.98	2.94	12.04	3.45
103	mmu-miR-191-4395410	Target	15.98	3.43	6.03	3.88
104	mmu-miR-192-4373108	Target	20.96	3.65	11.02	4.07
105	mmu-miR-193-4395361	Target	30.01	0.42	20.06	1.86
106	mmu-miR-193b-4395597	Target	18.95	2.70	9.01	3.25
107	mmu-miR-194-4373106	Target	23.98	2.39	14.04	3.00
108	mmu-miR-195-4373105	Target	21.98	2.62	12.04	3.19
109	mmu-miR-196b-4395326	Target	28.01	1.25	18.07	2.21
110	rno-miR-196c-4395750	Target	22.98	2.08	13.04	2.76
111	mmu-miR-197-4373102	Target	21.92	0.44	11.98	1.87
112	mmu-miR-199a-3p-4395415	Target	18.01	2.67	8.06	3.23
113	mmu-miR-199a-5p-4373272	Target	19.99	1.44	10.05	2.31
114	mmu-miR-200a-4378069	Target	17.98	2.37	8.04	2.99
115	mmu-miR-200b-4395362	Target	20.00	1.89	10.05	2.62

116	mmu-miR-200c-4395411	Target	19.00	2.17	9.06	2.83
117	mmu-miR-201-4395708	Target	NA	NA	NA	NA
118	mmu-miR-202-3p-4373311	Target	26.01	0.35	16.07	1.85
119	mmu-miR-202-5p-4395709	Target	34.05	0.21	24.11	1.83
120	mmu-miR-203-4373095	Target	17.01	2.12	7.07	2.79
121	mmu-miR-204-4373094	Target	20.98	2.93	11.04	3.45
122	mmu-miR-205-4373093	Target	19.00	3.12	9.06	3.61
123	mmu-miR-207-4373314	Target	25.05	0.14	15.11	1.82
124	rno-miR-207-4381096	Target	20.93	0.44	10.99	1.87
125	mmu-miR-208-4373091	Target	25.92	0.59	15.98	1.91
126	mmu-miR-208b-4395401	Target	29.95	0.33	20.01	1.84
127	mmu-miR-210-4373089	Target	18.02	1.36	8.08	2.27
128	mmu-miR-211-4373315	Target	22.54	0.07	12.60	1.82
129	mmu-miR-214-4395417	Target	16.97	2.77	7.03	3.31
130	mmu-miR-215-4373316	Target	30.97	1.63	21.03	2.44
131	snoRNA202-4380914	Target	12.96	3.34	3.02	3.80
132	mmu-miR-216a-4395331	Target	20.23	0.08	10.29	1.82
133	mmu-miR-216b-4395437	Target	26.00	1.31	16.06	2.24
134	mmu-miR-217-4395686	Target	29.11	0.36	19.17	1.85
135	mmu-miR-218-4373081	Target	11.95	3.85	2.01	4.26
136	mmu-miR-219-4373080	Target	26.99	0.49	17.05	1.88
137	mmu-miR-220-4395598	Target	NA	NA	NA	NA
138	mmu-miR-221-4373077	Target	19.98	2.66	10.04	3.22
139	mmu-miR-222-4395387	Target	21.95	3.12	12.01	3.61
140	mmu-miR-223-4395406	Target	22.99	2.71	13.05	3.26
141	mmu-miR-224-4395683	Target	21.97	3.21	12.03	3.69
142	mmu-miR-290-3p-4395721	Target	26.30	0.21	16.36	1.83
143	mmu-miR-291a-3p-4395722	Target	26.96	2.55	17.02	3.13
144	mmu-miR-291b-5p-4395667	Target	NA	NA	NA	NA
145	mmu-miR-292-3p-4395723	Target	19.96	4.72	10.02	5.05
146	mmu-miR-293-4386754	Target	23.96	3.28	14.02	3.75
147	mmu-miR-294-4373326	Target	24.96	3.11	15.01	3.60
148	mmu-miR-295-4373327	Target	21.94	4.03	12.00	4.42
149	mmu-miR-296-3p-4395212	Target	22.97	2.11	13.03	2.78
150	mmu-miR-296-5p-4373066	Target	15.01	1.88	5.07	2.61
151	mmu-miR-297c-4395610	Target	25.86	0.25	15.92	1.83
152	mmu-miR-297b-5p-4381049	Target	28.15	0.19	18.20	1.82
153	mmu-miR-298-4395728	Target	22.98	2.61	13.04	3.18
154	mmu-miR-301a-4373064	Target	16.98	2.59	7.04	3.16
155	mmu-miR-301b-4395730	Target	15.98	2.69	6.04	3.24
156	mmu-miR-302a-4378070	Target	18.97	2.75	9.03	3.30
157	mmu-miR-302c-4395688	Target	25.99	1.13	16.05	2.14
158	mmu-miR-302d-4373063	Target	20.97	2.59	11.02	3.16
159	mmu-miR-320-4395388	Target	19.96	2.62	10.02	3.19
160	mmu-miR-323-3p-4395338	Target	24.99	1.61	15.04	2.42
161	mmu-miR-324-5p-4373052	Target	16.98	2.54	7.04	3.12
162	mmu-miR-325-4395640	Target	22.99	2.03	13.04	2.72
163	rno-miR-327-4381108	Target	31.87	0.15	21.93	1.82
164	mmu-miR-328-4373049	Target	17.96	2.26	8.02	2.90
165	mmu-miR-329-4373336	Target	20.96	2.79	11.02	3.32
166	mmu-miR-330-4395341	Target	26.94	0.84	16.99	2.00
167	mmu-miR-331-3p-4373046	Target	16.00	2.25	6.06	2.89
168	mmu-miR-331-5p-4395344	Target	25.99	1.79	16.05	2.55
169	rno-miR-333-4381109	Target	20.78	0.08	10.84	1.82
170	mmu-miR-335-3p-4395296	Target	16.99	3.76	7.04	4.18
171	mmu-miR-335-5p-4373045	Target	14.94	2.98	5.00	3.49
172	rno-miR-336-4381111	Target	NA	NA	NA	NA
173	mmu-miR-337-3p-4395662	Target	20.98	3.15	11.04	3.63
174	mmu-miR-337-5p-4395645	Target	18.98	3.34	9.04	3.80

175	mmu-miR-338-3p-4395363	Target	28.98	1.94	19.04	2.66
176	mmu-miR-339-3p-4395663	Target	24.01	0.73	14.06	1.96
177	rno-miR-339-3p-4395760	Target	22.00	0.87	12.06	2.01
178	mmu-miR-339-5p-4395368	Target	19.99	1.37	10.05	2.27
179	U87-4386735	Target	16.98	2.70	7.04	3.25
180	mmu-miR-340-3p-4395370	Target	21.97	3.15	12.02	3.64
181	mmu-miR-340-5p-4395369	Target	20.99	3.25	11.04	3.72
182	mmu-miR-342-3p-4395371	Target	18.96	2.13	9.02	2.80
183	mmu-miR-342-5p-4395657	Target	26.06	0.68	16.12	1.94
184	rno-miR-343-4381123	Target	NA	NA	NA	NA
185	mmu-miR-344-4373340	Target	25.00	1.00	15.06	2.07
186	rno-miR-344-3p-4381112	Target	NA	NA	NA	NA
187	rno-miR-344-5p-4395761	Target	28.01	0.92	18.07	2.03
188	mmu-miR-345-3p-4395659	Target	25.95	1.71	16.01	2.49
189	rno-miR-345-3p-4395762	Target	26.99	2.84	17.05	3.37
190	mmu-miR-345-5p-4395658	Target	23.01	1.44	13.07	2.32
191	mmu-miR-346-4373342	Target	NA	NA	NA	NA
192	rno-miR-346-4381113	Target	NA	NA	NA	NA
193	rno-miR-347-4381114	Target	34.78	0.06	24.84	1.81
194	rno-miR-349-4381115	Target	32.08	0.07	22.14	1.81
195	mmu-miR-350-4395660	Target	22.96	2.78	13.02	3.32
196	rno-miR-351-4395764	Target	21.98	2.43	12.03	3.03
197	mmu-miR-361-4373035	Target	20.99	3.76	11.05	4.17
198	mmu-miR-362-3p-4395746	Target	22.98	3.20	13.04	3.68
199	mmu-miR-363-4378090	Target	31.05	1.18	21.10	2.17
200	mmu-miR-365-4373194	Target	20.98	2.29	11.04	2.92
201	mmu-miR-367-4373034	Target	19.96	3.06	10.02	3.56
202	mmu-miR-369-3p-4373032	Target	21.97	4.62	12.03	4.96
203	mmu-miR-369-5p-4373195	Target	26.97	3.22	17.02	3.70
204	mmu-miR-370-4395386	Target	18.96	2.86	9.02	3.39
205	mmu-miR-375-4373027	Target	18.97	3.85	9.03	4.26
206	mmu-miR-376a-4373347	Target	21.99	2.92	12.05	3.44
207	mmu-miR-376b-4395582	Target	22.02	1.33	12.08	2.25
208	mmu-miR-376c-4395580	Target	17.98	2.86	8.04	3.39
209	mmu-miR-377-4373025	Target	26.96	0.42	17.02	1.86
210	rno-miR-377-4381100	Target	30.05	0.08	20.10	1.82
211	mmu-miR-379-4373349	Target	19.97	3.01	10.03	3.52
212	mmu-miR-380-3p-4373350	Target	27.04	1.17	17.10	2.16
213	mmu-miR-380-5p-4395731	Target	19.98	2.69	10.03	3.24
214	mmu-miR-381-4373020	Target	23.97	3.05	14.02	3.55
215	mmu-miR-382-4373019	Target	16.00	1.71	6.05	2.49
216	mmu-miR-383-4381093	Target	27.98	1.75	18.04	2.52
217	mmu-miR-384-3p-4395733	Target	25.00	3.19	15.05	3.67
218	mmu-miR-384-5p-4395732	Target	17.95	3.82	8.01	4.23
219	mmu-miR-409-3p-4395443	Target	17.95	3.24	8.01	3.72
220	mmu-miR-409-5p-4395442	Target	22.01	1.60	12.07	2.42
221	mmu-miR-410-4378093	Target	15.97	3.27	6.03	3.74
222	mmu-miR-411-4381013	Target	15.98	4.11	6.04	4.50
223	rno-miR-421-4381122	Target	30.84	0.32	20.90	1.84
224	mmu-miR-423-5p-4395451	Target	22.99	1.45	13.05	2.32
225	mmu-miR-425-4380926	Target	NA	NA	NA	NA
226	mmu-miR-429-4373355	Target	20.00	1.53	10.06	2.37
227	Y1-4386739	Target	15.99	2.89	6.05	3.41
228	mmu-miR-431-4395173	Target	19.99	2.66	10.05	3.22
229	mmu-miR-433-4373205	Target	21.00	2.45	11.06	3.05
230	mmu-miR-434-3p-4395734	Target	15.98	3.38	6.04	3.84
231	mmu-miR-434-5p-4395711	Target	22.96	2.31	13.02	2.94
232	mmu-miR-448-4373206	Target	NA	NA	NA	NA
233	mmu-miR-449a-4373207	Target	27.95	2.91	18.01	3.43

234	mmu-miR-449b-4395669	Target	NA	NA	NA	NA
235	mmu-miR-450a-5p-4395414	Target	23.95	3.18	14.01	3.66
236	mmu-miR-450b-5p-4386779	Target	27.94	0.46	18.00	1.87
237	mmu-miR-451-4373360	Target	22.94	3.46	13.00	3.90
238	mmu-miR-453-4395614	Target	25.89	0.25	15.95	1.83
239	mmu-miR-455-4395585	Target	19.99	2.15	10.05	2.81
240	mmu-miR-465a-3p-4395574	Target	30.00	0.49	20.06	1.88
241	mmu-miR-465a-5p-4373363	Target	NA	NA	NA	NA
242	mmu-miR-465b-5p-4395615	Target	28.21	0.22	18.27	1.83
243	rno-miR-466b-4395767	Target	NA	NA	NA	NA
244	rno-miR-466c-4395768	Target	29.00	0.23	19.06	1.83
245	mmu-miR-466h-4395646	Target	NA	NA	NA	NA
246	mmu-miR-467a-4395717	Target	20.98	3.90	11.03	4.30
247	mmu-miR-467c-4395647	Target	23.98	2.65	14.04	3.21
248	mmu-miR-467d-4395648	Target	24.00	2.38	14.06	2.99
249	mmu-miR-467e-4395698	Target	24.97	2.01	15.03	2.71
250	mmu-miR-470-4395718	Target	NA	NA	NA	NA
251	mmu-miR-484-4381032	Target	15.97	3.44	6.02	3.89
252	mmu-miR-486-4378096	Target	NA	NA	NA	NA
253	mmu-miR-487b-4378102	Target	34.97	1.28	25.03	2.22
254	mmu-miR-489-4378114	Target	22.99	3.05	13.04	3.55
255	mmu-miR-491-4381053	Target	24.98	1.44	15.04	2.31
256	mmu-miR-493-4395649	Target	27.97	2.71	18.03	3.26
257	mmu-miR-494-4395476	Target	20.97	2.45	11.03	3.05
258	mmu-miR-495-4381078	Target	19.96	3.08	10.02	3.57
259	mmu-miR-497-4381046	Target	21.98	2.90	12.04	3.42
260	mmu-miR-499-4381047	Target	25.23	0.21	15.29	1.83
261	mmu-miR-500-4395736	Target	22.99	1.99	13.05	2.69
262	mmu-miR-501-3p-4381069	Target	20.00	1.04	10.06	2.09
263	mmu-miR-503-4395586	Target	19.98	2.50	10.04	3.09
264	mmu-miR-504-4395195	Target	23.09	0.51	13.15	1.88
265	mmu-miR-505-4381071	Target	22.68	0.11	12.74	1.82
266	mmu-miR-509-3p-4395651	Target	29.92	0.22	19.98	1.83
267	mmu-miR-509-5p-4395650	Target	NA	NA	NA	NA
268	mmu-miR-511-4395679	Target	NA	NA	NA	NA
269	mmu-miR-532-3p-4395466	Target	17.99	3.16	8.05	3.64
270	mmu-miR-532-5p-4380928	Target	22.97	4.08	13.03	4.47
271	rno-miR-532-5p-4395752	Target	25.09	0.75	15.15	1.96
272	mmu-miR-539-4378103	Target	31.02	1.92	21.08	2.64
273	mmu-miR-540-3p-4378119	Target	30.97	0.13	21.03	1.82
274	mmu-miR-540-5p-4395691	Target	27.96	0.46	18.02	1.87
275	ath-miR159a-4373390	Target	NA	NA	NA	NA
276	mmu-miR-542-3p-4378101	Target	20.98	2.79	11.04	3.33
277	mmu-miR-542-5p-4395693	Target	26.99	1.65	17.05	2.46
278	mmu-miR-543-4395487	Target	20.95	2.76	11.00	3.30
279	rno-miR-543-4395766	Target	25.10	0.16	15.16	1.82
280	mmu-miR-544-4395680	Target	22.96	2.54	13.02	3.12
281	mmu-miR-551b-4380945	Target	28.98	2.65	19.04	3.21
282	mmu-miR-574-3p-4395460	Target	23.98	2.73	14.04	3.28
283	mmu-miR-582-3p-4395697	Target	28.00	2.10	18.05	2.77
284	mmu-miR-582-5p-4395696	Target	26.00	1.69	16.06	2.48
285	mmu-miR-590-5p-4395176	Target	27.45	0.11	17.51	1.82
286	mmu-miR-598-4395606	Target	26.96	1.50	17.02	2.36
287	rno-miR-598-5p-4395754	Target	29.00	0.88	19.05	2.02
288	mmu-miR-615-3p-4386777	Target	22.95	0.44	13.01	1.87
289	mmu-miR-615-5p-4395464	Target	28.92	0.47	18.98	1.87
290	mmu-miR-654-3p-4395350	Target	30.11	0.18	20.17	1.82
291	mmu-miR-654-5p-4395652	Target	NA	NA	NA	NA
292	mmu-miR-665-4395737	Target	27.00	1.88	17.06	2.61

293	mmu-miR-666-5p-4386770	Target	25.90	0.84	15.96	2.00
294	mmu-miR-669a-4381091	Target	23.97	2.71	14.03	3.26
295	mmu-miR-671-3p-4395433	Target	23.97	3.14	14.03	3.63
296	mmu-miR-672-4395438	Target	21.97	2.29	12.03	2.92
297	rno-miR-673-4395755	Target	29.96	1.23	20.02	2.19
298	mmu-miR-674-4395193	Target	22.99	1.62	13.05	2.43
299	mmu-miR-708-4395452	Target	18.97	3.04	9.03	3.54
300	mmu-miR-741-4395587	Target	29.04	0.56	19.10	1.90
301	mmu-miR-742-4395573	Target	NA	NA	NA	NA
302	rno-miR-742-4395756	Target	NA	NA	NA	NA
303	mmu-miR-743b-3p-4395601	Target	NA	NA	NA	NA
304	mmu-miR-743b-5p-4395600	Target	25.08	0.14	15.14	1.82
305	mmu-miR-743a-4395599	Target	21.23	0.16	11.29	1.82
306	rno-miR-743b-4395769	Target	NA	NA	NA	NA
307	mmu-miR-744-4395435	Target	18.95	3.64	9.01	4.07
308	rno-miR-758-4395180	Target	22.96	3.43	13.02	3.88
309	rno-miR-760-5p-4395758	Target	NA	NA	NA	NA
310	mmu-miR-770-3p-4395564	Target	25.08	0.23	15.13	1.83
311	mmu-miR-802-4395566	Target	NA	NA	NA	NA
312	mmu-miR-871-4395465	Target	NA	NA	NA	NA
313	rno-miR-871-4395770	Target	26.44	0.17	16.49	1.82
314	mmu-miR-872-4395375	Target	22.98	2.98	13.03	3.49
315	mmu-miR-873-4395467	Target	32.97	0.97	23.03	2.06
316	mmu-miR-874-4395379	Target	NA	NA	NA	NA
317	mmu-miR-875-3p-4395677	Target	NA	NA	NA	NA
318	mmu-miR-876-3p-4395594	Target	NA	NA	NA	NA
319	mmu-miR-876-5p-4395593	Target	NA	NA	NA	NA
320	rno-miR-878-4395771	Target	25.15	0.14	15.21	1.82
321	mmu-miR-878-5p-4395670	Target	NA	NA	NA	NA
322	mmu-miR-879-4395602	Target	NA	NA	NA	NA
323	mmu-miR-881-4395739	Target	NA	NA	NA	NA
324	rno-miR-881-4395773	Target	NA	NA	NA	NA
325	mmu-miR-883a-3p-4395591	Target	NA	NA	NA	NA
326	mmu-miR-883b-3p-4395695	Target	NA	NA	NA	NA
327	mmu-miR-883a-5p-4395741	Target	NA	NA	NA	NA
328	mmu-miR-125b-3p-4395489	Target	23.95	1.69	14.01	2.48
329	mmu-miR-143-4395360	Target	18.97	3.14	9.03	3.63
330	rno-miR-219-1-3p-4395778	Target	24.00	0.49	14.06	1.88
331	rno-miR-219-2-3p-4395501	Target	19.10	0.24	9.16	1.83
332	rno-miR-224-4373187	Target	16.95	2.09	7.01	2.77
333	mmu-miR-324-3p-4395639	Target	18.99	2.40	9.05	3.01
334	mmu-miR-351-4373345	Target	20.97	1.46	11.03	2.33
335	rno-miR-381-4381102	Target	25.02	2.33	15.08	2.95
336	rno-miR-409-5p-4381098	Target	NA	NA	NA	NA
337	rno-miR-450a-4381124	Target	25.96	1.59	16.02	2.41
338	mmu-miR-452-4373281	Target	21.83	0.15	11.89	1.82
339	mmu-miR-464-4373362	Target	NA	NA	NA	NA
340	mmu-miR-467b-4381084	Target	24.98	2.21	15.04	2.86
341	mmu-miR-468-4373366	Target	NA	NA	NA	NA
342	mmu-miR-469-4373367	Target	30.94	0.20	21.00	1.82
343	mmu-miR-488-4381074	Target	24.00	1.84	14.05	2.59
344	mmu-miR-490-4373215	Target	23.47	0.13	13.53	1.82
345	mmu-miR-496-4386771	Target	25.01	1.37	15.07	2.27
346	rno-miR-505-4381097	Target	27.90	0.12	17.95	1.82
347	mmu-miR-546-4381044	Target	32.98	0.24	23.04	1.83
348	mmu-miR-547-4395694	Target	28.95	0.12	19.00	1.82
349	mmu-miR-652-4395463	Target	17.99	3.26	8.05	3.73
350	mmu-miR-667-4386769	Target	21.95	2.34	12.01	2.96
351	mmu-miR-668-4386767	Target	25.99	1.14	16.05	2.14

352	mmu-miR-670-4395561	Target	27.94	0.20	18.00	1.82
353	mmu-miR-675-3p-4386762	Target	20.98	1.70	11.04	2.48
354	mmu-miR-675-5p-4386761	Target	NA	NA	NA	NA
355	mmu-miR-676-4386776	Target	19.96	2.46	10.02	3.06
356	mmu-miR-677-4381075	Target	28.98	1.95	19.04	2.66
357	mmu-miR-679-4381077	Target	25.97	1.15	16.03	2.15
358	mmu-miR-680-4381079	Target	25.34	0.11	15.40	1.82
359	mmu-miR-682-4381081	Target	24.96	0.59	15.02	1.91
360	mmu-miR-683-4381082	Target	NA	NA	NA	NA
361	mmu-miR-684-4381083	Target	27.99	2.23	18.04	2.88
362	mmu-miR-685-4386748	Target	20.00	1.43	10.06	2.31
363	mmu-miR-686-4381085	Target	NA	NA	NA	NA
364	mmu-miR-687-4386750	Target	NA	NA	NA	NA
365	mmu-let-7a-4373169	Target	23.00	0.85	13.06	2.00
366	mmu-let-7f-4373164	Target	21.02	0.49	11.08	1.88
367	mmu-miR-106b-4373155	Target	13.98	3.29	4.04	3.76
368	mmu-miR-155-4395701	Target	23.97	3.82	14.02	4.23
369	mmu-miR-17-4395419	Target	12.99	3.24	3.04	3.71
370	mmu-miR-23a-4373074	Target	NA	NA	NA	NA
371	mmu-miR-29a-4395223	Target	23.96	3.08	14.01	3.57
372	mmu-miR-30a-4373061	Target	16.95	3.28	7.01	3.75
373	mmu-miR-30e-4395334	Target	19.95	3.66	10.01	4.09
374	mmu-miR-31-4373331	Target	18.98	2.96	9.04	3.47
375	mmu-miR-34b-3p-4395748	Target	19.96	3.22	10.02	3.69
376	mmu-miR-92a-4373013	Target	11.97	4.17	2.03	4.55
377	rno-miR-190b-4395749	Target	25.95	2.79	16.01	3.33
378	mmu-miR-129-5p-4373171	Target	26.11	0.13	16.17	1.82
379	mmu-miR-187-4373307	Target	27.02	0.37	17.07	1.85
380	mmu-miR-188-3p-4395217	Target	NA	NA	NA	NA
381	mmu-miR-106a-4395589	Target	12.99	2.76	3.05	3.31
382	mmu-miR-188-5p-4395431	Target	23.97	2.34	14.03	2.96
383	mmu-miR-302b-4378071	Target	18.98	2.68	9.03	3.24
384	mmu-miR-322-4378107	Target	19.95	2.51	10.01	3.10

Supplementary Table 1.4.2 Heat Map for Lung miRNA-Signature

Relative abundance:

High Low

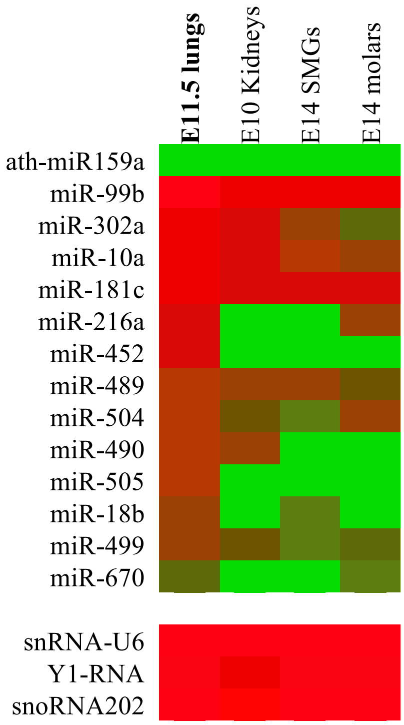

Supplementary Table 2 – Gene Ontology (GO) and scores (q-value) for the predicted miR-590-5p targets. Palate Development and Odontogenesis GO entries (with the lowest q-values) are shown on the top of the list.

**GO id**	**Description**	**q-value**
GO:0060021	palate development	6.0E-10
GO:0042476	odontogenesis	5.7E-05
GO:0035265	organ growth	1.4E-04
GO:0050678	regulation of epithelial cell proliferation	1.7E-04
GO:0033674	positive regulation of kinase activity	1.9E-04
GO:0005911	cell-cell junction	2.1E-04
GO:0051216	cartilage development	2.3E-04
GO:0043566	structure-specific DNA binding	5.3E-04
GO:0050673	epithelial cell proliferation	7.2E-04
GO:2000027	regulation of organ morphogenesis	8.5E-04
GO:0060324	face development	1.0E-03
GO:0048706	embryonic skeletal system development	1.2E-03
GO:0021537	telencephalon development	1.2E-03
GO:0034622	cellular macromolecular complex assembly	1.4E-03
GO:0016337	cell-cell adhesion	1.4E-03
GO:0005769	early endosome	1.5E-03
GO:0030855	epithelial cell differentiation	1.5E-03
GO:0003924	GTPase activity	1.6E-03
GO:0007568	aging	1.6E-03
GO:0060415	muscle tissue morphogenesis	1.6E-03
GO:0051155	positive regulation of striated muscle cell differentiation	1.7E-03
GO:0051090	regulation of sequence-specific DNA binding transcription factor activity	1.9E-03
GO:0043524	negative regulation of neuron apoptosis	2.0E-03
GO:0035591	signaling adaptor activity	2.1E-03
GO:0010171	body morphogenesis	2.1E-03
GO:0044456	synapse part	2.1E-03
GO:0030837	negative regulation of actin filament polymerization	2.2E-03
GO:0045860	positive regulation of protein kinase activity	2.3E-03
GO:0060325	face morphogenesis	2.5E-03
GO:0048732	gland development	2.6E-03
GO:0034504	protein localization to nucleus	2.8E-03
GO:0035239	tube morphogenesis	2.8E-03
GO:0071696	ectodermal placode development	2.8E-03
GO:0071697	ectodermal placode morphogenesis	2.8E-03
GO:0032956	regulation of actin cytoskeleton organization	2.8E-03
GO:0043523	regulation of neuron apoptosis	2.8E-03
GO:0060788	ectodermal placode formation	2.8E-03
GO:0021543	pallium development	2.9E-03
GO:0001837	epithelial to mesenchymal transition	2.9E-03
GO:0009952	anterior/posterior pattern specification	2.9E-03
GO:0042063	gliogenesis	3.0E-03
GO:0010741	negative regulation of intracellular protein kinase cascade	3.1E-03
GO:0044087	regulation of cellular component biogenesis	3.3E-03
GO:0071901	negative regulation of protein serine/threonine kinase activity	3.3E-03
GO:0032970	regulation of actin filament-based process	3.3E-03
GO:0019003	GDP binding	3.7E-03
GO:0000987	core promoter proximal region sequence-specific DNA binding	3.7E-03
GO:0051493	regulation of cytoskeleton organization	3.7E-03
GO:0048566	embryonic digestive tract development	3.7E-03
GO:0043409	negative regulation of MAPK cascade	3.7E-03
GO:0005525	GTP binding	3.7E-03
GO:0060536	cartilage morphogenesis	3.8E-03

Supplementary Table 3 | Selected genes for expression screening after antagomir treatments

Gene	Gene Ontology	Targeting miRNA	Reference
Apc	Odontogenesis<br>Epithelial morphogenesis	-	Wang et al, Development 2009
Bcl11b	Odontogenesis<br>Enamel morphogenesis	miR-590-5p	Kyrylkova et al, PLoS One 2012
Bmp4	Odontogenesis	-	Bei et al, Development 2000
Bmpr1a	Odontogenesis	-	He et al, Dev. Biol. 2010
Casp3	Apoptosis,<br>Upregulated in miR-21 knockout mice	-	Zhou et al, Oncol. Rep. 2010
Chd7	Co-regulated with Sox2	miR-590-5p	Puc et al, Nat. Genet. 2011
Gli1	Shh signaling<br>Enamel regulation	-	Gritli-Linde et al, Development 2002
Gli2	Shh signaling<br>Enamel regulation	-	Hardcastle et al, Development 1998
Gli3	Shh signaling<br>Enamel regulation	-	Hardcastle et al, Development 1998
Msx1	Odontogenesis	miR-590-5p	Chen et al, Development 1996
Nog	Direct miR-200c target gene	miR-200c	Cao et al, Development 2013
Pax9	Odontogenesis	-	Nakatomi et al, Dev. Biol. 2010
Pdcd4	Direct miR-21 target<br>Cell death regulation	miR-21	Ahmed et al, J. Cell Sci. 2011
Pitx2	Odontogenesis	miR-590-5p	Cao et al, Development 2013
Sox2	Progenitor stem cell marker	-	Wang et al, Development 2009

Supplementary Table 4a – Indirect* methods of miRNA target validation

mRNA methods	Advantage	Disadvantage
qPCR	Fast preliminary screening	Low throughput<br>Limited to predicted targets
Northern blot	Fast preliminary screening	Low throughput<br>Limited to predicted targets
In Situ Hybridization	Fast preliminary screening<br>Tissue distribution information	Low throughput<br>Limited to predicted targets
Microarrays	High throughput<br>Discovery new unpredicted targets	Data analysis is time-consuming
RNA-Seq	High throughput<br>Discovery new unpredicted targets	Data analysis is time-consuming

Protein methods	Advantage	Disadvantage
Western blot	Fast preliminary screening	Low throughput
Mass Spectroscopy	High throughput<br>Discovery new unpredicted targets	Data analysis is time-consuming

* Indirect inferences from changes in gene expression

Supplementary Table 4b – Direct** methods of miRNA target validation

Method	Advantage	Disadvantage
Luciferase Reporter	miRNA seeding region binding screening	No biological context<br>Set up is time consuming<br>Low throughput<br>Limited to predicted targets
miRNA Pulldown (miR-PD)	Biological context of interest<br>Set up is fast<br>Data analysis is fast<br>Discovery new unpredicted targets<br>High throughput***	Small amount of recovered RNA
Ribosome Profiling	Biological context of interest<br>Discovery new unpredicted targets<br>High throughput***	Set up is time-consuming<br>Data analysis is time-consuming
Crosslinking Immunoprecipitation (CLIP)	Biological context of interest<br>Discovery new unpredicted targets<br>High throughput***	Set up is time-consuming<br>Data analysis is time-consuming

** Takes in consideration miRNA:mRNA interactions and the corresponding cellular context
*** When coupled with Microarrays or RNA-Seq

Supplementary Table 5 – Antagomirs and Biotinylated Mimics

Antagomirs from Exiqon

miRNA target	Catalog number	Stock solution
Negative Control (Off-target)	199004-00	5 µM
miR-590-5p	412180-00	5 µM
miR-325-3p	410918-00	5 µM
miR-183-3p	410096-00	5 µM
miR-429-3p	410693-00	5 µM
miR-200c-3p	410126-00	5 µM

Biotinylated Mimics from Dharmacon

miRNA target	Catalog number	Stock solution
Negative Control (Off-target)	Custom miRIDIAN mimic	5 µM
miR-590-5p	Custom miRIDIAN mimic	5 µM
miR-200c-3p	Custom miRIDIAN mimic	5 µM

Note: all biotinylated mimics are pre-ordered upon request from Dharmacon (Thermo Scientific)

Supplementary Table 6 | Preparation of whole mount and mandible cryosections for immunofluorescence

**Whole mount (SMGs, Lungs, Kidneys)**	**Mandible cryosections (Molar Germs, Incisors)**
i. Carefully remove the PFA-fixed organs from the Nuclepore filters using forceps<br>ii. Place the organs in a well of a multiwell plate (12 wells) containing 1 mL of Tween 0.3%<br>iii. Incubate for 20 min at room temperature<br>iv. Pipette out the Tween 0.3% solution<br>v. Pipette 1 mL of IF Blocking Buffer*<br>vi. Incubate for 1 h at room temperature<br>vii. Pipette out the IF Blocking Buffer<br>viii. Pipette 1 mL of primary antibody (anti-mouse E-Cadherin or anti-rat EpCam, at 1:400 or 1:1,000 dilutions, respectively). Pipette 80 µl/mL of MOM Protein mix supplied by the MOM reagent per antibody incubation<br>ix. Incubate in a rocker at 4 °C, overnight<br>x. Pipette out the primary antibodies. Add 1 mL of BSA<br>xi. Optional: perform EdU detection following the Click-it EdU detection kit specifications<br>xii. Proceed to secondary antibody incubation by pipetting the corresponding donkey anti-mouse or donkey anti-rat fluorescently labeled antibody** (dilution 1:200 in PBS), and incubate for 1 h at room temperature (protected from the light)<br>xiii. Wash the secondary antibody solution with PBS, 3 times, and mount the organs in glass slides for microscope	i. Carefully remove the PFA-fixed mandibles from the Trowell-type organ explant plates using forceps<br>ii. Place the mandibles in a well a multiwell plate (12 wells) containing 1 mL of Sucrose (30% solution m/v in PBS)<br>iii. Incubate overnight at 4 °C in a rocker<br>iv. Use cryomolds containing volumes of O.C.T enough to cover the mandibles.<br>v. Transfer the mandibles from the multiwell plates to the molds using forceps<br>vi. Position the mandibles for coronal or sagittal sectioning<br>vii. Place the cryomolds in dry ice (use immediately or store the molds at -80 °C)<br>viii. Proceed to cryosections following the Cryostat Leica CM 3050S specifications<br>ix. Collect 14 µ thick sections in glass slides for microscopy<br>x. Seal the border of the glass slides with a hydrophobic pen<br>xi. Wash the slides in PBS solution, 3 times, to remove the OCT<br>xii. Proceed to the steps v-xiii as in Whole Mount, using 500 µL of all solutions per slide<br>xiii. Mount the slides for microscopy analysis using mounting medium

* Blocking Buffer: 10% Heat-Inactivated Donkey Serum, 10% BSA, Mouse On Mouse Blocking Reagent (18µL/mL, Vector, cat.no. MKB-2213), all diluted in PBS (0.1% Tween).

**Secondary Antibodies: all from Jackson Immuno Research, and used as previously tested.[5] [6]

Supplementary Table 7 – qRT-PCR Primers

**qRT-PCR Primers for mRNA expression analysis**		
**Gene**	**Forward Sequence**	**Reverse Sequence**
Rps29	GGAGTCACCCACGGAAGTTCGG	GGAAGCACTGGCGGCACATG
Chd7	GCCTTTCACGCACACCCACAA	CCTCCTGCCTCGCCTCCTC
Msx1	CCTCACTCTACAGTGCCTCT	CCGCTGCTCTGCTCAAAG
Bcl11b	ATGGGCCGAGGATACCCACT	AGGCTGGGGGCTGAAAAGAG
Pitx2	CTGAGAATTGTGCTAGAAGGTCGT	CAGTCAAACATTTGTGGGCGTAC
Shh	GAGCAGACCGGCTGATGACTCAGAG	GTCCCGGTCGGACGTGGT
Sox2	CAGCATGTCCTACTCGCAGCAG	TGGAGTGGGAGGAAGAGGTAACC
Bmp4	GAGATCAGGCAGTCCAGAGG	TGGTATGTGTAGGTGGTTGAATG

Notes:
1. All primers are stored as 20 µM stock solutions at -20 °C, and diluted prior use
2. All primers are ordered from Invitrogen

**qRT-PCR Primers for miRNA expression analysis**	
**miRNA**	**Catalog number**
miR-590-5p	MS00012404
miR-200c-3p	MS00001827
miR-183-3p	MS00011298
miR-325-3p	MS00011788
miR-429-3p	MS00002366
snRNA-U6 (Control 1)	MS00033740
snord61 (Control 2)	MS00033705

Note: all primers for miRNA are from Qiagen (sequence provided upon request)

qPCR cycling conditions for miRNA and mRNA detection

**miRNA detection**			**mRNA detection**		
**Step**	**Time**	**Temperature**	**Step**	**Time**	**Temperature**
1. Initiation	15 min	95 °C	1. Initiation	3 min	95 °C
2. Denaturation	30 s	95 °C	2. Denaturation	30 s	95 °C
3. Annealing	30 s	55 °C	3. Annealing	30 s	65 °C
4. Extension	30 s	70 °C	4. Extension	30 s	72 °C
5. Repeat steps 2-4	40 X		5. Repeat steps 2-4	35 X	
6. Inactivation	5 min	95 °C	6. Inactivation		95 °C

Supplementary Table 8 – Statistical Analysis

Fig. 3b Statistical Analysis: Student's T-Test (one-tailed, unpaired)
Experiment: qRT-PCR analysis of miR-200c and miR-590-5p expression after transfections of antagomirs in molar germs (mandible cultures) using nanoparticles (**NFS**) or liposomes (**LFS**).
Comparisons: miR-200c expression using NFS versus LFS, and miR-590-5p expression using NFS versus LFS.
Organ model: **molar tooth germs** (E13.5 mandible culture explants)

qRT-PCR Fold Change (NFS transfections)

miRNA	Triplicate	Control Group Anta-Off	Experimental group Anta-200c	Experimental group Anta-590
U6-snRNA	1	1.0	1.0	1.0
	2	1.0	1.0	1.0
	3	1.0	1.0	1.0
snoRNA-D61	1	1.0	0.9	1.2
	2	1.0	0.9	1.4
	3	1.0	1.0	1.3
miR-200c-3p	1	1.0	0.2	1.0
	2	1.0	0.1	1.0
	3	1.0	0.2	1.0
miR-590-5p	1	1.0	1.2	0.3
	2	1.0	0.9	0.2
	3	1.0	1.0	0.2

PCR Fold Change (LFS transfections)

miRNA	Triplicate	Control Group Anta-Off	Experimental group Anta-200c	Experimental group Anta-590
U6-snRNA	1	1.0	1.0	1.0
	2	1.0	1.0	1.0
	3	1.0	1.0	1.0
snoRNA-D61	1	1.0	1.1	1.1
	2	1.0	1.3	1.2
	3	1.0	1.1	1.1
miR-200c-3p	1	1.0	0.5	0.8
	2	1.0	0.6	1.0
	3	1.0	0.6	0.9
miR-590-5p	1	1.0	0.9	0.6
	2	1.0	1.0	0.6
	3	1.0	0.9	0.6

Significance by default

ns $p>0.05$
* $p<0.05$
** $p<0.01$
*** $p<0.001$

T-Student Test (one-tailed, unpaired) for miRNA expression after A-200c and A-590 transfections (Comparison: NFS versus LFS transfection methods)

miRNA, gene	p-value (NFS versus LFS) A-200c	Significance	p-value (NFS versus LFS) A-590	Significance
U6-snRNA	ns	ns	ns	ns
snoRNA-D61	0.100	ns	0.141	ns
miR-200c-3p	0.006	**	0.214	ns
miR-590-5p	0.220	ns	0.010	**

Supplementary Table 8 – Statistical Analysis

Fig.3b Statistical Analysis: Student's T-Test (one-tailed, unpaired)
Experiment: qRT-PCR analysis of miR-200c and miR-590-5p expression after transfections of antagomirs in molar germs (mandible cultures) using nanoparticles (**NFS**) or liposomes (**LFS**).
Comparisons: miR-200c expression using NFS versus LFS, and miR-590-5p expression using NFS versus LFS.
qRT-PCR calculations: delta-delta CT

Organ model: **incisors** (E13.5 mandible culture explants)

qRT-PCR Fold Change values

miRNA	Triplicate	Control Group Anta-Off	Experimental group Anta-200c	Experimental group Anta-590
U6-snRNA	1	1.0	1.0	1.0
	2	1.0	1.0	1.0
	3	1.0	1.0	1.0
snoRNA-D61	1	1.0	0.8	1.0
	2	1.0	1.2	1.2
	3	1.0	0.9	0.9
miR-200c-3p	1	1.0	0.3	0.8
	2	1.0	0.4	1.0
	3	1.0	0.2	0.8
miR-590-5p	1	1.0	0.9	0.4
	2	1.0	0.9	0.3
	3	1.0	1.0	0.3

PCR Fold Change values

miRNA	Triplicate	Control Group Anta-Off	Experimental group Anta-200c	Experimental group Anta-590
U6-snRNA	1	1.0	1.0	1.0
	2	1.0	1.0	1.0
	3	1.0	1.0	1.0
snoRNA-D61	1	1.0	1.0	0.9
	2	1.0	0.9	0.9
	3	1.0	1.0	0.9
miR-200c-3p	1	1.0	0.7	1.3
	2	1.0	0.6	1.2
	3	1.0	0.6	1.1
miR-590-5p	1	1.0	1.2	0.6
	2	1.0	1.1	0.7
	3	1.0	1.0	0.7

Significance by default

ns $p>0.05$
* $p<0.05$
** $p< 0.01$
*** $p<0.001$

T-Student Test (one-tailed, unpaired) for miRNA expression after A-200c and A-590 transfections
(Comparison: NFS versus LFS transfection methods)

miRNA, gene	p-value (NFS versus LFS) A-200c	Significance	p-value (NFS versus LFS) A-590	Significance
U6-snRNA	ns	ns	ns	ns
snoRNA-D61	0.471	ns	0.100	ns
miR-200c-3p	0.010	**	0.072	ns
miR-590-5p	0.100	ns	0.006	**

Supplementary Table 8 – Statistical Analysis

Fig.3b Statistical Analysis: Student's T-Test (one-tailed, unpaired)
Experiment: qRT-PCR analysis of miR-200c and miR-590-5p expression after transfections of antagomirs in molar germs (mandible cultures) using nanoparticles (**NFS**) or liposomes (**LFS**).
Comparisons: miR-200c expression using NFS versus LFS, and miR-590-5p expression using NFS versus LFS.
Organ model: **salivary glands** (E13.5 tongue + salivary glands explants)

qRT-PCR Fold Change (NFS transfections)

miRNA	Triplicate	Control Group Anta-Off	Experimental group Anta-200c	Experimental group Anta-590
U6-snRNA	1	1.0	1.0	1.0
	2	1.0	1.0	1.0
	3	1.0	1.0	1.0
snoRNA-D61	1	1.0	1.1	1.4
	2	1.0	1.3	1.2
	3	1.0	1.1	1.2
miR-200c-3p	1	1.0	0.2	1.4
	2	1.0	0.3	1.1
	3	1.0	0.2	1.1
miR-590-5p	1	1.0	1.0	0.2
	2	1.0	1.2	0.3
	3	1.0	1.0	0.2

PCR Fold Change (LFS transfections)

miRNA	Triplicate	Control Group Anta-Off	Experimental group Anta-200c	Experimental group Anta-590
U6-snRNA	1	1.0	1.0	1.0
	2	1.0	1.0	1.0
	3	1.0	1.0	1.0
snoRNA-D61	1	1.0	1.3	1.0
	2	1.0	1.2	0.9
	3	1.0	1.3	0.6
miR-200c-3p	1	1.0	0.6	0.9
	2	1.0	0.6	0.8
	3	1.0	0.7	0.8
miR-590-5p	1	1.0	1.0	0.5
	2	1.0	1.0	0.6
	3	1.0	0.9	0.7

Significance by default

ns p>0.05
* p<0.05
** p< 0.01
*** p<0.001

T-Student Test (one-tailed, unpaired) for miRNA expression after A-200c and A-590 transfections (Comparison: NFS versus LFS transfection methods)

miRNA, gene	p-value (NFS versus LFS) A-200c	Significance	p-value (NFS versus LFS) A-590	Significance
U6-snRNA	ns	ns	ns	ns
snoRNA-D61	0.230	ns	0.041	*
miR-200c-3p	0.011	**	0.080	ns
miR-590-5p	0.270	ns	0.004	***

Supplementary Table 8 – Statistical Analysis

Fig.4g-h Statistical Analysis: Student's T-Test (one-tailed, unpaired).
Experiment: qRT-PCR analysis of the expression of miR-590-5p targets and co-expressed genes after transfections of antagomirs (**g**) and mimics (**h**) in molar germs (mandible cultures) using nanoparticles.
Comparisons: gene expression (Fold Change values) normalized to a housekeeping gene (*Rps29*) and to an off target antagomir (**g**) or mimic (**h**) controls. qRT-PCR calculations: delta-delta CT

qRT-PCR Fold Change Values

Gene	Triplicate	Mimic Control	Mimic-590	Antagomir Control	Antagomir-590
Rps29	1	1.0	1.0	1.0	1.0
	2	1.0	1.0	1.0	1.0
	3	1.0	1.0	1.0	1.0
Chd7	1	1.0	0.3	1.0	2.3
	2	1.0	0.4	1.0	2.0
	3	1.0	0.4	1.0	1.9
Msx1	1	1.0	0.4	1.0	2.1
	2	1.0	0.4	1.0	1.4
	3	1.0	0.6	1.0	2.1
Bcl11b	1	1.0	0.5	1.0	1.7
	2	1.0	0.5	1.0	1.1
	3	1.0	0.6	1.0	1.4
Pitx2	1	1.0	0.7	1.0	0.6
	2	1.0	0.8	1.0	0.7
	3	1.0	1.1	1.0	0.9
Shh	1	1.0	0.6	1.0	1.3
	2	1.0	0.7	1.0	0.8
	3	1.0	0.8	1.0	1.6
Sox2	1	1.0	1.1	1.0	2.0
	2	1.0	0.9	1.0	1.4
	3	1.0	0.8	1.0	2.1
Bmp4	1	1.0	0.3	1.0	1.9
	2	1.0	0.4	1.0	1.1
	3	1.0	0.4	1.0	1.6

Significance by default

ns p>0.05
* p<0.05
** p< 0.01
*** p<0.001

T-Student Test (one-tailed, unpaired) comparing gene expression using Mimic-590 versus Mimic-Off-target, and Antagomir-590 versus Antagomir-Off-target

Gene	p-value (Mimic-590 versus Mimic-Off-target)	significance	p-value (Antagomir-590 versus Antagomir-Off-target)	significance
Chd7	0.00002	***	0.00058	***
Msx1	0.00038	***	0.01038	**
Bcl11b	0.00024	***	0.03208	*
Pitx2	0.21540	ns	0.02826	ns
Shh	0.06550	ns	0.17361	ns
Sox2	0.27859	ns	0.00937	**
Bmp4	0.00000	***	0.03025	*

Supplementary Table 8 – Statistical Analysis

Fig.4i Statistical Analysis: Student's T-Test (one-tailed, unpaired).
Experiment: qRT-PCR analysis of miR-590-5p expression after transfections of antagomirs and mimics in molar germs (mandible cultures) using nanoparticles.
Comparisons: miR-590-5p expression (Fold change values) between Off-target (A-Off) versus miR-590-5p (A-590) antagomirs, and Off-target (M-Off) and miR-590-5p (M-590) mimics. qRT-PCR calculations: delta-delta CT

T-Student Test (one-tailed, unpaired) comparing miR-590-5p expression using Antagomir-590 versus Antagomir-Off-target, and Mimic-590 versus Mimic-Off-target

Gene	Triplicate	Fold change (A-Off)	Fold change (A-590)	p-value (A-Off versus A-590)	Significance	Fold change (M-Off)	Fold Change (M-590)	p-value (M-Off versus M-590)	Significance
miR-590-5p	1	1.00	0.23	0.001241936	***	1.00	955.43	0.001229677	***
	2	1.00	0.29			1.00	797.86		
	3	1.00	0.15			1.00	564.18		

Significance by default

ns p>0.05
* p<0.05
** p< 0.01
*** p<0.001

Supplementary Table 8 – Statistical Analysis

Fig.4j Statistical Analysis: Student's T-Test (one-tailed, unpaired).
Experiment: qRT-PCR analysis of enrichment of genes detected after micro-RNA pulldown (miR-PD) analysis using miR-590-5p biotinylated mimic in molar tooth germs (mandible explants).
Comparisons: enrichment (in percentage) of miR-590-5p targets and co-regulated genes using the pulldown fractions (PD) of miR-590-5p-biotin and an off-target-miRNA-biotin.
qRT-PCR calculations: delta-delta CT

Percentage of detection<br>Gene	Triplicate	Off-Target PD	miR-590-5p PD
Rps29	1	100.0	100.0
	2	100.0	100.0
	3	100.0	100.0
Chd7	1	100.0	246.2
	2	100.0	263.9
	3	100.0	141.4
Msx1	1	100.0	348.2
	2	100.0	459.5
	3	100.0	373.2
Bcl11b	1	100.0	246.2
	2	100.0	303.1
	3	100.0	174.1
Pitx2	1	100.0	81.2
	2	100.0	53.6
	3	100.0	75.8
Shh	1	100.0	ND
	2	100.0	ND
	3	100.0	ND
Sox2	1	100.0	57.4
	2	100.0	162.5
	3	100.0	107.2
Bmp4	1	100.0	162.5
	2	100.0	123.1
	3	100.0	81.2

T-Student Test (one-tailed, unpaired) comparing gene expression of pulldown fractions using miR-590-5p-biotin versus Off-Target-miRNA-biotin control

Gene	p value<br>(miR-590-5p PD versus miR-off target PD)	Statistical significance	
Chd7	0.046	*	
Msx1	0.006	**	**Significance by default**
Bcl11b	0.032	*	ns p>0.05
Pitx2	0.360	ns	* p<0.05
Shh	#DIV/0!	ns	** p< 0.01
Sox2	0.397	ns	*** p<0.001
Bmp4	0.221	ns	

Supplementary Figure Legends

Supplementary Figure 1 | Protocol for transfections of miRNA antagomirs or mimics using peptide-based nanoparticles. (**a**) Typically, antagomirs and mimics are used at an initial concentration of 5 μM, and diluted with Transfection Buffer (Solution 1) to 1.3 μM. The amphiphilic N-TER peptide is diluted in water (Solution 2), and the solutions 1 and 2 are mixed to form Nanoparticle-Forming Solution (NFS) at a final concentration of 650 nM of antagomir or mimic. (**b**) Table shows that ratio of N-TER and antagomir or mimic can be modified from 5:1 (highlighted, recommended in this method) to 2:1 (more cargo molecules, less N-TER peptide), and 20:1 (less cargo molecules, more N-TER peptide). For example, transfections using cell lines require much less cargo molecule, and a ratio of 20:1 N-TER and cargo molecule can be used. Note: the N-TER peptide itself does not self-assemble in the absence of cargo molecules. (**c**) Volumes of NFS prepared using a 5:1 ratio of N-TER peptide and cargo molecules used for transfections to obtain 10, 50, or 100 nM final concentrations of antagomirs or mimics in organ explant cultures. The final volume of culture medium is 2.0 mL. See METHODS for details.

Supplementary Figure 2 | Flowchart of miRNA target gene validation using biotinylated mimics and miRNA pulldown assay (miR-PD). (**a**) The miR-PD method consists of directly detecting the mRNAs that interact with a biotinylated miRNA mimic using a pulldown protocol. Lysis buffer is optimized to recover the cytoplasmic fraction (Input) separated from the nuclear fraction (Pellet). The final output of this protocol is highlighted (in orange) and derives from the cytoplasmic lysate (Input Fraction). From the Input Fraction, miRNA targets (mRNAs) and RNAi machinery proteins (pulldown or PD Fraction) are separated from the rest of the cytoplasmic fraction (Input – PD Fraction), and both PD and Input – PD Fractions are used to

calculate the relative enrichment of mRNAs by qRT-PCR analysis. (**b**) Flowchart shows steps of qRT-PCR calculations to obtain enrichment ratios that compare mRNAs found in the PD and in the Input-PD Fractions.

Supplementary Fig. 3 | Embryonic organ collection and generation of miR expression signature for molar tooth germs using TaqMan Low Density Arrays (TLDA). (**a**) Representative picture of mouse embryos at the corresponding times of organ collection. The table below the picture shows RNA extraction efficiencies using the miRvana total RNA extraction kit^{TM} (Ambion). (**b**) Selected heat map of highly expressed miRNAs in the molar tooth germs compared to other organs (detailed heat map and TLDA data are available in Suppl. Table 1). Housekeeping RNAs U6-RNA and snoRNA202 have similar relative abundances in all organs and can be used as housekeeping genes. Some miRNAs such as miR-429-3p, miR-325-3p, miR-590-5p and miR-200c-3p show higher relative expression in molar tooth germs when compared to other organs, while miR-687, miR-129-5p, miR-297b-5p, miR-546, and miR-743b-3p show specific expression in molar germs with very low or absent expression in other tissues. (**c**) E13.5 epithelium and mesenchyme tissues are separated using Dispase treatment, and relative miRNA expression (shown as percent of expression in epithelium) is detected by qRT-PCR. The graph shows that the miRNAs selected for this study have differential expression in epithelium (miR-429-3p, miR-590-5p, and miR-200c-3p) compared to miR-325-3p (more abundant in mesenchyme).

Supplementary Fig. 4 | Optimization of antagomir transfections using Liposome-Forming Solution (LFS) and peptide-based Nanoparticle-Forming Solution (NFS). E11.5 lungs (**a**) and E13.5 SMGs (**b**) were explanted in floating filters and transfected with a fluorescently labeled off-target antagomir miRNA (Antagomir-Cy3) using LFS and NFS. Embryonic organs were fixed after 48 h and used for immunofluorescence analysis. Epithelial Cell Adhesion molecule (EpCam) was used as an epithelial marker. A set of representative images of embryonic lungs and SMGs were used in each experimental group. Cy3 and Alexa-488 fluorescent images were quantified using ImageJ. NFS shows more efficiency in transfecting Antagomir-Cy3 than LFS, as inferred by the quantification graphs. In the graphs, red bars correspond to Cy3 detection (red fluorescence), green bars correspond to EpCam detection (green fluorescence), and orange bars are normalized ratios of Antagomir-Cy3/EpCam detection (red fluorescence normalized to green fluorescence). Scale bar: 100 μ. (**c-d**) Transfection of fluorescently labeled antagomir in embryonic lung (c) and kidney (d) explants using nanoparticles. Embryonic organs were harvested and explanted in floating filters on top of DMEM-F12 supplemented with antibiotics as previously described. The representative figures show a fluorescently labeled antagomir (Antagomir-Cy3NA) transfected in embryonic lung and kidney explants. Transfections were independently performed using organ explants from different onset of development (E11.5, E12.5), and time of organ culture (24 h, 48 h). Final concentration of Antagomir-Cy3 is 100 nM.

Supplementary Fig. 5. Analysis of nanoparticle-transfected Antagomir-Cy3NA uptake and cell viability in the epithelium and in the mesenchyme of E13.5 embryonic mandibular explants using flow cytometry. (**a**) Overview of the experimental design. Embryonic E13.5

mandibles from a mouse strain that constitutively expresses Egfp (as in MATERIALS) were explanted, and off-target antagomirs, labeled or not with Cy3 (Antagomir-Cy3, and Off-Target Antagomir), were transfected using N-TER nanoparticles. A third control, corresponding to non-transfected explants (untreated) was used. The explants were cultured (24 h) and epithelial-mesenchyme tissue dissections performed using conventional Dispase neutral proteases protocol, followed by single-cell preparations of the epithelium and the mesenchyme tissues. (**b**) The representative picture shows an E13.5 EGFP-expressing mandible, after Antagomir-Cy3 (100 nM) transfection using nanoparticles, and subjected to Dispase epithelial and mesenchymal tissue separation. The arrow in the higher magnification detailed picture shows the separated epithelium region of the molar tooth germs. OE: Oral Epithelium, M: Mesenchyme, MM: Molar Mesenchyme, ME: Molar Epithelium. (**c**) Gene expression (qRT-PCR) of epithelial and mesenchymal markers in single-cell preparations from the epithelium (red) and the mesenchyme (blue) tissues of E13.5 mandible explants (24 h), after transfection of Antagomir-Cy3 antagomir (group 1, graph on the left), an unlabeled antagomir control (group 2, graph in the middle), or untreated mandibles (group 3, left graph). Gene expression of epithelial markers (*Cdh1*, *Fgfr2b*, *Lama5*), and mesenchymal markers (*Fgfr2c*, *Vm1*, *Msx1*), did not show significant differences among the experimental groups, and suggested that nanoparticle-based transfections had no effect in gene expression. (**d-e**) Flow cytometry analysis of epithelial (e) and mesenchymal (e) single cell suspensions after transfections of antagomirs (Antagomir-Cy3 or unlabeled control, groups 1 and 2, respectively), and untreated mandible explants (group 3). The results were representative of 4 biological replicates combined (n=4). Cell viability (Propidium Iodide staining, PPI) of the epithelial single cell suspensions in the groups 1, 2 and 3, were, respectively, 87.4%, 87.2%, and 75.5% (Suppl. Fig. 5d), and for the mesenchymal single cell

suspensions in the groups 1, 2, and 3, were 96.4%, 95.8%, and 94.6% (Suppl. Fig. 5e), which suggests that nanoparticle-based transfections had negligible off-target effects (such as cell death and stress-induced apoptosis). The Antagomir-Cy3 uptake in epithelial cells (81.2%, Suppl. Fig. 5d), and in mesenchymal cells (93.2%, Suppl. Fig. 5e) suggested a highly efficient transfection throughout the tissues in the organ explants.

Supplementary Fig. 6 | *In silico* prediction of miRNA targeting using the homology-based database search TargetScan and the Gene Ontology-based miRNA target ranking approach. (**a**) Examples of molar tooth germ miR-signature genes miR-325-3p, miR-590-5p, and miR-429-3p used for miRNA target prediction. Orange arrows indicate number of miRNA targets after prediction and filtering (in green). (**b**) Flow diagram showing the CytoScape and GeneMania plugin used for Gene Ontology analysis. (**c**) q-Value scores showing Gene Ontology Identification (GO ID) numbers, and the corresponding Descriptions (Biological Processes). (**d**) Gene Interactions diagram. Predicted (orange lines) and physical interactions (red lines), and co-expression (purple lines, not tissue expression), and miRNA targets and non-targets are also shown.

Supplementary Fig. 7 | Specificity of antagomir-induced downregulation of target miRNAs. miRNA expression analysis by qRT-PCR after miR-590-5p and miR-21 antagomir transfections in molar tooth germs from E13.5 mandible explants, showed a selective decrease of miR-590-5p and miR-21 after the corresponding antagomir transfections (respectively, Anta-590, and Anta-21). miRNA expression of other odontogenic miRNAs (miR-200c-3p, miR-429-3p, miR-183-3p, miR-325-3p, and miR-574-3p), did not change with antagomir treatments. PCR data was

normalized to the housekeeping RNA snRNA-U6, and to an off-target antagomir control (red dotted line), and plotted as Fold Change.

Supplementary Fig. 8 | Antagomir specificity in increasing expression of miRNA target genes. A selection of 15 odontogenic and morphogenetic markers (Suppl. Table 3) was used to survey the gene expression changes after antagomir transfections targeting odontogenic miRNAs. (**a**) Overview of gene expression profiles of each antagomir treatment in E13.5 mandibles explanted (24 h), and targeting, respectively: miR-429-3p (Anta-429), miR-325-3p (Anta-325), miR-590-5p (Anta-590), miR-200c-3p (Anta-200c), miR-21 (Anta-21), miR-574-3p (Anta-574), and miR-183-3p (Anta-183). Gene expression was measured by qRT-PCR, normalized to the housekeeping gene *Rps29* and to an off-target antagomir treatment (red dotted line in the graph), and plotted as Fold Change in Expression. (**b**) Analysis of the expression of selected genes and antagomir treatments from (a) revealed that the miR-590-5p predicted targets *Chd7*, *Msx1*, and *Bcl11b* were significantly upregulated after miR-590-5p antagomir treatment, together with the non-target co-regulated *Sox2* and *Bmp4*. In addition, *Nog* (a miR-200c-3p target) and *Pdcd4* (a miR-21 target) were upregulated with the respective antagomir (Anta-200c, and Anta-21) treatments. Altogether, these results indicate selective specificity of antagomir treatments in molar tooth germs.

Supplementary Fig. 9 | Trowell-type organ explant system. Preparation of Trowell-type organ cultures for mandible (containing molar germs and incisors) and tongue (containing salivary glands) explants. Metal grids fit into wells of a multiwell plate (12 wells), each designed to use 2.0 mL of culture medium.

Supplementary Fig. 10 | Dose-response for miR-590-5p mimic transfectios. miR-590-5p expression was detected by qRT-PCR in molar tooth germs after transfecting E13.5 mandibles with different concentrations of mimics (fold change in expression was calculated by normalizing the data to a housekeeping RNA snRNA-U6 and to an off-target mimic control). Doses of mimics lower than 10 nM did not show morphogenetic changes in the molar tooth germs after transfections.

Supplementary Fig. 11 | Chd7, a miR-590-5p target, and its co-factor Sox2, co-localize in the molar tooth germ. E11.5 mouse mandibles were fixed in PFA and used for histology (10 μ coronal section) and immunofluorescence analysis of Chd7 (red) and Sox2 (green) expression. E-Cadherin (blue) was used as an epithelial marker. Scale bar: 50 μ. Arrow indicates the oral epithelium region where molar tooth germ placode begins. The antibodies used for this immunofluorescence were the following: Rabbit Anti-Mouse Chd7 antibody (catalog number ab31824, Abcam), Goat Anti-Mouse Sox2 antibody (catalog number sc-17320, Santa Cruz), and Mouse Anti-Human E-Cadherin Antibody (catalog number 610181, BD Transduction Laboratories). We used the following dilutions (from original stocks): Anti-Mouse Chd7 antibody (1:500), Anti-Mouse Sox2 antibody (1:1,000), and Anti-Human E-Cadherin Antibody. For details about the IF protocol, refer to METHODS.

Supplementary Fig. 12 | Transfection of antagomirs using nanoparticles in transgenic mouse organs expressing Green Fluorescent Protein (GFP) in a *Krt14-Cre* promoter. (**a**) Overview of the E14.5 mouse expressing Green Fluorescent Protein (GFP) in the *Krt14-Cre* promoter. Ectodermal organs such as skin and hair follicles fluorescently labeled (green) while

the rest of the embryonic tissues constitutively expresses Red Fluorescent Protein (RFP). (**b**) Dissected E14.5 mouse mandibular rudiment expressing Krt14-GFP in the oral epithelium, molar tooth germs, and incisors. (**c-e**) Sagittal frozen section of E17.5 mouse mandibular rudiment expressing Krt14-GFP shows first (1) and second (2) non-erupted molar teeth, the incisor, and submandibular salivary gland (SMG). The diagram shows the sagittal orientation for sectioning (a single 10 μ section is shown in c and d). (**f-h**) Coronal frozen section of E14.5 mouse mandibular rudiment expressing Krt14-GFP shows the bell stage of molar tooth germs in the maxilla and mandible. The diagram shows the coronal orientation for sectioning (a single 10 μ coronal section is shown in f and g). (**i-j**) Krt14-GFP expressing mandibles from E13.5 mice were explanted and transfected with antagomir control (i) or antagomir miR-590-5p (j) using peptide-based nanoparticles, and used for frozen sections and analysis of molar tooth germ morphogenesis after 48 h of culture. A representative set of sequential coronal sections from the same molar tooth germ for the control (i) and miR-590-5p (j) antagomirs showed defective molar tooth morphogenesis in short-term explants. The expected bell stage morphogenesis of the molar tooth germ was lost when mandibles were transfected with antagomir targeting miR-590-5p. Scale bar: 100 μ.

Supplementary Fig. 13 | Transfections of antagomirs and mimics using nanoparticles in transgenic mouse molar tooth germs expressing Green Fluorescent Protein (GFP) in a *Shh-Cre* promoter. (**a**) Overview of the E14.5 mouse expressing Green Fluorescent Protein (GFP) in the *Shh-Cre* promoter. (**b**) Shh-GFP expression in E14.5 mouse mandible was restricted to odontogenic epithelium (molar tooth germs and incisors). (**c-d**) Shh-GFP was expressed in epithelium of E14.5 submandibular salivary glands (c) and E14.5 lungs (d). The oral epithelium

that covers the tongue also showed, to a lesser extent, Shh-GFP expression. (**e-f**) In this experiment, E14.5 molar tooth germs expressing Shh-GFP were dissected from the mandibles and explanted for 96 h using the floating filter system. Cusp formation (dotted line in blue) and crown formation (dotted line in red) were measured and quantified. The images correspond to representative pictures of molar tooth germs transfected with off-target miRNA, mimic-590-5p and antagomir targeting miR-590-5p, and the morphometric analysis of first and second molar tooth germs is shown (graphs corresponding to relative length in pixels, Y-axis, when comparing antagomir and mimic treatments to an off-target control). The results showed non-significant changes in morphogenesis of molar tooth germs in longer periods of *ex vivo* cultures, suggesting miR-590-5p developmental compensation. Scale bar: 100 μ.

Supplementary Fig. 14 | Examples of mRNA sequences containing seeding regions for miR-590-5p and types of possible seeding regions for miR-590-5p. (**a**) miRNA seeding regions are located in 3'-UTRs of corresponding mRNA targets. *Bmp4* mRNA contains a seeding region of 6merγ type that is located in the coding region of the mRNA, which disqualifies it as a binding region for miR-590-5p. (**b**) Oligonucleotide sequences for synthetic miRNA mimic and antagomir for miR-590-5p. (**c**) N-TER amphiphilic peptide sequence.

Additional References

1. Rebustini, I. T. & Hoffman, M. P. ECM and FGF-dependent assay of embryonic SMG epithelial morphogenesis: investigating growth factor/matrix regulation of gene expression during submandibular gland development. *Methods Mol. Biol.* **522,** 319–330 (2009).
2. Närhi, K. & Thesleff, I. Explant culture of embryonic craniofacial tissues: analyzing effects of signaling molecules on gene expression. *Methods Mol. Biol.* **666,** 253–267 (2010).
3. del Moral, P.-M. & Warburton, D. Explant culture of mouse embryonic whole lung, isolated epithelium, or mesenchyme under chemically defined conditions as a system to evaluate the molecular mechanism of branching morphogenesis and cellular differentiation. *Methods Mol. Biol.* **633,** 71–79 (2010).
4. Costantini, F., Watanabe, T., Lu, B., Chi, X. & Srinivas, S. Dissection of embryonic mouse kidney, culture in vitro, and imaging of the developing organ. *Cold Spring Harb Protoc* **2011,** pdb.prot5613 (2011).
5. Rebustini, I. T. *et al.* miR-200c regulates FGFR-dependent epithelial proliferation via Vldlr during submandibular gland branching morphogenesis. *Development* **139,** 191–202 (2012).
6. Rebustini, I. T. *et al.* MT2-MMP-dependent release of collagen IV NC1 domains regulates submandibular gland branching morphogenesis. *Developmental Cell* **17,** 482–493 (2009).
7. Montojo, J. *et al.* GeneMANIA Cytoscape plugin: fast gene function predictions on the desktop. *Bioinformatics* **26,** 2927–2928 (2010).
8. Zhou, L. *et al.* Integrated Profiling of MicroRNAs and mRNAs: MicroRNAs Located on Xq27.3 Associate with Clear Cell Renal Cell Carcinoma. *PLoS ONE* **5,** e15224 (2010).
9. Schmittgen, T. D. & Livak, K. J. Analyzing real-time PCR data by the comparative C(T) method. *Nat Protoc* **3,** 1101–1108 (2008).
10. Lal, A. *et al.* Capture of microRNA-bound mRNAs identifies the tumor suppressor miR-34a as a regulator of growth factor signaling. *PLoS Genet* **7,** e1002363 (2011).
